# Synthesis and Evaluation of Benzylic ^18^F-Labeled *N*-Biphenylalkynyl Nipecotic Acid Derivatives for PET Imaging of GABA Transporter 1

**DOI:** 10.1021/acschemneuro.4c00782

**Published:** 2025-01-29

**Authors:** Niels Knippenberg, Matthias Bauwens, Alexandru Florea, Soma Rudi, Olaf Schijns, Govert Hoogland, Vincent Ornelis, Ronny Mohren, Michiel Vandenbosch, Felix M. Mottaghy, Thomas J. Cleij, Kasper Eersels, Bart van Grinsven, Hanne Diliën

**Affiliations:** † Sensor Engineering Department, Faculty of Science and Engineering, Maastricht University, 6200 MD Maastricht, The Netherlands; ‡ Institute of Nutrition and Translational Research in Metabolism (NUTRIM), Maastricht University, 6200 MD Maastricht, The Netherlands; § Department of Radiology and Nuclear Medicine, Maastricht University Medical Centre+ (MUMC+), 6229 HX Maastricht, The Netherlands; ∥ School for Cardiovascular Diseases (CARIM), Maastricht University Medical Centre+ (MUMC+), 6229 HX Maastricht, The Netherlands; ⊥ Department of Nuclear Medicine, University Hospital Aachen, RWTH Aachen University, 52074 Aachen, Germany; # Maastricht Science Programme (MSP), Faculty of Science and Engineering, Maastricht University, 6200 MD Maastricht, The Netherlands; ∇ Department of Neurosurgery, Maastricht University Medical Centre+ (MUMC+), 6229 HX Maastricht, The Netherlands; ○ Mental Health and Neuroscience (MHeNS) Research Institute, Maastricht University, 6200 MD Maastricht, The Netherlands; ◆ Academic Center for Epileptology (ACE), Maastricht University Medical Centre+ (MUMC+), 6229 HX Maastricht, The Netherlands; ¶ The Maastricht MultiModal Molecular Imaging (M4I) Institute, Division of Imaging Mass Spectrometry (IMS), Maastricht University, 6229 ER Maastricht, The Netherlands

**Keywords:** GABA, GABA transporter, radioligand, positron emission tomography, fluorine-18

## Abstract

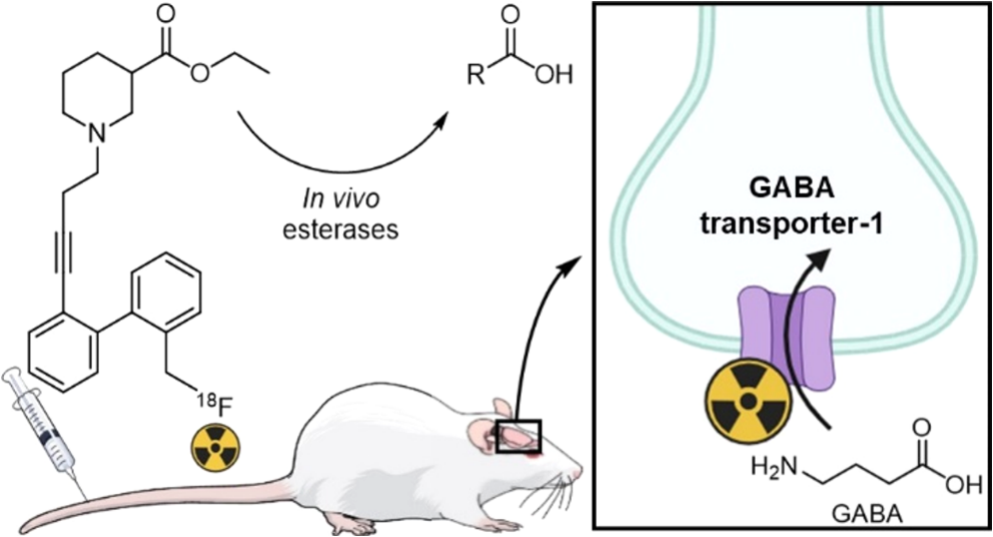

As the main inhibitory neurotransmission system, the GABAergic system poses an interesting yet underutilized target for molecular brain imaging. While PET imaging of postsynaptic GABAergic neurons has been accomplished using radiolabeled benzodiazepines targeting the GABA_A_ receptor, the development of presynaptic radioligands targeting GABA transporter 1 (GAT1) has been unsuccessful thus far. Therefore, we developed a novel GAT1-addressing radioligand and investigated its applicability as a PET tracer in rodents. We selected a lipophilic nipecotic acid scaffold that is known to bind selectively to GAT1 as the basis for our radioligand. To obtain the desired candidate radiotracer **[**^**18**^**F]4**, ester-protected radioligands **[**^**18**^**F]11a-b** were synthesized through aliphatic nucleophilic radiofluorination of the respective bromo-precursors, after which chemical deprotection was attempted using various conditions. Because these deprotections were unsuccessful, it was evaluated whether the ethyl ester **[**^**18**^**F]11a** could function as a prodrug and afford the active radioligand **[**^**18**^**F]4** after *in vivo* ester hydrolysis by esterases. Unfortunately, PET imaging studies in a rat model using **[**^**18**^**F]11a** showed no brain uptake of the radiotracer. Instead, significant uptake of radioactivity was observed in the liver and bones, the latter being caused by radiodefluorination of the PET tracer. Since the PET tracer developed in this study was found to be unstable, further efforts should investigate the development of a more stable GAT1-addressing PET tracer without the potential labile benzyl fluoride moiety. Moreover, as the still intact fraction of the radiotracer did not cross the BBB, options other than the prodrug approach should be considered to increase the BBB permeability of future GAT1 radioligands.

## 1 Introduction

**Figure 1:**
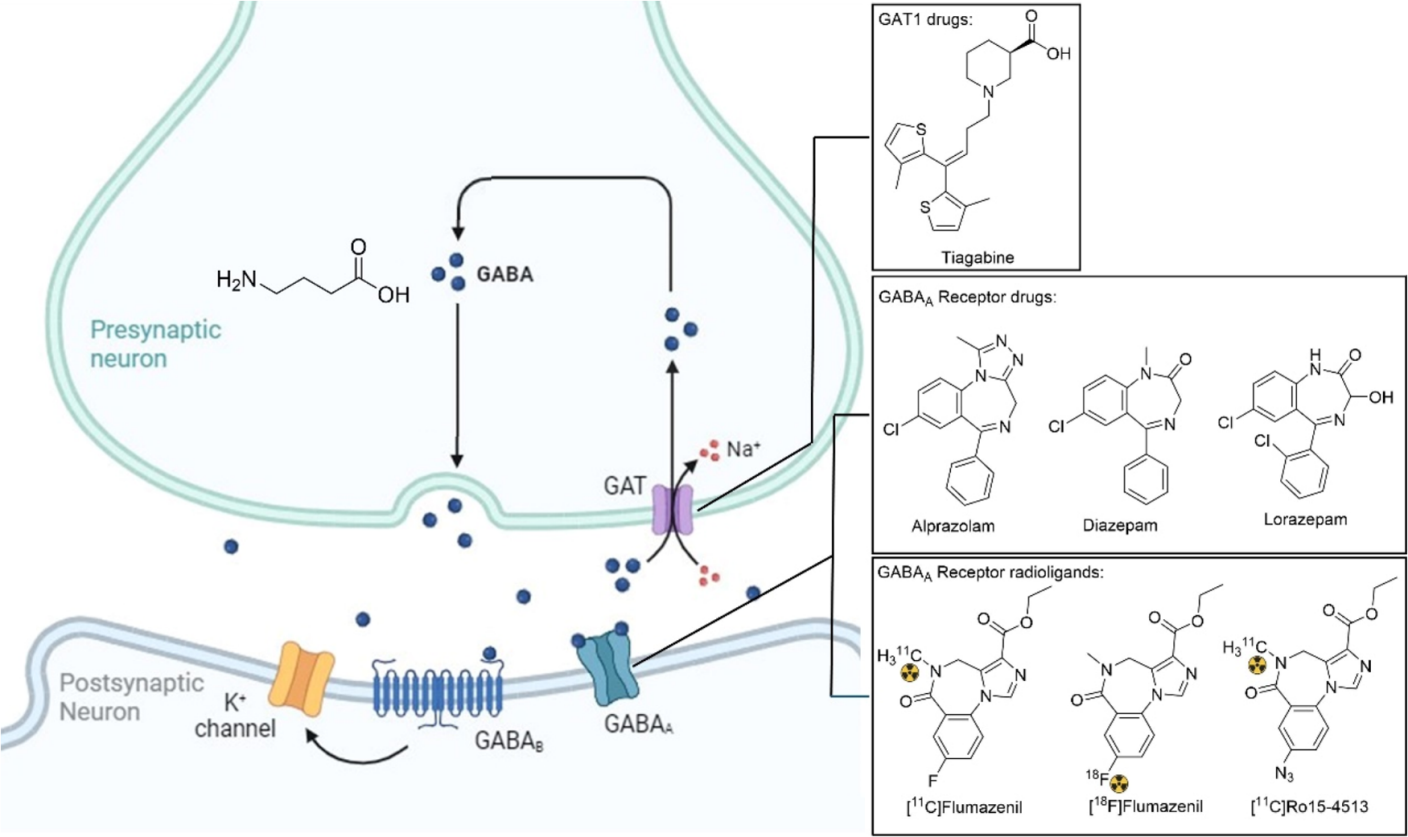
Schematic overview of the GABAergic synapse and selected drugs and radioligands targeting the GABAergic system.

The amino acid γ-aminobutyric acid (GABA) is the principal inhibitory neurotransmitter in the mammalian central nervous system (CNS). Dysfunctions of the GABAergic system (Figure 1) are associated with the pathogenesis of various neurological disorders, such as epilepsy, schizophrenia, Parkinson’s disease, and Alzheimer’s disease.^1^ Therefore, several drugs modulating the GABAergic system are prescribed to manage these disorders. These include benzodiazepines (e.g., alprazolam, diazepam, and lorazepam) that activate the GABA_A_ receptor and tiagabine, which selectively inhibits the neuronal GABA transporter 1 (GAT1).^2^ Given the importance of the GABAergic system in various neurological diseases, the development of noninvasive imaging methods of the GABAergic system is highly desired in order to better understand the pathogenesis of these CNS disorders and enable earlier diagnosis and treatment options. However, the clinically available radioligands that are able to image GABAergic neurons are rather limited and solely consist of benzodiazepine derivatives (e.g., [^11^C]flumazenil, [^18^F]flumazenil, and [^11^C]Ro15-4513; Figure 1), that only target the GABA_A_ receptor.^2,3^ Since GABA_A_ receptors are mostly located on the postsynaptic GABAergic neurons, no information about presynaptic GABAergic neurons can be obtained using the currently available radioligands.

The GABA transporter (GAT) could function as a target for radioligands intended for the visualization of presynaptic GABAergic neurons. Especially GAT1 has been recognized as a promising target for the development of radioligands,^5^ as itis the most abundant transporter of the four GAT isoforms,^4^ and is mainly present on presynaptic GABAergic neurons. GAT1-addressing radioligands would be especially beneficial for patients with temporal lobe epilepsy, as several lines of evidence suggest these patients have reduced GAT expression.^6−10^ Moreover, the effect of GAT1-modulating drugs has also been studied on anxiety disorders, depression, sleep apnea, and schizophrenia and has shown promising results in preclinical studies.^11^ As such, noninvasive imaging of GAT1 could help to more precisely understand the role of GAT1 in these disorders as well.

**Figure 2:**
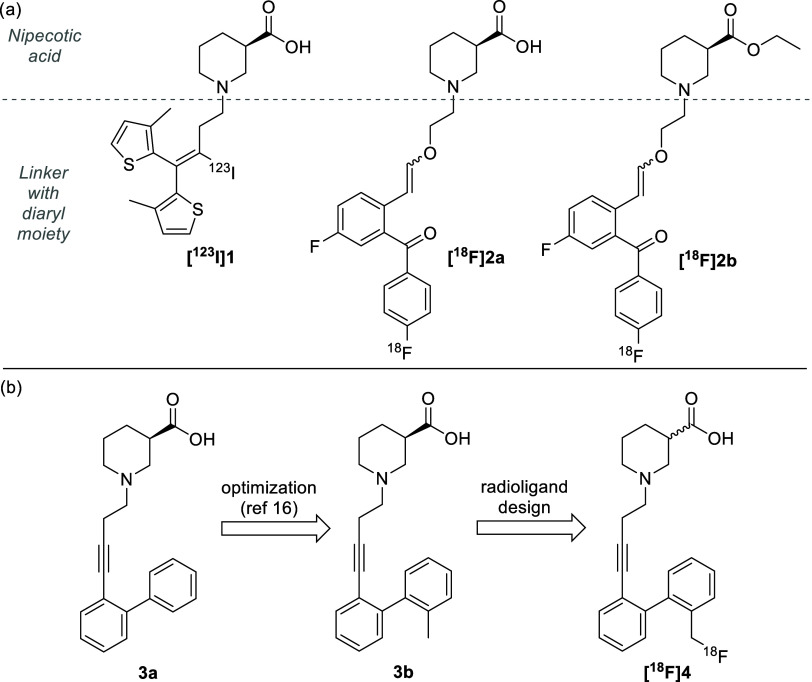
(a) Examples of previously synthesized GAT1-addressing radioligands and (b) design of radioligand **[**^**18**^**F]4** developed in this study.

To enable noninvasive GAT imaging, several research attempts have been made to develop GAT1-addressing radioligands.^5^ Most of these compounds are based on nipecotic acid, which is a cyclic analogue of GABA and is required for efficient binding into the GAT1-binding site (Figure 2a). In a further common design, the nipecotic acid moiety is *N*-alkylated with a lipophilic moiety consisting of a linker and a diaryl system to facilitate passage through the blood–brain barrier (BBB).^5^ This will result in increased brain uptake, which is required to reach the GAT1 target located in the brain. Unfortunately, the radioligands that have been developed thus far have been mostly unsuccessful. For example, [^123^I]iodotiagabine **[**^**123**^**I]1** failed to enter the brain, in part due to possible deiodination, and also the recently developed radioligand **[**^**18**^**F]2a** exhibited poor BBB permeability.^12,13^ In an attempt to rationalize the poor brain uptake, the ethyl ester of the latter radioligand (i.e., **[**^**18**^**F]2b**) was also evaluated for its BBB permeability.^13^ This compound **[**^**18**^**F]2b** showed significant brain uptake in rhesus monkeys as the ester moiety can help the radiotracer cross the BBB, after which the ester can be converted into the active carboxylic acid **[^18^F]2a** by esterases in the brain.^14,15^

In view of the lack of appropriate GAT1 radioligands, a new positron emission tomography (PET) radiotracer targeting the GABA transporter was designed in this study. This radioligand is based on a line of GAT1 selective inhibitors developed by Wanner and co-workers featuring a nipecotic acid moiety, alkynyl spacer, and biphenyl moiety (i.e., scaffold **3a**, Figure 2b).^16^ Structure–activity relationships revealed that the introduction of a halide or methyl substituent at the 2′-position of the distal phenyl moiety significantly increases the GAT1 affinity, with compound **3b** being one of the most potent GAT1 inhibitors with a carbon linker known to date (p*K*_i_ = 8.32, pIC_50_ = 7.60 for mGAT1). It was decided to use this compound as the basis for our radioligand design for several reasons. First, the excellent potency and selectivity for GAT1 inhibition provide a scaffold that binds well into GAT1, which is essential for a radioligand. Moreover, the absence of heteroatoms in the linker prevents the introduction of polar moieties except for the necessary nipecotic acid group. Therefore, the scaffold is more hydrophobic than for example compound **[**^**18**^**F]2a** (compare predicted log *D*_7.4_ values: **[**^**18**^**F]2a** 1.49; **[**^**18**^**F]4** 1.94)^17^ and has a higher chance of passing through the BBB. Finally, the methyl substituent provides an easy handle to introduce a radioisotope on the synthetically accessible benzylic position. Fluorine-18 was used as radioisotope, considering its favorable half-life (109.7 min) and excellent physicochemical properties limiting the steric perturbations after substitution.^18^ This is in strong contrast to the more bulky iodine-123 for example.

A potential risk regarding this strategy could be that while the reactive benzylic position allows for easy labeling it may also aid defluorination, as some [^18^F]benzyl fluoride radioligands are reported to suffer from radiodefluorination.^19−21^ However, with other [^18^F]benzyl fluorides being reported to be stable,^22−24^ the rate of defluorination seems to be dependent on the exact substituents of the benzene ring and can be difficult to predict.^23^ As such, we aimed to introduce a fluorine-18 radionuclide on the benzylic position through nucleophilic radiofluorination, which should afford radioligand **[**^**18**^**F]4** (Figure 2b). In this paper, we describe the synthesis of compound **[**^**18**^**F]4** and its ester derivatives as novel GAT1-addressing radioligands and investigate their applicability as PET tracers in rodents.

## 2 Results and Discussion

### 2.1 Synthesis of Radiolabeling Precursors **10a** and **10b**

**Scheme 1:**
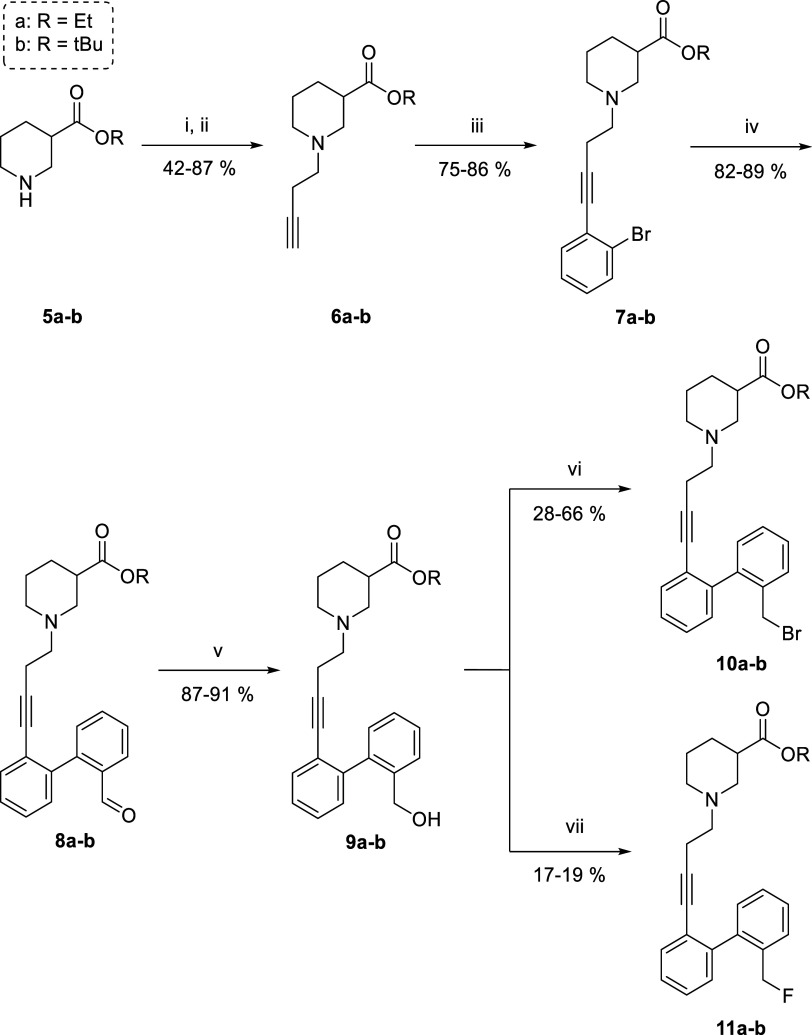
Synthesis of Radioligand Precursors and References Reagents and conditions: (i) 3-butynyl tosylate (1.0 equiv), neat, rt, on.; (ii) 4-bromo-1-butyne (1.0 equiv), K_2_CO_3_ (2.0 equiv), NaI (0.2 equiv), acetone, rt, on.; (iii) 2-bromoiodobenzene (1.0 equiv), Pd(dppf)Cl_2_ (1 mol %), CuI (4 mol %), K_3_PO_4_ (3.0 equiv), dioxane/H_2_O (1:1), 60 °C, on.; (iv) 2-formylphenylboronic acid (1.5 equiv), Pd_2_(dba)_3_·CHCl_3_ (1 mol %), SPhos (4 mol %), K_3_PO_4_ (2.0 equiv), dioxane/H_2_O (1:1), 60 °C, on.; (v) NaBH_4_ (2.0 equiv), EtOH for **8a** and *^t^*BuOH for **8b**, 0 °C, 2 h; (vi) CBr_4_ (1.3 equiv), polymer-bound PPh_3_ (2.6 equiv), dichloromethane (DCM), 3 h, rt; (vii) DAST (2.0 equiv), CsF (0 or 2.0 equiv), DCM, 0 °C, 3 h.

For the synthesis of the fluorine-18 labeled lead compound **[**^**18**^**F]4**, an S_N_2 strategy was envisioned starting from brominated precursor **10** (Scheme 1). Since nucleophilic fluorinations with [^18^F]fluoride using unprotected carboxylic acids are known to be challenging due to the ability of [^18^F]fluoride to form hydrogen fluoride with acidic protons,^25,26^ it was decided to protect the nipecotic acid moiety. To this end, both base-labile ethyl ester precursor **10a** and acid-labile *tert*-butyl ester precursor **10b** were investigated.

The ethyl ester precursor **10a** was accessed through a linear reaction pathway starting from commercially available racemic ethyl nipecotate **5a** (Scheme 1). The alkyne linker was then attached to this protected nipecotic acid building block through an S_N_2 reaction with 3-butynyl tosylate to access the terminal alkyne **6a**.^16^ This allowed for a stepwise construction of the biphenyl moiety using consecutive Sonogashira and Suzuki-Miyaura cross-coupling reactions, leveraging the reactivity difference of the two halides in *ortho*-bromoiodobenzene.^16^ The resulting aldehyde **8a** was then subjected to a NaBH_4_ reduction to afford alcohol **9a** in excellent yields.

To convert the alcohol into a good leaving group, several tosylation and deoxyhalogenation reactions were attempted using the model system biphenyl-4-methanol (see Supporting Information(27,28)). Since classic tosylation conditions using TsCl and Et_3_N or pyridine led to chlorination instead of tosylation,^29^ it was decided to perform an Appel reaction to obtain the brominated compound. To ease purification efforts for the brominated precursor **10a**, it was decided to perform the Appel reaction using polymer-supported PPh_3_ (PS–PPh_3_).^30^ Using this procedure, it was possible to obtain brominated precursor **10a** in modest yield. Since long-term storage of the brominated precursor was found to be difficult due to degradation (vide supra), the compound was freshly prepared a couple of days before radiofluorination.

Because we were also interested in synthesizing *tert*-butyl ester precursor **10b**, we explored transesterification of **7a** to **7b** using potassium *tert*-butoxide. However, this led to base-catalyzed alkyne-to-allene isomerization (see Supporting Information).^31,32^ Therefore, the *tert*-butyl precursor **10b** was accessed through the same linear pathway as the ethyl ester precursor **10a** starting from *tert*-butyl nipecotate **5b**, which was prepared from nipecotic acid as previously described.^33,34^

Besides the brominated precursors **10a**–**b**, it was also important to obtain the nonradioactive analogues **11a**–**b** as reference standards to validate the formation of **[**^**18**^**F]11a–b** during radiosynthesis. To that end, DAST-mediated deoxyfluorination was explored as a means of accessing the fluorinated references. While this reaction proceeded smoothly for the model system biphenyl-4-methanol, translation to the alcohol **9a**–**b** generated a side-product that was assumed to result from a 6-*exo*-*dig* cyclization with the alkyne (see Supporting Information(35−41)).^42^ To our delight, the addition of CsF lowered the amount of side-product formed, which could then mostly be separated to afford the fluorinated references **11a**–**b**.

### 2.2 Stability Tests of Radiolabeling Precursors **10a**–**b**

**Figure 3:**
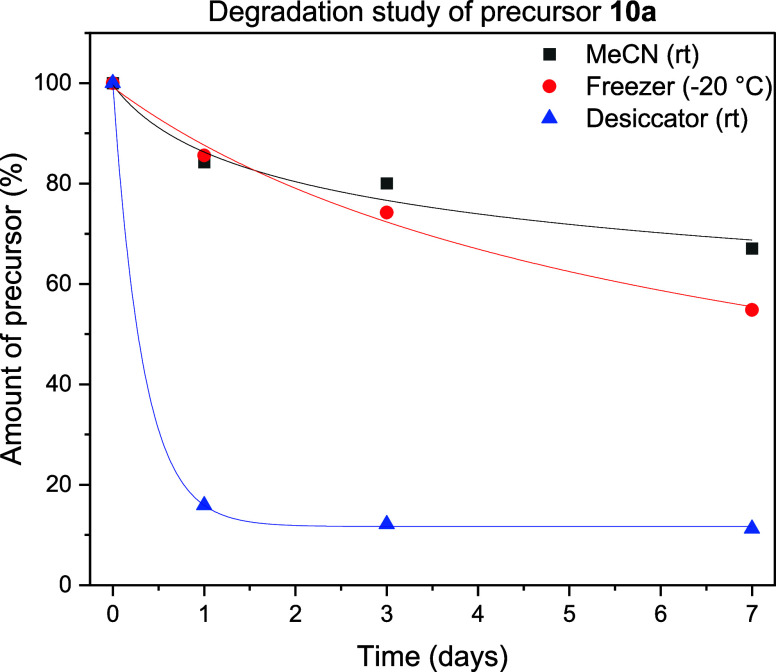
Degradation study of precursor **10a** under different storage conditions; black: MeCN solution (1.5 mg/mL, rt); red: freezer (−20 °C); blue: desiccator under vacuum (rt). After synthesis, product **10a** was divided into batches of 1 mg, which were stored as indicated. Upon sample preparation, MeCN (670 μL) and internal standard solution (830 μL, 75 μM 2-nitrobenzoic acid) were added and the samples were measured by liquid chromatography–mass spectrometry (LC-MS). The amount of precursor was calculated using the integration of the PDA signal of **10a** as a percentage of the value at day 0.

Since it was discovered that the brominated radiolabeling precursors **10a**–**b** degraded over time, a survey of storage conditions was conducted using compound **10a** to study the degradation. It was found that the compound degraded within days when stored in its pure form in the desiccator but was relatively stable for at least a week in the freezer or preferably as MeCN solution (Figures 3 and S12). Therefore, the radiolabeling precursors were freshly prepared a couple of days before radiofluorination and stored in MeCN. Proposed degradation products that were observed during these studies included the resonance-stabilized carbocation [M–Br]^+^ (374 *m*/*z*) resulting from cleavage of the bromide and its acetonitrile adduct (415 *m*/*z*). Therefore, it seems that the cleavage of the carbon-bromide bond and the formation of the stabilized carbocation is a likely mechanism for further degradation of the precursor. In order to obtain more information on the bond cleavage, studies were undertaken in which the dissolved precursor was mixed with the radical scavenger TEMPO (0.1 equiv) (Figure S13). The addition of TEMPO did not significantly change the amount of degradation after 7 days (compared to precursor in MeCN without TEMPO), indicating that it is not likely for the degradation to proceed through a radical pathway. Moreover, it was found that the addition of water (10 v/v%) did not influence the degradation either, indicating that no direct substitution of the benzylic bromide moiety occurred in neutral aqueous conditions.

### 2.3 Radiosynthesis of Compound **[**^**18**^**F]4**

**Scheme 2:**
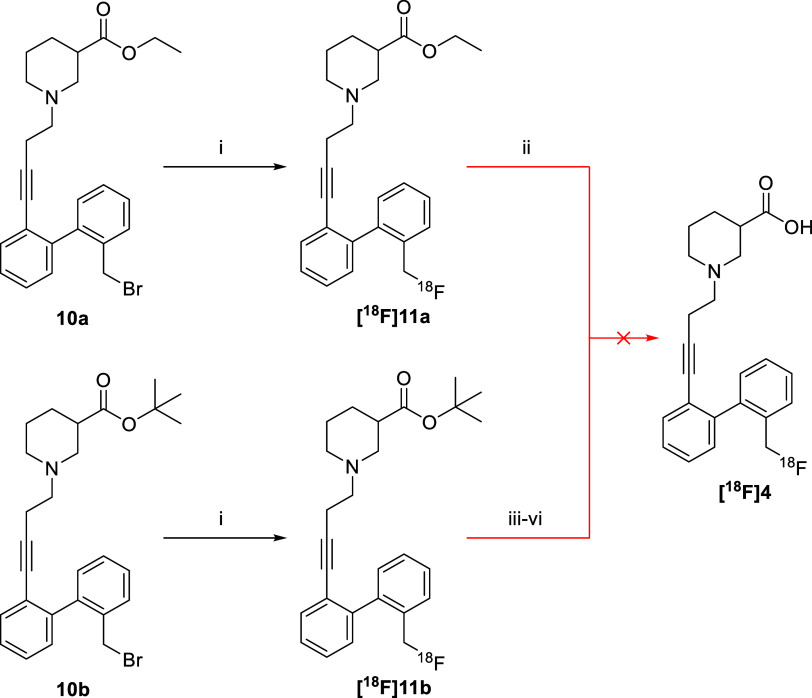
Attempted Synthesis of Radioligand **[**^**18**^**F]4** from Ester-Protected Precursors **10a**–**b** Reagents and conditions: (i) [^18^F]F^–^/K222/K_2_CO_3_ complex, MeCN, 105 °C, 15 min; (ii) aq. 0.01 M NaOH, rt, 5 min; (iii) 2 M aq. HCl, 105 °C, 10 min; (iv) formic acid, 50 °C, 15 min; (v) trifluoroacetic acid (TFA), rt, 15 min; (vi) TFA, 105 °C, 20 min.

Having synthesized the brominated radiolabeling precursors **10a**–**b**, we proceeded with the synthesis of the fluorine-18 labeled compound **[**^**18**^**F]11a** by subjecting the freshly prepared (ideally a couple of days before radiolabeling) bromo-precursor **10a** to standard aliphatic nucleophilic fluorination conditions ([^18^F]F^–^/K222/K_2_CO_3_ in MeCN, 105 °C, 15 min) (Scheme 2). Given the instability of the bromo-precursor **10a**, only modest yields of the radiolabeled product **[**^**18**^**F]11a** could be obtained (3–5% according to high-performance liquid chromatography (HPLC), see Supporting Information). Nevertheless, saponification of the ethyl ester was then attempted (200 μL aq 0.01 M NaOH, 100 °C, 20 min) but led to complete degradation of the product (HPLC spectra, see Supporting Information). When these deprotection conditions were employed on the nonradioactive analogue **11a**, no degradation was observed, meaning the difference in reactivity is likely attributed to the different scale and stoichiometry of the reaction.

Besides the base-mediated deprotection, it was attempted to perform the deprotection under acidic conditions using the acid-labile *tert*-butyl protecting group. To that end, compound **[**^**18**^**F]11b** was prepared in a fashion similar to that of compound **[**^**18**^**F]11a**, after which several deprotection methods were investigated. Treatment of the *tert*-butyl ester with aqueous 2 M HCl led to the degradation of the product and the formation of free fluoride. Due to the problems with the deprotection in acidic aqueous conditions, the following deprotection attempts were performed in water-free conditions using formic acid and TFA. Unfortunately, neither of these options cleanly deprotected **[**^**18**^**F]11b**, as the remaining ester could still be found after the deprotection among several more lipophilic degradation products (HPLC spectra, see Supporting Information). Because the deprotection of compounds **[**^**18**^**F]11a–b** could not be achieved despite many attempts, further efforts were refocused on investigating whether the protected ethyl ester precursor **[**^**18**^**F]11a** could be employed as a prodrug.

### 2.4 Ethyl Ester Prodrug Approach

It is known that nipecotic acid alkyl esters readily cross the blood–brain barrier and can be hydrolyzed to give nipecotic acid once in the brain.^14,15^ Thus, instead of synthesizing the carboxylic acid derivative **[**^**18**^**F]4** as attempted above, one could employ the ethyl ester precursor **[**^**18**^**F]11a** which we successfully obtained. It is expected that this ester analogue can then be converted into the active carboxylic acid derivative **[**^**18**^**F]4***in vivo* through the action of esterases. Combined with the fact that **[**^**18**^**F]2b** (Figure 1) exhibits uptake in the brain of rhesus monkeys,^13^ it was decided to continue studies using **[**^**18**^**F]11a** and evaluate whether this ethyl ester could function as a prodrug.

To that end, **[**^**18**^**F]11a** was purified using semipreparative HPLC and reformulated using a C18 cartridge to give 2.9 MBq as a final formulated dose (0.52% RCY (decay-corrected)). Coinjection of the radioactive product after purification with the reference standard **11a** confirmed the isolated radiotracer to be **[**^**18**^**F]11a** (Figures S29–S30). Unfortunately, [^18^F]fluoride was also detected in these coinjections, indicating that the tracer suffered from radiodefluorination upon prolonged contact with the acidic aqueous HPLC eluent. The radiochemical purity (RCP) of the tracer was found to be 63% after 45 min and 56% after 85 min. Moreover, the molar activity was estimated to be 3.22 MBq/μmol (Supporting Information).

**Figure 4:**
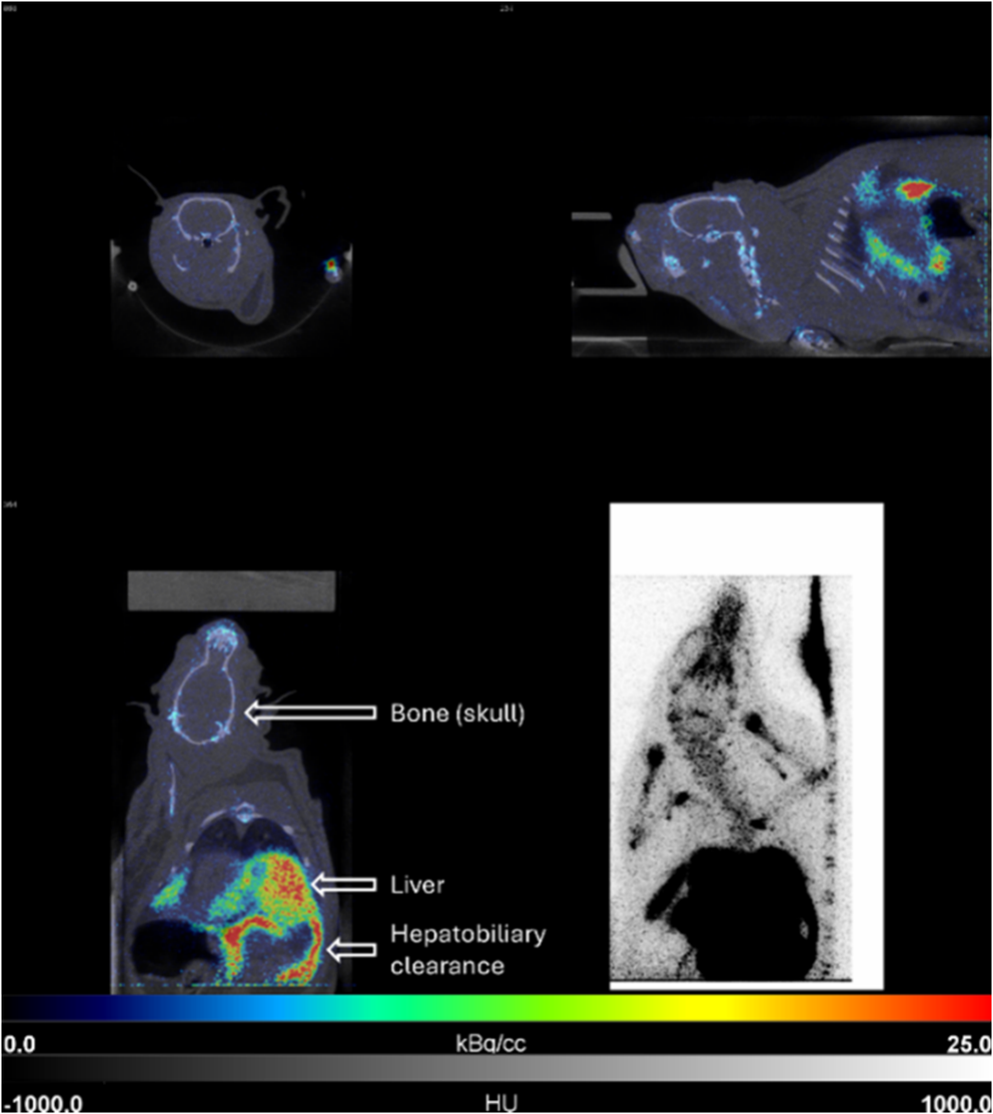
Coronal, sagittal, and transversal fused PET/CT image after injection of **[**^**18**^**F]11a**. The image is a static recon of the full 45 min PET scan. PET and CT scales are shown at the bottom of the image. No brain uptake of the radiotracer is observed.

After reformulation, preliminary PET imaging was conducted in a rodent, which unfortunately showed no brain uptake of the PET tracer (Figure 4). Instead, a significant uptake of radioactivity was shown in the liver and to a smaller extent in the bones. The radioactivity uptake in the liver can be explained by the clearance pathway of the lipophilic product via this organ. Meanwhile, radioactivity uptake into bones, including the skull, was observed due to the radiodefluorination of the PET tracer and the binding of the resulting [^18^F]fluoride to the hydroxyapatite in bones.^43,44^ The radiodefluorination seems to be caused by the potentially labile benzyl fluoride moiety, which was seen to be unstable in basic or acidic aqueous media *ex vivo* and also suffered from further *in vivo* defluorination after the previously generated [^18^F]fluoride was separated during reformulation.^20,22^ Metabolism studies of other [^18^F]benzyl fluorides indicate that this *in vivo* radiodefluorination can occur via hydroxylation of the reactive benzylic carbon, after which [^18^F]fluoride can be liberated through the formation of an aldehyde.^19,20^

This *in vivo* defluorination would partially explain the lack of brain uptake of **[**^**18**^**F]11a**, as the amount of intact radioligand is decreased. However, since not all of the injected radioligand suffered from radiodefluorination, other reasons to explain the inability of the still intact tracer to cross the BBB need to be evaluated. Likely reasons for the BBB impermeability include poor pharmacokinetics or hydrolysis of the ethyl ester in plasma and the inability of the resulting carboxylic acid to cross the BBB. Evidence for the latter can also be found in the literature that studied ethyl ester **[**^**18**^**F]2b** and found that this compound exhibited brain uptake in rhesus monkeys but not in rats.^13^ The authors hypothesized that the difference between the species could be caused by faster hydrolysis of the ester in rat plasma,^45,46^ which might also have happened to our radiotracer **[**^**18**^**F]11a**. Less likely reasons to explain the BBB-impermeability of the radioligand **[**^**18**^**F]11a** could include efflux transport from the brain (although **[**^**18**^**F]2a** is shown to be not affected by P-glycoprotein (Pgp) efflux transport)^13^ or the inability of the prodrug construct to passively cross the BBB (which is unlikely given the brain uptake of ethyl nipecotate and other nipecotic acid alkyl esters).^14,15^

## 3 Conclusions

In this work, we developed the synthesis of a fluorine-18 labeled radioligand based on a previously described scaffold with high affinity and selectivity for the GAT1 transporter. Even though our final goal was the synthesis of the deprotected carboxylic acid derivative **[**^**18**^**F]4**, multiple deprotection attempts failed. We then investigated if the protected ethyl ester precursor **[**^**18**^**F]11a** could be employed as a radioligand in a prodrug approach. Synthesis of this radiolabeled ethyl ester prodrug could be achieved, although it was found to suffer from radiodefluorination after prolonged contact with the acidic aqueous HPLC eluent. Subsequent *in vivo* imaging in a rat model revealed that the compound showed no uptake in the brain, and radiodefluorination resulted in uptake in the bones. The observed radiodefluorination can be caused by the reactivity of the potentially labile fluoromethylbiphenyl moiety. Therefore, further work will focus on the development of more stable analogues that do not contain the labile benzyl fluoride moiety. Moreover, it was found that protecting the nipecotic acid with an ethyl ester did not lead to uptake of the prodrug into the rat brain, indicating that other approaches than the usage of prodrugs would be required to increase BBB permeability of nipecotic acid-derived GAT1 radioligands. A promising solution that has been proposed previously is the use of carboxylic acid bioisosteres.^5,13,33^ In this approach, the carboxylic acid moiety is replaced by a group that has similar biological properties but can lead to an increase in lipophilicity. Fortunately, many carboxylic bioisosteres are known^47−49^ and there is some precedent in their use for GAT1 inhibitors,^5^ making this a favorable option for further research.

## 4 Methods

### 4.1 Synthesis of Precursors and Reference Standards

#### 4.1.1 General Considerations

Reagents were purchased from commercial suppliers and used as received unless noted otherwise. Moisture and air-sensitive reactions were carried out under a nitrogen environment using standard Schlenk techniques. Thin layer chromatography was performed using TLC Silica gel 60 F_245_ TLC plates (Merck) and visualized using UV-light or potassium permanganate staining. Flash column chromatography was performed using a Biotage Isolera One Flash Chromatograph and prepacked silica cartridges (Biotage Sfär Silica D Duo 60 μm).

FTIR measurements were performed on a Shimadzu IRSpirit Fourier Transform Infrared Spectrometer equipped with a QATR-S single reflection ATR accessory. NMR measurements were carried out using a Bruker Ultrashield 300 MHz NMR spectrometer or a JEOL 400YH 400 MHz NMR spectrometer. The solvent used was CDCl_3_, unless noted otherwise. Chemical shift values are reported in ppm and referenced to the residual solvent signal.^50^ Data are reported as follows: chemical shift, multiplicity (s = singlet, d = doublet, t = triplet, q = quartet, p = pentet, dd = doublet of doublets, dt = doublet of triplets, td = triplet of doublets, m = multiplet), coupling constant (Hz), and integration. LC-MS analyses were performed on a Shimadzu LC-MS 2020 mass spectrometer equipped with an SPD-M40 diode array detector and an ELSD-LT III evaporative light scattering detector. Separation was achieved by reverse liquid partition chromatography using a Shimadzu Shim-pack GIST C18-AQ column (1.9 μm, 2.1 mm × 100 mm). Gradient elution was performed using water and acetonitrile, both with 0.1% formic acid. In the first two minutes, a mixture of 95% water and 5% acetonitrile was used as eluent, after which the percentage of acetonitrile was increased to 95% over 6 min. Then a mixture of 5% water and 95% acetonitrile was used for 1 min, after which the eluent was switched back to the first mixture for the remaining 3 min. The flow rate was 0.40 mL/min for the whole elution program. The column oven temperature was set at 40 °C. Mass spectra were obtained using electrospray ionization (ESI), and scanning was done over the range *m*/*z* 75–1000. HRMS-ESI spectra were obtained with an Exploris 480 spectrometer (Thermo Scientific) at 480.000 K mass resolution.

#### 4.1.2 Synthesis of Ethyl 1-(But-3-yn-1-yl)piperidine-3-carboxylate **(6a)**

Compound **6a** was synthesized according to a procedure developed by Wanner and co-workers.^16^ The atmosphere in an oven-dried round-bottomed flask was changed to N_2_. Afterward, but-3-yn-1-yl-4-methylbenzenesulfonate (2.5 mL, 11.1 mmol, 1.0 equiv) and ethyl nipecotate **5a** (4.3 mL, 27.9 mmol, 2.5 equiv) were added, and the mixture was stirred for 24 h at room temperature. After the reaction time had elapsed, DCM (100 mL) and water (100 mL) were added, and the product was extracted with DCM. The combined organic layers were washed with brine and dried over MgSO_4_, after which the solvent was removed *in vacuo* to obtain the crude product. The crude product was purified by flash column chromatography (eluting with hexane/EtOAc 95/5 to 70/30) to yield product **6a** as a colorless oil (2.0 g, 9.6 mmol, 87%). *R*_f_ = 0.50 (hexane/EtOAc 2/1); ^1^H NMR (300 MHz, CDCl_3_) δ = 4.13 (q, *J* = 7.13 Hz, 2H), 2.97 (d, *J* = 11.0 Hz, 1H), 2.76 (d, *J* = 11.2 Hz, 1H), 2.63–2.50 (m, 3H), 2.37 (td, *J* = 7.5, 2.4 Hz, 2H), 2.23 (t, *J* = 10.6 Hz, 1H), 2.08 (td, *J* = 10.8, 2.8 Hz, 1H), 1.99 (t, *J* = 2.6 Hz, 1H), 1.94–1.88 (m, 1H), 1.78–1.68 (m, 1H), 1.65–1.52 (m, 1H), 1.52–1.36 (m, 1H), 1.25 (t, *J* = 7.1 Hz, 3H); ^13^C NMR (75 MHz, CDCl_3_) δ = 173.9, 82.6, 69.0, 60.1, 57.2, 54.9, 53.3, 41.6, 26.7, 24.4, 16.6, 14.1; FTIR ν = 3294, 2943, 2808, 2120, 1728, 1470, 1447, 1369, 1308, 1215, 1180, 1153, 1134, 1099, 1030 cm^–1^; ESI-MS *m*/*z* 210 [M + H]^+^. The spectral data are in accordance with literature.^16,51^

#### 4.1.3 Synthesis of *tert*-Butyl 1-(But-3-yn-1-yl)piperidine-3-carboxylate **(6b)**

*tert*-Butyl nipecotate **5b** was synthesized from nipecotic acid as described previously,^33^ according to a modified procedure developed by Hughes and co-workers.^34^ Compound **6b** was then synthesized according to a procedure developed by Sørensen and co-workers.^52^ To a solution of *tert*-butyl nipecotate **5b** (4.9 g, 26.5 mmol, 1.0 equiv) in acetone (160 mL) were added 4-bromobut-1-yne (2.5 mL, 26.5 mmol, 1.0 equiv), K_2_CO_3_ (7.3 g, 52.9 mmol, 2.0 equiv), and NaI (0.79 g, 5.3 mmol, 0.2 equiv). The reaction mixture was stirred at room temperature overnight, after which it was filtered to remove the base and salts. The filtrate was concentrated *in vacuo* to obtain the crude product. The crude product was purified by flash column chromatography (eluting with hexane/EtOAc 85/15 to 70/30) to yield the product **6b** as a yellow oil (2.62 g, 11.0 mmol, 42%). *R*_f_ = 0.29 (hexane/EtOAc 2/1); ^1^H NMR (400 MHz, CDCl_3_) δ = 2.95 (dd, *J* = 11.1, 3.3 Hz, 1H), 2.76 (d, *J* = 11.1 Hz, 1H), 2.62–2.55 (m, 2H), 2.44 (tt, *J* = 10.8, 3.8 Hz, 1H), 2.37 (td, *J* = 7.5, 2.6 Hz, 2H), 2.14 (t, *J* = 10.7 Hz, 1H), 2.03 (td, *J* = 11.1, 2.9 Hz, 1H), 1.96 (t, *J* = 2.7 Hz, 1H), 1.94–1.85 (m, 1H), 1.70 (dt, *J* = 13.3, 3.6 Hz, 1H), 1.60–1.48 (m, 1H), 1.43 (s, 9H), 1.35 (td, *J* = 12.1, 3.9 Hz, 1H); ^13^C NMR (101 MHz, CDCl_3_) δ 173.7, 83.0, 80.3, 69.1, 57.5, 55.4, 53.5, 42.8, 28.2, 27.1, 24.7, 16.7; FTIR ν = 3310, 2976, 2904, 2811, 2120, 1723, 1469, 1453, 1393, 1367, 1313, 1242, 1147, 1102, 848, 629 cm^–1^; ESI-HRMS *m*/*z* [M + H]^+^ calcd for C_14_H_24_NO_2_^+^: 238.1802, found 238.1803.

#### 4.1.4 Synthesis of Ethyl 1-(4-(2-Bromophenyl)but-3-yn-1-yl)piperidine-3-carboxylate **(7a)**

Compound **7a** was synthesized according to a modified procedure developed by Wanner and co-workers.^16^ Pd(dppf)Cl_2_ (70 mg, 0.096 mmol, 1 mol %), CuI (73 mg, 0.38 mmol, 4 mol %), and K_3_PO_4_ (6.1 g, 28.7 mmol, 3.0 equiv) were introduced into a round-bottomed flask, after which the atmosphere was changed to N_2_. Then the alkyne **6a** (2.0 g, 9.56 mmol, 1.0 equiv) in degassed dioxane (10 mL) and degassed water (10 mL) were added. After the addition of 2-bromoiodobenzene (1.2 mL, 9.56 mmol, 1.0 equiv), the mixture was stirred at 60 °C overnight. After cooling to room temperature, DCM (100 mL) and water (100 mL) were added, and the product was extracted with DCM. The combined organic layers were washed with brine and dried over MgSO_4_, after which the solvent was removed *in vacuo* to obtain the crude product. The crude product was purified by flash column chromatography (eluting with hexane/EtOAc 95/5 to 80/20) to yield the product **7a** as a yellow to light brown oil (3.0 g, 8.21 mmol, 86%). *R*_f_ = 0.23 (hexane/EtOAc 4/1); ^1^H NMR (300 MHz, CDCl_3_) δ = 7.54 (dd, *J* = 8.0, 0.9 Hz, 1H), 7.41 (dd, *J* = 7.7, 1.6 Hz, 1H), 7.22 (td, *J* = 7.6, 1.1 Hz, 1H), 7.11 (td, *J* = 7.8, 1.7 Hz, 1H), 4.12 (q, *J* = 7.1 Hz, 2H), 3.06 (dd, *J* = 11.1, 3.3 Hz, 1H), 2.83 (d, 11.1 Hz, 1H), 2.77–2.72 (m, 2H), 2.68–2.61 (m, 2H), 2.57 (tt, *J* = 10.4, 3.9 Hz, 1H) 2.29 (t, *J* = 10.7 Hz, 1H), 2.14 (td, *J* = 10.9, 2.9 Hz, 1H), 2.00–1.90 (m, 1H), 1.79–1.70 (m, 1H), 1.59 (dtt, *J* = 13.1, 7.0, 3.7 Hz, 1H), 1.45 (qd, *J* = 11.8, 4.0 Hz, 1H), 1.24 (t, *J* = 7.1 Hz, 3H); ^13^C NMR (75 MHz, CDCl_3_) δ = 174.1, 133.3, 132.3, 128.8, 126.9, 125.8, 125.4, 93.5, 80.1, 60.4, 57.3, 55.1, 53.4, 41.9, 26.9, 24.6, 17.9, 14.2; FTIR ν = 2941, 2811, 2230, 1727, 1469, 1434, 1369, 1304, 1179, 1152, 1100, 1026, 861, 752 cm^–1^; ESI-MS *m*/*z* 364, 366 [M + H]^+^. The spectral data are in accordance with literature.^16^

#### 4.1.5 Synthesis of *tert*-Butyl 1-(4-(2-Bromophenyl)but-3-yn-1-yl)piperidine-3-carboxylate **(7b)**

Compound **7b** was synthesized according to the procedure described for compound **7a**. Starting from the alkyne **6b** (1.8 g, 7.6 mmol, 1.0 equiv) and 2-bromoiodobenzene (0.97 mL, 7.6 mmol, 1.0 equiv), product **7b** was obtained as a yellow to light brown oil (2.24 g, 5.7 mmol, 75%). *R*_f_ = 0.17 (hexane/EtOAc 4/1); ^1^H NMR (400 MHz, CDCl_3_) δ = 7.55 (dt, *J* = 8.1, 1.4 Hz, 1H), 7.41 (dt, *J* = 7.7, 1.5 Hz, 1H), 7.22 (tt, *J* = 7.7, 1.3 Hz, 1H), 7.11 (tt, *J* = 7.3, 1.5 Hz, 1H), 3.03 (d, *J* = 10.9 Hz, 1H), 2.83 (d, *J* = 11.1 Hz, 1H), 2.76–2.69 (m, 2H), 2.68–2.61 (m, 2H), 2.47 (tt, *J* = 10.4, 3.8 Hz, 1H), 2.22 (t, *J* = 10.7 Hz, 1H), 2.10 (td, *J* = 11.4, 2.9 Hz, 1H), 1.95–1.87 (m, 1H), 1.72 (dt, *J* = 13.4, 3.7 Hz, 1H), 1.63–1.50 (m, 1H), 1.43 (s, 9H), 1.37 (td, *J* = 11.9, 4.1 Hz, 1H); ^13^C NMR (101 MHz, CDCl_3_) δ = 173.7, 133.5, 132.4, 128.9, 127.0, 126.0, 125.6, 93.8, 80.3, 80.2, 57.5, 55.5, 53.6, 43.0, 28.2, 27.1, 24.8, 18.1; FTIR ν = 2975, 2939, 2810, 2232, 1720, 1470, 1434, 1365, 1242, 1132, 1101, 1026, 848, 752 cm^–1^; ESI-HRMS *m*/*z* [M + H]^+^ calcd for C_20_H_27_BrNO_2_^+^: 392.1220, found 392.1222.

#### 4.1.6 Synthesis of Ethyl 1-(4-(2′-Formyl-[1,1′-biphenyl]-2-yl)but-3-yn-1-yl)piperidine-3-carboxylate **(8a)**

Compound **8a** was synthesized according to a modified procedure developed by Wanner and co-workers.^16^ Pd_2_(dba)_3_·CHCl_3_ (70 mg, 0.068 mmol, 1 mol %), SPhos (111 mg, 0.27 mmol, 4 mol %), K_3_PO_4_ (2.88 g, 13.6 mmol, 2.0 equiv), and 2-formylphenylboronic acid (1.5 g, 10.2 mmol, 1.5 equiv) were introduced into a round-bottomed flask, after which the atmosphere was changed to N_2_. Then the aryl bromide **7a** (2.47 g, 6.78 mmol, 1.0 equiv) in degassed dioxane (6 mL) and degassed water (6 mL) were added. Afterward, the mixture was stirred at 60 °C overnight. After cooling to room temperature, DCM (60 mL) and water (60 mL) were added and the product was extracted with DCM (3 × 60 mL). The combined organic layers were washed with brine and dried over MgSO_4_, after which the solvent was removed *in vacuo* to obtain the crude product. The crude product was purified by flash column chromatography (eluting with hexane/EtOAc 95/9 to 80/20) to yield the corresponding cross-coupling product **8a** as a yellow to light brown oil (2.34 g, 6.00 mmol, 89%). *R*_f_ = 0.20 (hexane/EtOAc 3/1); ^1^H NMR (300 MHz, CDCl_3_) δ = 9.83 (s, 1H), 8.02 (dd, *J* = 7.9, 1.1 Hz, 1H), 7.63 (td, *J* = 7.5, 1.2 Hz, 1H), 7.53–7.46 (m, 2H), 7.40–7.30 (m, 4H), 4.11 (q, 7.1 Hz, 2H), 2.86 (d, *J* = 10.5 Hz, 1H), 2.64 (d, *J* = 11.4 Hz, 1H), 2.54–2.42 (m, 1H), 2.40–2.29 (m, 4H), 2.13 (t, *J* = 10.5 Hz, 1H), 2.00–1.85 (m, 2H), 1.66 (dt, *J* = 13.0, 3.7 Hz, 1H), 1.58–1.31 (m, 2H), 1.23 (t, *J* = 7.1 Hz, 3H); ^13^C NMR (75 MHz, CDCl_3_) δ = 192.0, 174.2, 144.6, 140.3, 134.2, 133.5, 132.1, 131.2, 130.2, 128.2, 128.1, 128.0, 126.8, 124.3, 93.5, 80.3, 60.5, 56.8, 55.0, 53.2, 41.9, 26.9, 24.6, 17.5, 14.3; FTIR ν = 2941, 2229, 1726, 1694, 1595, 1470, 1441, 1393, 1369, 1302, 1180, 1152, 1099, 1030, 827, 758, 644 cm^–1^; ESI-HRMS *m*/*z* [M + H]^+^ calcd for C_25_H_28_NO_3_^+^: 390.2064, found 390.2066.

#### 4.1.7 Synthesis of *tert*-Butyl 1-(4-(2′-Formyl-[1,1′-biphenyl]-2-yl)but-3-yn-1-yl)piperidine-3-carboxylate **(8b)**

Compound **8b** was synthesized according to the procedure described for compound **8a**. Starting from the aryl bromide **7b** (2.23 g, 5.7 mmol, 1.0 equiv) and 2-formylphenylboronic acid (1.3 g, 8.5 mmol, 1.5 equiv), product **8b** was obtained as a yellow to light brown oil (1.95 g, 4.7 mmol, 82%). *R*_f_ = 0.25 (hexane/EtOAc 4/1); ^1^H NMR (400 MHz, CDCl_3_) δ = 9.83 (d, *J* = 0.8 Hz, 1H), 8.02 (ddd, *J* = 7.8, 1.4, 0.5 Hz, 1H), 7.63 (td, *J* = 7.5, 1.4 Hz, 1H), 7.54–7.44 (m, 2H), 7.40–7.30 (m, 4H), 2.83 (d, *J* = 9.4 Hz, 1H), 2.66–2.59 (m, 1H), 2.39 (dt, *J* = 10.3, 3.6 Hz, 1H), 2.34 (d, *J* = 5.4 Hz, 4H), 2.06 (t, *J* = 10.7 Hz, 1H), 1.93–1.84 (m, 2H), 1.64 (dp, *J* = 10.3, 3.5 Hz, 1H), 1.51–1.44 (m, 1H), 1.42 (s, 9H), 1.32 (qd, *J* = 12.1, 4.0 Hz, 1H); ^13^C NMR (101 MHz, CDCl_3_) δ = 192.0, 173.6, 144.6, 140.2, 134.2, 133.5, 132.1, 131.2, 130.2, 128.2, 128.1, 127.9, 126.8, 124.3, 93.5, 80.3, 80.2, 56.9, 55.2, 53.2, 42.8, 28.2, 27.0, 24.7, 17.6; FTIR ν = 3061, 2975, 2937, 2810, 2228, 1720, 1694, 1597, 1469, 1440, 1391, 1365, 1194, 1147, 1101, 848, 826, 757, 644 cm^–1^; ESI-HRMS *m*/*z* [M + H]^+^ calcd for C_27_H_32_NO_3_^+^: 418.2377, found 418.2378.

#### 4.1.8 Synthesis of Ethyl 1-(4-(2′-(Hydroxylmethyl)-[1,1′-biphenyl]-2-yl)but-3-yn-1-yl)piperidine-3-carboxylate **(9a)**

To an ice-cold solution of aldehyde **8a** (2.23 g, 5.7 mmol, 1.0 equiv) in ethanol (6 mL), NaBH_4_ (0.65 g, 17.2 mmol, 2.0 equiv) was carefully added in portions. The mixture was stirred in an ice bath until completion (2 h, monitored by LC-MS and TLC hexane/EtOAc 1/1). The reaction mixture was quenched by the addition of an aqueous saturated NH_4_Cl solution (6 mL), after which the volatiles were removed *in vacuo*. Then, the product was extracted with diethyl ether (3 × 20 mL). The combined organic layers were washed with brine and dried over MgSO_4_, after which the solvent was removed *in vacuo* to obtain the reduced product **9a** as a yellow to light brown oil (2.05 g, 5.2 mmol, 91%), which was used without further purification. *R*_f_ = 0.20 (hexane/EtOAc 1/1); ^1^H NMR (400 MHz, CDCl_3_) δ 7.64 (dd, *J* = 11.6, 7.5 Hz, 1H), 7.46 (d, *J* = 7.3 Hz, 1H), 7.39 (t, *J* = 7.2 Hz, 1H), 7.35–7.27 (m, 3H), 7.20 (d, *J* = 7.6 Hz, 1H), 7.15 (d, *J* = 7z.4 Hz, 1H), 4.57 (d, *J* = 12.7 Hz, 1H), 4.37 (d, *J* = 12.9 Hz, 1H), 4.13 (2 × q, *J* = 7.0 Hz, 2H), 3.08–2.60 (m, 3H), 2.41 (dt, *J* = 20.3, 6.3 Hz, 2H), 2.35–2.23 (m, 2H), 2.03–1.92 (m, 2H), 1.86–1.74 (m, 1H), 1.71–1.63 (m, 2H), 1.34 (dd, *J* = 11.9, 5.0 Hz, 1H), 1.26 (2 × t, *J* = 7.0 Hz, 3H); ^13^C NMR (101 MHz,CDCl_3_) δ 174.2, 143.5, 139.9, 139.8, 131.9, 131.8, 129.7, 129.7, 128.0, 128.0, 127.9, 127.3, 127.2, 123.1, 91.8, 80.6, 62.8, 62.7, 60.5, 57.2, 57.1, 55.4, 54.7, 53.6, 53.0, 41.1, 41.1, 27.1, 27.0, 24.0, 17.2, 14.4, 14.3; FTIR ν = 3057, 2941, 2812, 2229, 1728, 1470, 1439, 1369, 1306, 1182, 1152, 1132, 1099, 1032, 947, 862, 758, 623, 600 cm^–1^; ESI-HRMS *m*/*z* [M + H]^+^ calcd for C_25_H_30_NO_3_^+^: 392.2220, found 392.2222. Several signals in both ^1^H and ^13^C spectra are split due to intramolecular interactions. As further proof, a NOESY spectrum is shown in the Supporting Information, in which the same phase signals for the CH_2_OH and the ring NCH_2_ protons indicate chemical exchange.^53^

#### 4.1.9 Synthesis of *tert*-Butyl 1-(4-(2′-(Hydroxylmethyl)-[1,1′-biphenyl]-2-yl)but-3-yn-1-yl)piperidine-3-carboxylate **(9b)**

Compound **9b** was synthesized according to the procedure described for compound **9a**, using *tert*-butanol as solvent instead of ethanol. Starting from aldehyde **8b** (1.95 g, 4.7 mmol, 1.0 equiv) the reduced product **9b** was obtained as a yellow to light brown oil (1.71 g, 4.1 mmol, 87%), which was used without further purification. Traces of *tert*-butanol were azeotropically removed with hexane.^54^*R*_f_ = 0.19 (hexane/EtOAc 1/1); ^1^H NMR (400 MHz, CDCl_3_) δ = 7.64 (dd, *J* = 14.0, 7.6 Hz, 1H), 7.46 (d, *J* = 7.1 Hz, 1H), 7.39 (t, *J* = 7.6 Hz, 1H), 7.35–7.27 (m, 3H), 7.20 (dd, *J* = 7.3, 1.4 Hz, 1H), 7.14 (dd, *J* = 7.5, 1.3 Hz, 1H), 4.58 (d, *J* = 12.5 Hz, 1H), 4.37 (d, *J* = 12.9 Hz, 1H), 3.03–2.48 (m, 3H), 2.42 (t, *J* = 7.8 Hz, 1H), 2.35 (t, *J* = 5.9 Hz, 1H), 2.32–2.19 (m, 2H), 2.01–1.84 (m, 2H), 1.83–1.69 (m, 1H), 1.69–1.57 (m, 2H), 1.46–1.42 (2 × s, 9H), 1.33–1.23 (m, 1H); ^13^C NMR (101 MHz, CDCl_3_) δ = 173.8, 143.5, 140.0, 139.8, 131.8, 129.7, 129.6, 128.0, 127.9, 127.9, 127.3, 127.2, 123.2, 91.9, 80.5, 80.3, 62.7, 57.3, 55.7, 54.9, 53.8, 53.1, 42.0, 28.2, 28.2, 27.3, 27.1, 24.1, 17.3; FTIR ν = 3060, 2937, 2812, 1722, 1472, 1440, 1391, 1367, 1311, 1147, 1006, 848, 756. ESI-HRMS *m*/*z* [M + H]^+^ calcd for C_27_H_34_NO_3_^+^: 420.2533, found 420.2521. Several signals in both ^1^H and ^13^C spectra are split due to intramolecular interactions. As further proof, a NOESY spectrum is shown in the Supporting Information, in which same phase signals for the CH_2_OH and the ring NCH_2_ protons indicate chemical exchange.^53^

#### 4.1.10 Synthesis of Ethyl 1-(4-(2′-(Bromomethyl)-[1,1′-biphenyl]-2-yl)but-3-yn-1-yl)piperidine-3-carboxylate **(10a)**

Alcohol **9a** (100 mg, 0.26 mmol, 1.0 equiv), CBr_4_ (110 mg, 0.33 mmol, 1.3 equiv), and polymer-supported PPh_3_ (100–200 mesh, 1.6 mmol/g) (410 mg, 0.66 mmol, 2.6 equiv) were introduced into a dried Schlenk tube and the atmosphere was changed to N_2_. Anhydrous DCM (3 mL) was then added, and the reaction mixture was stirred for 3 h at rt. Afterward, the reaction mixture was filtered and the residue was washed with DCM. Celite was added to the combined organic layers, and the crude product was dry-loaded on the Celite by evaporation of the solvent *in vacuo*. The crude product was then purified by flash column chromatography (eluting with hexane/EtOAc 95/5 to 70/30) to yield brominated product **10a** as a colorless oil (32.7 mg, 0.07 mmol, 28%). *R*_f_ = 0.40 (hexane/EtOAc 1/1); ^1^H NMR (400 MHz, CDCl_3_) δ 7.56 (d, *J* = 7.5 Hz, 1H), 7.53–7.48 (m, 1H), 7.43–7.33 (m, 4H), 7.32–7.28 (m, 1H), 7.25–7.19 (m, 1H), 4.42 (d, *J* = 10.3 Hz, 1H), 4.30 (dd, *J* = 10.3, 7.4 Hz, 1H), 4.14 (q, *J* = 7.1 Hz, 2H), 3.54 (s, 1H), 3.41–3.28 (m, 1H), 3.04–2.77 (m, 4H), 2.61–2.40 (m, 1H), 2.32–2.18 (m, 2H), 1.83 (t, *J* = 15.8 Hz, 1H), 1.72–1.57 (m, 2H), 1.47–1.33 (m, 1H), 1.24 (t, *J* = 7.2 Hz, 3H); ^13^C NMR (75 MHz, CDCl_3_) δ 174.1, 142.4, 140.9, 135.8, 131.9, 130.5, 130.2, 129.6, 128.1, 128.1, 127.6, 127.5, 123.3, 92.2, 80.2, 60.3, 57.1, 54.9, 53.1, 41.8, 32.2, 26.8, 24.5, 17.5, 14.2; FTIR ν = 2963, 2512, 1726, 1473, 1448, 1374, 1306, 1262, 1209, 1146, 1024, 860, 759, 670 cm^–1^; ESI-HRMS *m*/*z* [M + H]^+^ calcd for C_25_H_29_BrNO_2_^+^: 454.1376, found 454.1374.

#### 4.1.11 Synthesis of *tert*-Butyl 1-(4-(2′-(Bromomethyl)-[1,1′-biphenyl]-2-yl)but-3-yn-1-yl)piperidine-3-carboxylate **(10b)**

Compound **10b** was synthesized according to the procedure described for compound **10a**. Starting from the alcohol **9b** (100 mg, 0.24 mmol, 1.0 equiv) the brominated product **10b** was obtained as a colorless oil (14 mg, 0.03 mmol, 12%). Alternatively, in the case of full conversion (verified by LC-MS) the bromoform impurity could be removed by washing the Celite with hexane, after which the product (76 mg, 0.16 mmol, 66%) was recovered using EtOAc. *R*_f_ = 0.66 (hexane/EtOAc 1/1); ^1^H NMR (400 MHz, CDCl_3_) δ = 7.52 (d, *J* = 7.4 Hz, 1H), 7.48 (dd, *J* = 7.1, 1.3 Hz, 1H), 7.39–7.29 (m, 5H), 7.20 (dd, *J* = 7.3, 1.7 Hz, 1H), 4.47 (dd, *J* = 10.3, 2.9 Hz, 1H), 4.34 (d, *J* = 10.3 Hz, 1H), 2.84 (d, *J* = 11.2 Hz, 1H), 2.63 (d, *J* = 11.1 Hz, 1H), 2.43–2.28 (m, 5H), 2.05 (t, *J* = 11.2 Hz, 1H), 1.95–1.85 (m, 2H), 1.70–1.66 (m, 1H), 1.51–1.46 (m, 1H), 1.43 (s, 9H), 1.35–1.31 (m, 1H); ^13^C NMR (101 MHz, CDCl_3_) δ = 173.7, 142.5, 141.1, 135.9, 132.1, 130.7, 130.4, 129.7, 128.2, 128.2, 127.7, 127.7, 123.5, 92.4, 80.4, 80.3, 57.3, 55.3, 53.3, 42.8, 32.3, 28.2, 27.1, 24.7, 17.6; FTIR ν = 2965, 2930, 2529, 1717, 1440, 1368, 1259, 1157, 1145, 844, 756, 733, 648 cm^–1^; ESI-HRMS *m*/*z* [M + H]^+^ calcd for C_27_H_33_NO_2_^+^: 482.1689, found 482.1690.

#### 4.1.12 Synthesis of Ethyl 1-(4-(2′-(Fluoromethyl)-[1,1′-biphenyl]-2-yl)but-3-yn-1-yl)piperidine-3-carboxylate **(11a)**

Alcohol **9a** (100 mg, 0.26 mmol, 1.0 equiv) was introduced into an oven-dried Schlenk tube and the atmosphere was changed to N_2_. Anhydrous DCM (3 mL) was then added, and the solution was cooled in an ice bath. Afterward, DAST (70 μL, 0.51 mmol, 2.0 equiv) was added, and the reaction mixture was stirred at 0 °C for 3 h. The reaction was quenched by the addition of ice, and the mixture was stirred until the ice melted. Then, the product was extracted with DCM. The combined organic layers were washed with brine and dried over MgSO_4_, after which the solvent was removed *in vacuo* to obtain the crude product. The crude product was purified by flash column chromatography (eluting with DCM/EtOAc 97/3 to 70/30) to yield the fluorinated product **11a** as a yellow oil (16.8 mg, 0.04 mmol, 17%). *R*_f_ = 0.20 (DCM/EtOAc 4/1); ^1^H NMR (400 MHz, CDCl_3_) δ 7.55 (d, *J* = 6.9 Hz, 1H), 7.46 (td, *J* = 7.6, 2.2 Hz, 1H), 7.44–7.35 (m, 2H), 7.32 (td, *J* = 6.6, 1.7 Hz, 2H), 7.29–7.22 (m, 2H), 5.42–5.08 (m, 2H), 4.12 (q, *J* = 7.1 Hz, 2H), 2.92 (d, *J* = 8.8 Hz, 1H), 2.73–2.66 (m, 1H), 2.58–2.49 (m, 1H), 2.45–2.33 (m, 4H), 2.15 (t, *J* = 10.4 Hz, 1H), 2.00–1.91 (m, 2H), 1.68 (dt, *J* = 13.2, 3.6 Hz, 1H), 1.62–1.50 (m, 1H), 1.39 (qd, *J* = 11.9, 4.1 Hz, 1H), 1.24 (t, *J* = 7.1 Hz, 3H); ^13^C NMR (101 MHz, CDCl_3_) δ 174.2, 142.4, 140.2 (d, *J* = 4.9 Hz), 134.6 (d, *J* = 16.5 Hz), 132.1, 130.3, 129.8, 128.3 (d, *J* = 3.0 Hz), 128.0, 127.9, 127.7, 127.6, 123.4, 92.2, 82.7 (d, *J* = 164.9 Hz), 80.2, 60.7, 57.2, 55.0, 53.3, 41.9, 27.0, 24.7, 17.6, 14.3; ^19^F NMR (376 MHz, CDCl_3_) δ −207.65 (t, *J* = 47.9 Hz); FTIR ν = 2942, 1726, 1470, 1441, 1370, 1304, 1262, 1180, 1152, 1134, 1099, 1030, 758, 735, 675 cm^–1^; ESI-HRMS *m*/*z* [M + H]^+^ calcd for C_25_H_29_FNO_2_^+^: 394.2177, found 394.2171.

#### 4.1.13 Synthesis of *tert*-Butyl 1-(4-(2′-(Fluoromethyl)-[1,1′-biphenyl]-2-yl)but-3-yn-1-yl)piperidine-3-carboxylate **(11b)**

Compound **11b** was synthesized according to the procedure described for compound **11a** with the addition of dried (100 °C, vacuum, on.) CsF (73 mg, 0.48 mmol, 2.0 equiv) to the reaction mixture. Starting from the alcohol **9b** (100 mg, 0.24 mmol, 1.0 equiv), the fluorinated product **11b** was obtained as a yellow oil (19 mg, 0.05 mmol, 19%). *R*_f_ = 0.25 (DCM/EtOAc 9/1); ^1^H NMR (400 MHz, CDCl_3_) δ 7.55 (d, *J* = 6.9 Hz, 1H), 7.49–7.43 (m, 1H), 7.42–7.35 (m, 2H), 7.32 (ddd, *J* = 6.7, 5.8, 1.7 Hz, 2H), 7.29–7.23 (m, 2H), 5.44–5.05 (m, 2H), 2.85 (d, *J* = 10.8 Hz, 1H), 2.64 (d, *J* = 11.3 Hz, 1H), 2.44–2.37 (m, 1H), 2.34 (s, 4H), 2.06 (t, *J* = 10.6 Hz, 1H), 1.94–1.85 (m, 2H), 1.66 (dt, *J* = 13.1, 3.4 Hz, 1H), 1.43 (s, 9H), 1.38–1.25 (m, 2H); ^13^C NMR (101 MHz, CDCl_3_) δ 173.7, 142.4, 140.2 (d, *J* = 4.8 Hz), 134.6 (d, *J* = 16.6 Hz), 132.1, 130.3, 129.8, 128.3 (d, *J* = 2.9 Hz), 128.0, 127.9, 127.7, 127.6, 123.4, 92.3, 82.8 (d, *J* = 164.7 Hz), 80.3, 80.2, 57.3, 55.3, 53.3, 42.8, 28.2, 27.1, 24.7, 17.6; ^19^F NMR (376 MHz, CDCl_3_) δ −207.56 (td, *J* = 47.8, 7.4 Hz); FTIR ν = 2970, 2942, 2227, 1722, 1450, 1440, 1367, 1217, 1147, 1101, 848, 757, 735 cm^–1^; ESI-HRMS *m*/*z* [M + H]^+^ calcd for C_27_H_33_FNO_2_^+^: 422.2490, found 422.2483.

### 4.2 Radiochemistry

#### 4.2.1 General Considerations

Reagents were obtained from commercial suppliers and used as received: 0.9% sodium chloride was purchased from B. Braun; ethanol, anhydrous acetonitrile, K_2_CO_3_, and Kryptofix 222 were purchased from Merck; HPLC-grade acetonitrile was purchased from ITW Reagents; HPLC-grade water was purchased from VWR; QMA-light and C18-light Sep-Paks were purchased from Waters Corporation. QMA-light Sep-Paks were flushed with 10 mL of 0.04 M K_2_CO_3_ and 10 mL of water prior to use. C18 Sep-Paks were flushed with 5 mL of acetonitrile followed by 10 mL of water prior to use.

Analytical HPLC was performed by using a Knauer Smartline system equipped with a Gabi Star radiation detector and a UV detector. Quality control samples were measured using an Intertsil ODS-3 column (5 μm, 4.6 mm × 250 mm). A gradient elution was performed using water (+0.1% TFA) and acetonitrile. For the first three minutes 100% water (+0.1% TFA) was used as the eluent, after which the percentage of acetonitrile was increased to 25% over 7 min and then to 100% over 15 min. Then 100% acetonitrile was used for 1 min, after which the first mixture was employed for the remaining 4 min. The flow rate was 1.0 mL/min for the entire elution program. Meanwhile, semipreparative HPLC was performed using a Kinetex 5 μM 100A C18 column (150 mm × 10 mm). A gradient elution was performed using water (+0.1% TFA) to which was added 5 mL of 0.9% NaCl per liter and acetonitrile. For the first three minutes 30% acetonitrile was used as eluent, after which the percentage of acetonitrile was increased to 100% over 20 min and kept for 4 min. Afterward, the elution was switched back to the first mixture for the remaining 3 min. The flow rate was 2.0 mL/min for the entire elution program.

#### 4.2.2 Synthesis of [^18^F]-1-(4-(2′-(Fluoromethyl)-[1,1,-biphenyl]-2-yl)but-3-yn-1-yl)piperidine-3-carboxylic Acid **([**^**18**^**F]4)** through Ethyl Ester Precursor **10a**

Aqueous [^18^F]fluoride was purchased from Radboud Translational Medicine B.V. (Nijmegen, The Netherlands). 1.5 GBq of [^18^F]fluoride was trapped on a QMA-light SepPak and then eluted into a reaction vial using a mixture of 700 μL 0.095 M Kryptofix 222 in acetonitrile and 360 μL 0.04 M K_2_CO_3_ in water. The resulting solution was dried azeotropically under an argon flow at 105 °C by the addition of anhydrous acetonitrile (3 × 1 mL). Afterward, brominated precursor **10a** was added (0.5 mg dissolved in 1 mL of anhydrous acetonitrile) and the reaction mixture was heated at 105 °C for 15 min to give crude **[**^**18**^**F]11a** (3% conversion as confirmed by HPLC analysis). Afterward, the reactor was cooled to 100 °C, 200 μL of 0.01 M NaOH was added, and the hydrolysis reaction was heated for 20 min. HPLC analysis indicated complete degradation of the product.

#### 4.2.3 Synthesis of [^18^F]-1-(4-(2′-(Fluoromethyl)-[1,1,-biphenyl]-2-yl)but-3-yn-1-yl)piperidine-3-carboxylic Acid **([**^**18**^**F]4)** through *tert*-Butyl Ester Precursor **10b**

Crude **[**^**18**^**F]11b** was synthesized according to the procedure described for **[**^**18**^**F]11a** starting from [^18^F]fluoride (1.5 GBq) and brominated precursor **10b** (0.5 mg dissolved in 1 mL of anhydrous acetonitrile). After HPLC analysis to confirm the formation of **[**^**18**^**F]11b** (55% conversion), aliquots of the reaction mixture were added to reaction vials containing acidic media to attempt hydrolysis of the *tert*-butyl ester:

- crude [^18^F]-11b was added to 150 μL 2 M aq. HCl and stirred at 105 °C for 10 min.
- crude [^18^F]-11b was added to 250 μL formic acid and stirred at 50 °C for 15 min.
- crude [^18^F]-11b was added to 150 μL TFA and stirred at rt for 10 min.
- crude [^18^F]-11b was added to 150 μL TFA and stirred at 105 °C for 20 min.

Unfortunately, none of the deprotection conditions led to clean deprotection of the *tert*-butyl ester, with mostly starting material **[**^**18**^**F]11b** or lipophilic degradation products detected by HPLC analysis.

#### 4.2.4 Synthesis of [^18^F]-Ethyl 1-(4-(2′-(Fluoromethyl)-[1,1′-biphenyl]-2-yl)but-3-yn-1-yl)piperidine-3-carboxylate **([**^**18**^**F]11a)**

Crude **[**^**18**^**F]11a** was synthesized according to the procedure described above starting from [^18^F]fluoride (1.5 GBq) and brominated precursor **10a** (2.1 mg dissolved in 1 mL of anhydrous acetonitrile). The reaction mixture was purified by semipreparative HPLC, during which the product peak (retention time 14.3 min) was collected and diluted with water (20 mL). The resulting solution was passed through a C18 SepPak filter to remove solvent while trapping **[**^**18**^**F]11a**. The product was then eluted with 0.5 mL EtOH from the male side of the SepPak. Afterward, EtOH was concentrated to a volume of 50 μL, and 800 μL of 0.9% NaCl was added to give the final formulated dose of **[**^**18**^**F]11a** (2.9 MBq, 0.52% RCY (radiochemical yield, decay-corrected)).

### 4.3 PET Imaging

#### 4.3.1 General Considerations

All animal experiments were approved by the relevant German authority (Landesamt für Natur, Umwelt, undVerbraucherschutz Nordrhein-Westfalen) for compliance with the Animal Protection Act in conjunction with the regulation for the protection of animals used for experimental and other scientific purposes (Project-file number 81–02.04.2021.A430). Male Wistar rats at 12 weeks of age were purchased from Janvier Laboratories and housed under a 12-h-light/12-h-dark cycle with free access to food and water. The room temperature and relative humidity were kept between 20–25 °C and 45–65%, respectively.

#### 4.3.2 Rodent Imaging

Rodent imaging was performed using a small animal PET/CT system (i.e., β-CUBE and X-CUBE from Molecubes NV, Gent, Belgium). Anesthesia was induced in a healthy male Wistar rat using a 4% isoflurane dilution in medical-grade compressed air at 0.8 L/min, and was maintained with 2–2.5% isoflurane/O_2_ throughout the imaging study. Body temperature was maintained by using a heating pad. The animal was injected *via* the lateral tail vein with the radioligand (0.85 MBq) with a total volume of 600 μL tracer.

A dynamic 45 min PET scan was initiated immediately after the tracer injection. The PET axial field of view is 160 mm. Afterward, a CT scan was initiated with the following acquisition settings: standard high-resolution protocol, 440 μA, 50 kVp, 32 ms exposure time, and 1080° rotation in a spiral mode with 960 exposures/360°; the duration of each CT scan was approximately 7 min. The CT axial field of view is 37 mm. During the scans, the isoflurane concentration was adapted between 1.5 and 2% in order to achieve a respiratory rate between 75–50 breaths per minute.

#### 4.3.3 Image Processing and Analysis

CT images were reconstructed using an iterative reconstruction algorithm (i.e., ISRA) to an isometric voxel size of 0.1 mm in a 700 × 700 × 1400 matrix. Using vendor software, CT values were converted into Hounsfield units (HU) using the formula:
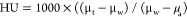
where μ_w_ is the linear attenuation coefficient of water, μ_a_ is the linear attenuation coefficient of air and μ_t_ is the linear attenuation coefficient of the tissue.

PET data were reconstructed using a three-dimensional ordered-subset expectation maximization (i.e., OSEM-3D with 30 iterations) with an energy window of ±15% from a peak of 511 keV to an isometric voxel size of 0.4 mm in a 192 × 192 × 384 matrix. CT-based corrections (i.e., attenuation correction, scatter correction, dead time, and random events) were applied during the PET reconstruction.

PET and CT images were automatically aligned after reconstruction by using the matrix generated from a capillary phantom scan. For the Image preparation, the PMOD software package version 4.4 (PMOD Technologies LLC, Zürich, Switzerland) was used.

## Supporting Information Available

Details on the optimization for the synthesis of precursors and reference standards, HPLC spectra of the radiosynthesis, and analytical spectra of synthesized compounds (PDF)

## Abbreviations

BBB: blood–brain barrier
CNS: central nervous system
GABA: γ-aminobutyric acid
GAT: GABA transporter
GAT-1: GABA transporter-1
HPLC: high-performance liquid chromatography
HU: Hounsfield units
LC-MS: liquid chromatography-mass spectrometry
PET: positron emission tomography
PS–PPh_3_: polymer-supported PPh_3_
RCP: radiochemical purity
RCY: radiochemical yield
