# Supplementary material for: Synthesis and Evaluation of Benzylic 18F-Labeled N-Biphenylalkynyl Nipecotic Acid Derivatives for PET Imaging of GABA Transporter 1
Source: ACS Chem Neurosci. 2025 Jan 29;16(4):711–22. doi: 10.1021/acschemneuro.4c00782 (PMC11843609; doi:10.1021/acschemneuro.4c00782)
Supplement: Supplementary file 1 — cn4c00782_si_001.pdf [file cn4c00782_si_001.pdf]

Supplementary information

Synthesis and evaluation of benzylic ^{18}F-labeled *N*-biphenylalkynyl nipecotic acid derivatives for PET imaging of GABA transporter 1

Niels Knippenberg*, Matthias Bauwens, Alexandru Florea, Soma Rudi, Olaf Schijns, Govert Hoogland, Vincent Ornelis, Ronny Mohren, Michiel Vandenbosch, Felix M. Mottaghy, Thomas J. Cleij, Kasper Eersels, Bart van Grinsven, Hanne Diliën

*Corresponding author: Niels Knippenberg, niels.knippenberg@maastrichtuniversity.nl

Optimization for the synthesis of precursors and reference standards	S2
Transesterification	S2
Tosylation and deoxybromination reactions using the model system biphenyl-4-methanol	S5
Synthesis and stability of brominated precursors **10a-b**	S9
Deoxyfluorination reactions using the model system biphenyl-4-methanol	S16
Synthesis of reference standards **11a-b**	S20
Radiosynthesis	S23
Ethyl ester deprotection	S23
Tert-butyl ester deprotection	S24
Ethyl ester prodrug approach	S27
Molar activity estimation	S30
Analytical spectra of synthesized compounds	S32
References	S57

Optimization for the synthesis of precursors and reference standards

Transesterification

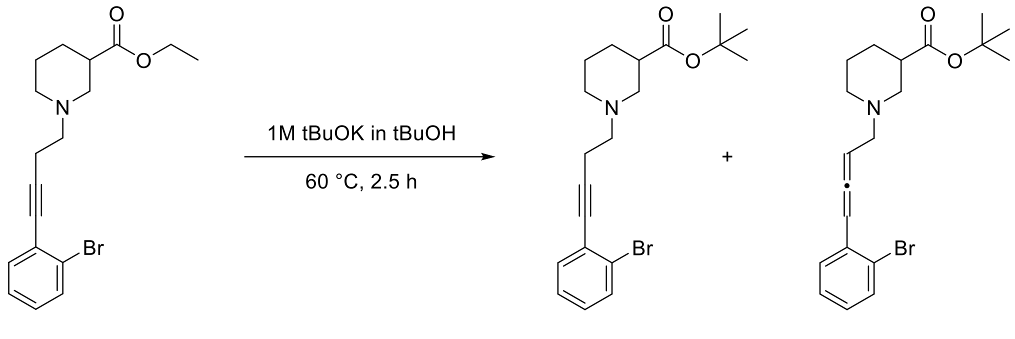

Scheme S1: Transesterification of **7a** to **7b**.

To access the *tert*-butyl precursor **7b**, it was first attempted to perform a transesterification of **7a** to **7b** using potassium *tert*-butoxide (**Scheme S1**). To that end, the ethyl ester **7a** (120 mg, 0.33 mmol, 1.0 equiv.) was added to an oven-dried Schlenk flask containing 1 M tBuOK in tBuOH (1 mL, 1 mmol, 3.0 equiv.). The reaction mixture was stirred at 60 °C for 1 h, after which conversion was monitored using LC-MS. Since, the reaction had not reached full conversion, an additional 3.0 equiv. of 1 M tBuOK in tBuOH was added and the reaction mixture was stirred at 60 °C for 1.5 h. After this time, LC-MS confirmed full conversion and water (40 mL) was added to quench the reaction. The product was extracted with DCM (3 x 30 mL). The combined organic layers were dried over $MgSO_4$ after which the solvent was removed *in vacuo* to obtain the crude product, which was analyzed with LC-MS and NMR.

While full conversion can be seen by the absence of ethyl ester signals in the NMR and the absence of starting material in the LC-MS, the transesterification did not smoothly yield the desired product. Instead, LC-MS and NMR analysis indicate the formation of the desired product and an allene side-product (**3b'**, **Scheme S1**), formed through base-catalyzed alkyne-to-allene isomerization.[1] This can be observed by the two separate LC-MS signals with the same m/z value (**Figure S1b**). Moreover, the allene moiety can be observed in the proton NMR spectrum as a signal at 5.64 and 6.62 ppm, coupling to 91.4 and 93.6 ppm in the HSQC spectrum, respectively (**Figures S2-3**). These NMR signals are in accordance with literature presenting similar nipecotic acid-based GAT1 inhibitors with an allene linker.[2] Given the difficulty to separate both alkyne and allene products, it was decided to synthesize the *tert*-butyl precursor **10b** through the same linear pathway starting as the ethyl precursor starting from *tert*-butyl nipecotate **5b**.

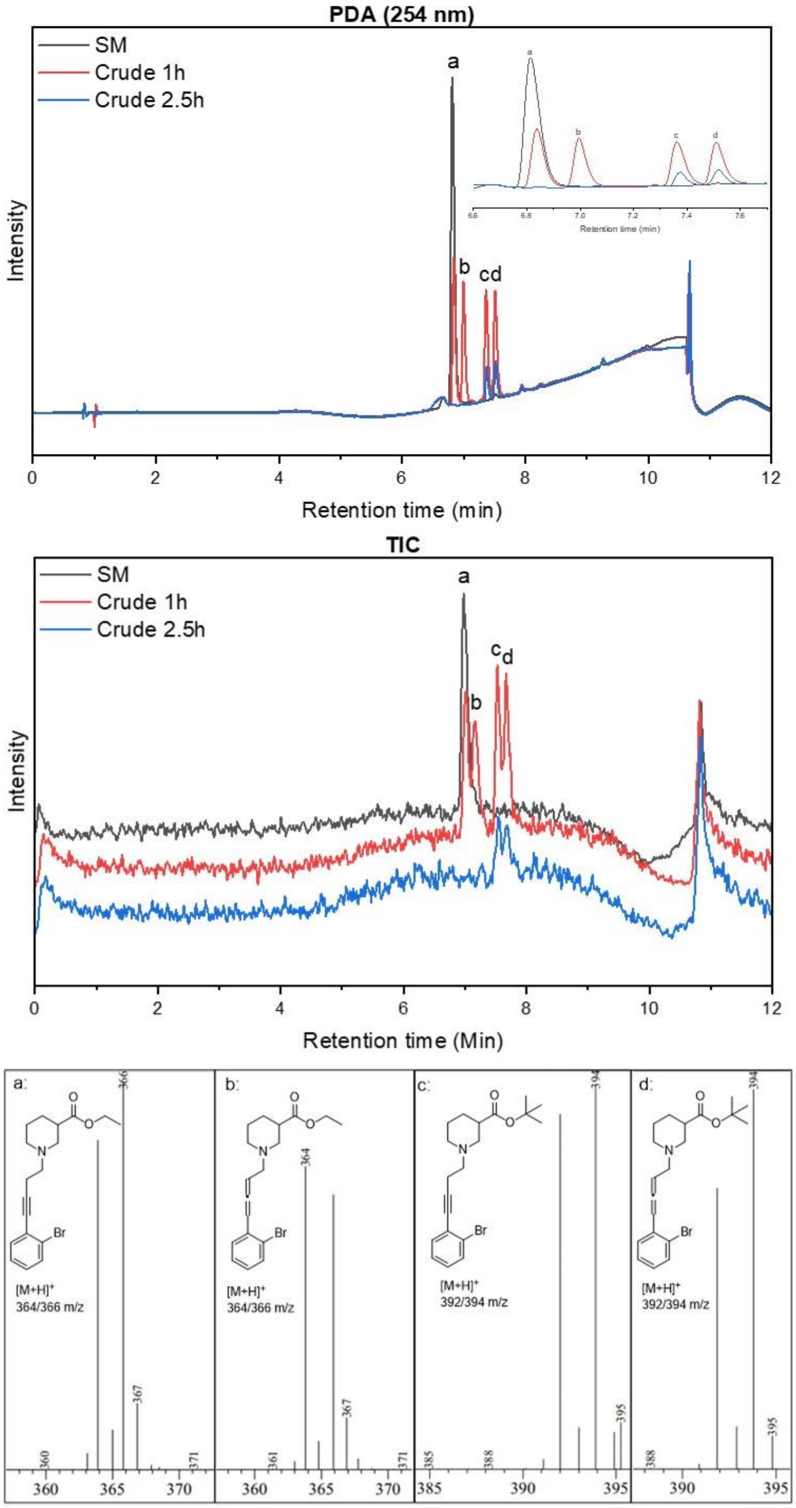

Figure S1: Crude LC-MS spectra of the transesterification reaction. Top to bottom: PDA spectra (254 nm), TIC spectra, ESI-MS spectra.

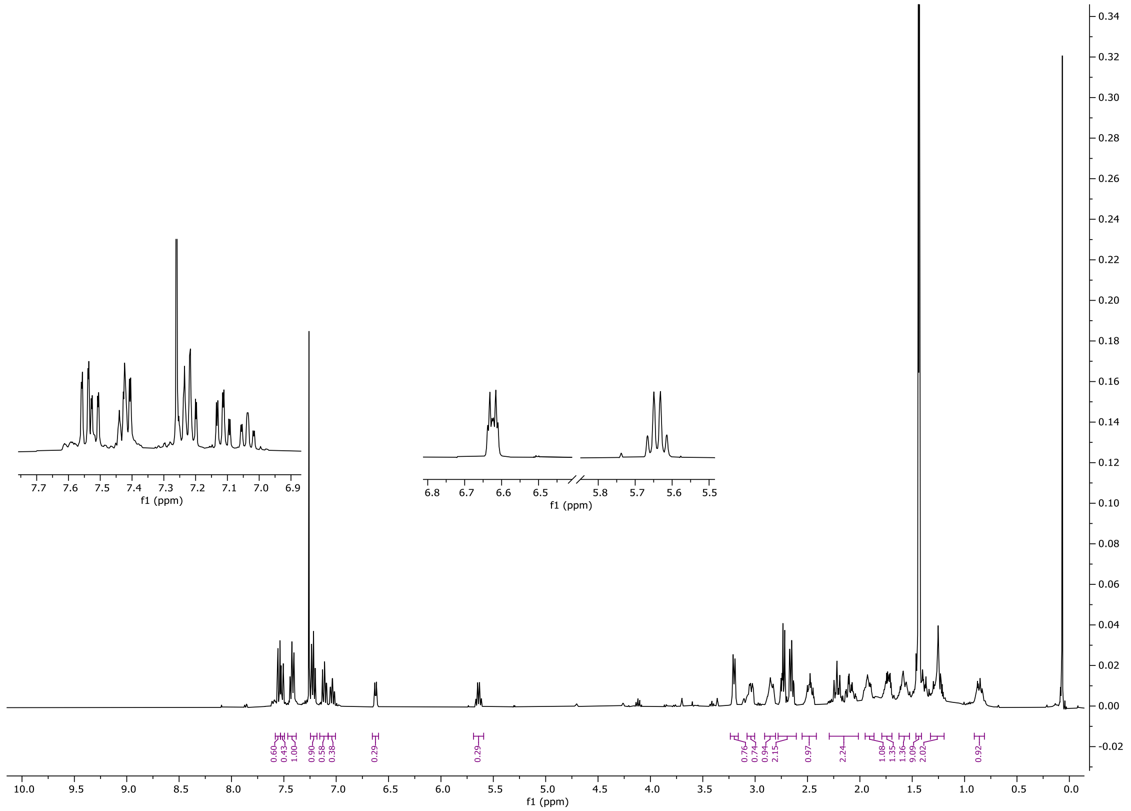

Figure S2: Crude ^{1}H NMR ($CDCl_3$, 400 MHz) of the transesterification of **7a**.

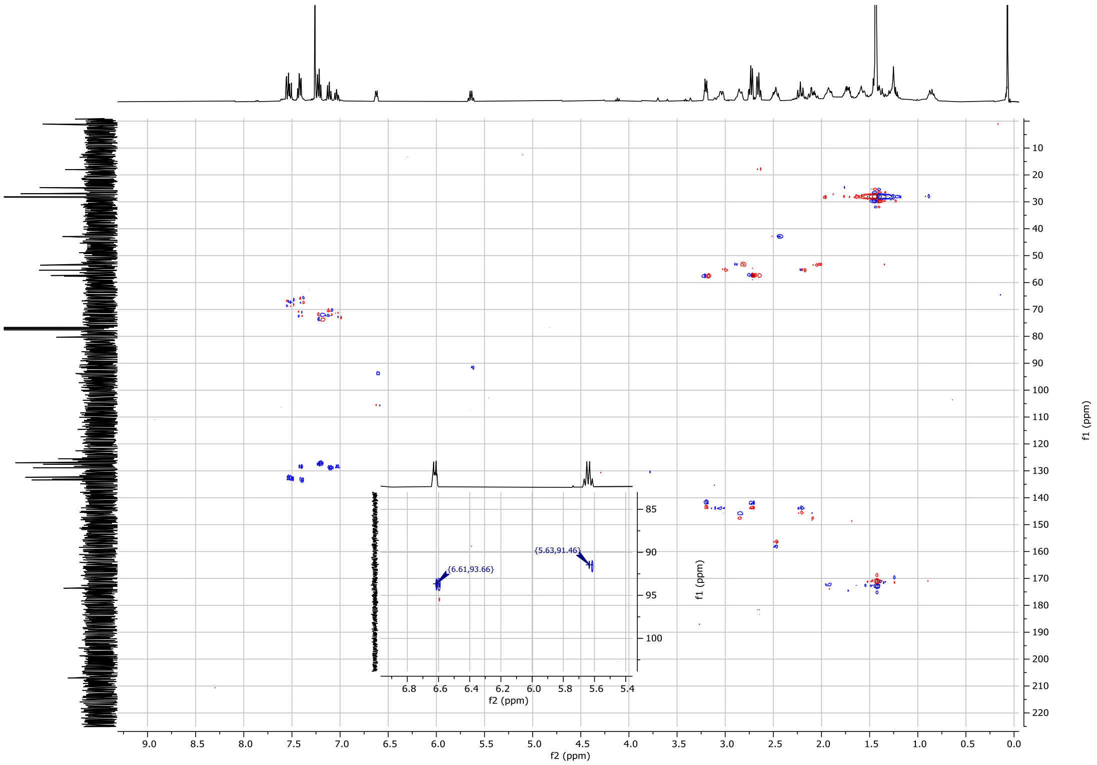

Figure S3: Crude HSQC ($CDCl_3$, 400 MHz) of the transesterification of **7a**.

Tosylation and deoxybromination reactions using the model system biphenyl-4-methanol

To convert the alcohol moiety of compound **9a-b** into a good leaving group, several reactions conditions were screened using the model system biphenyl-4-methanol (**S1**) (**Scheme S2**). In short, a tosylation reaction under classic tosylation conditions afforded the chlorinated product **S3** instead of the tosylated product **S2.** [3] Therefore, it was decided to perform an Appel bromination to install a bromine as better leaving group. This procedure was then optimized using polymer-supported PPh_3 (PS-PPh_3) to ease purification for the unstable radiolabeling precursor **9a-b**.

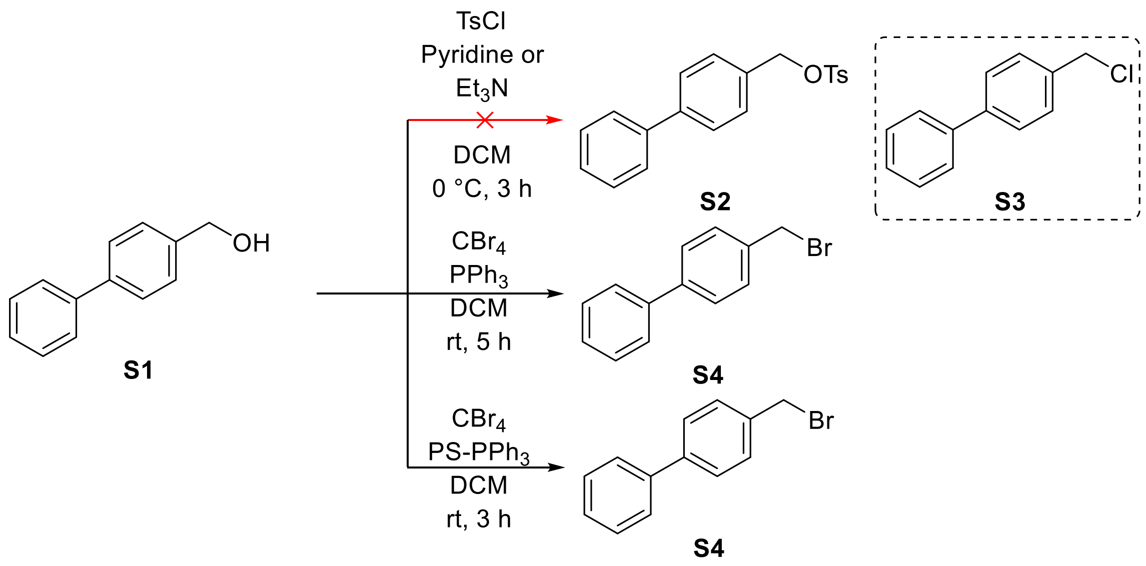

Scheme S2: Tosylation and deoxybromination test reactions using the model system biphenyl-4-methanol **S1**.

First a tosylation reaction was performed using classic tosylation conditions. To that end, TsCl (410 mg, 2.2 mmol, 2.2 equiv.) was added to a stirred solution of biphenyl-4-methanol (**S1**) (184 mg, 1.0 mmol, 1.0 equiv.) in DCM (10 mL) at 0 °C. Afterwards, pyridine (0.24 mL, 3.1 mmol, 3.1 equiv.) or Et_3N (0.43 mL, 3.1 mmol, 3.1 equiv.) was added. The reaction was stirred at 0 °C for 3 h, then diluted with diethyl ether (35 mL) and washed consecutively with water (35 mL), a saturated aqueous solution of NH_4Cl (35 mL), saturated aqueous solution of $NaHCO_3$ (35 mL), and brine (2 x 35 mL), dried over $MgSO_4$, filtered and concentrated in vacuo. The crude product was analyzed by NMR (**Figures S4-5**) and found to contain TsCl and 4-(chloromethyl)-1,1'-biphenyl (**S3**) [3], with the benzylic 4.65 and 46.1 ppm signals in ^{1}H and ^{13}C NMR in accordance with literature for 4-(chloromethyl)-1,1'-biphenyl (**S3**). [4]

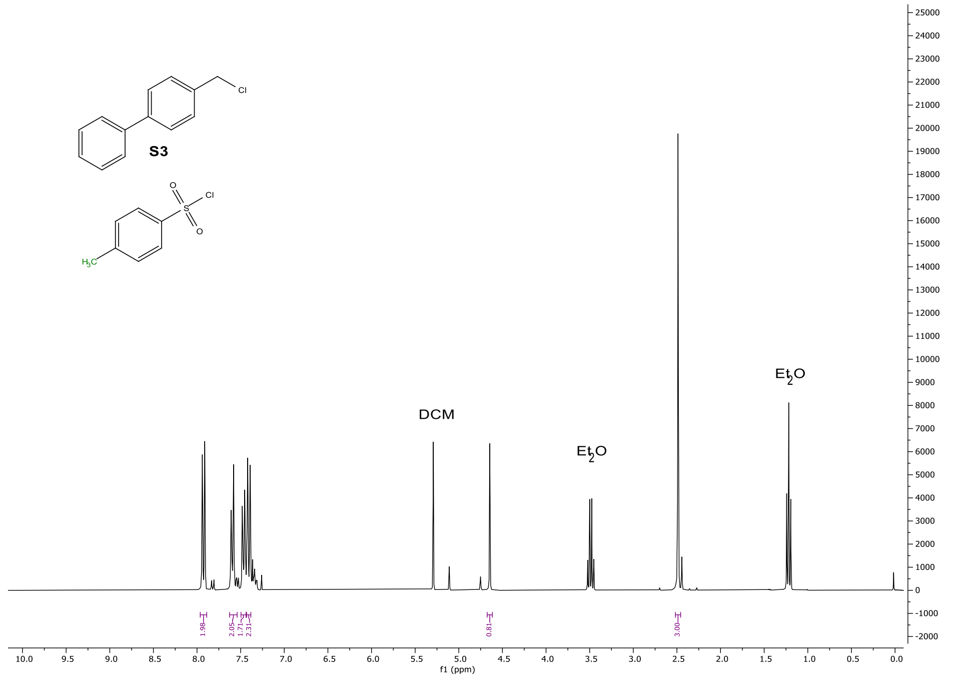

Figure S4: Crude ^{1}H NMR spectrum ($CDCl_3$, 300 MHz) of 4-(chloromethyl)-1,1'-biphenyl (**S3**).

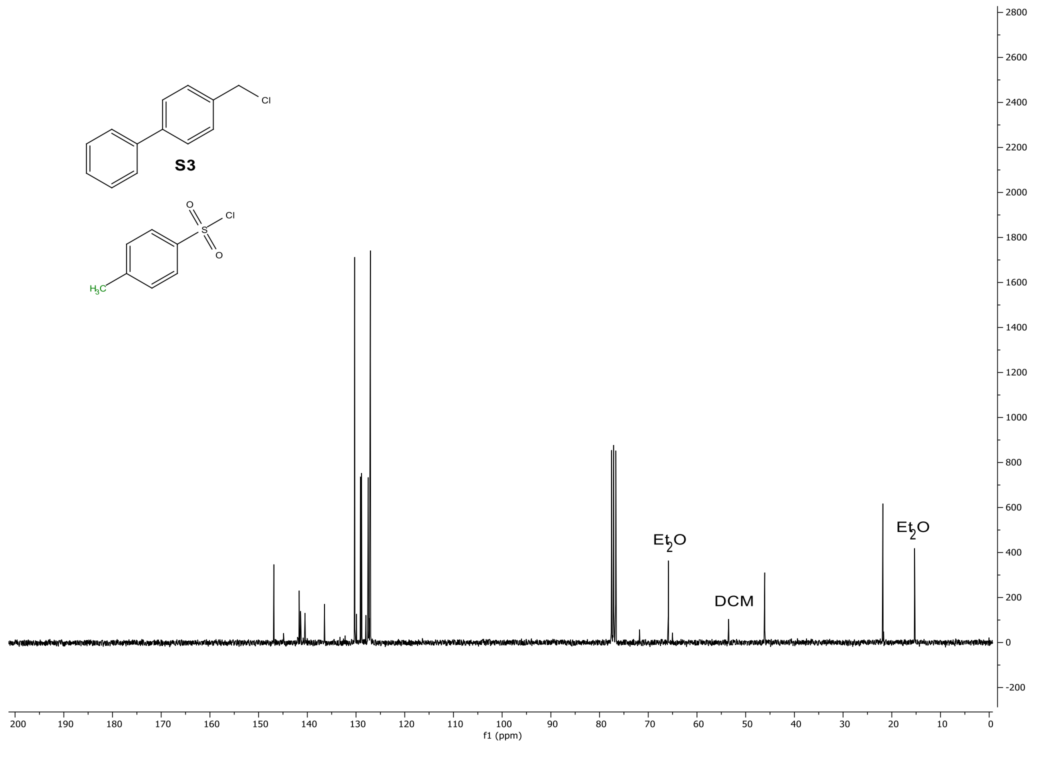

Figure S5: Crude ^{13}C NMR spectrum ($CDCl_3$, 75 MHz) of 4-(chloromethyl)-1,1'-biphenyl (**S3**).

Due to the failed tosylation, it was decided to install a bromine leaving group through an Appel reaction. To that end, CBr_4 (0.20 g, 0.60 mmol, 1.1 equiv.) and PPh_3 (0.16 g, 0.60 mmol, 1.1 equiv.) were added to a stirred solution of biphenyl-4-methanol in dry DCM under N_2 atmosphere. The reaction mixture was stirred at room temperature for 4 h after which conversion was verified by TLC (Hexane/EtOAc 3/1). Since the reaction did not go to completion, an additional 0.4 equiv. of both CBr_4 and PPh_3 were added and the reaction mixture was stirred for an additional hour. After this time, the starting material was fully consumed, and the solvent was removed *in vacuo*. The crude product was analyzed by NMR (**Figure S6**) and found to contain (oxidized) triphenylphosphine, bromoform and 4-(bromomethyl)-1,1'-biphenyl (**S4**), with the benzylic 4.55 ppm signal in ^{1}H NMR in accordance with literature for 4-(bromomethyl)-1,1'-biphenyl (**S4**).[5]

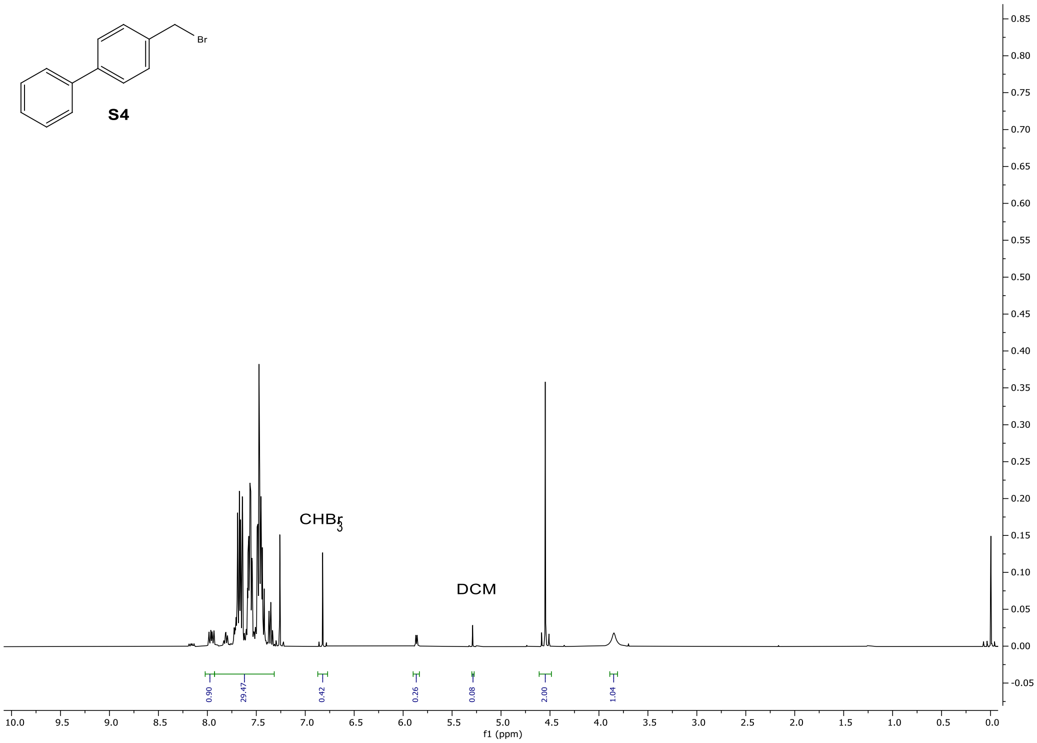

Figure S6: Crude ^{1}H NMR spectrum ($CDCl_3$, 400 MHz) of 4-(bromomethyl)-1,1'-biphenyl (**S4**).

In order to ease purification for the radiolabeling precursors **10a-b**, the Appel conditions were then further optimized by using polymer-supported PPh_3 (PS-PPh_3). The attractive feature of the PS-PPh_3 is that product isolation is facilitated by the ready separation of the reactive polymer-supported species by filtration, leaving only the product and bromoform in the filtrate. [6]

To perform the bromination, biphenyl-4-methanol (80 mg, 0.43 mmol, 1.0 equiv.), CBr_4 (0.16 g, 0.48 mmol, 1.1 equiv.), and PS-PPh_3 (100-200 mesh, 1.6 mmol/g) (0.60 g, 0.96 mmol, 2.2 equiv.) were introduced into a dried Schlenk tube and the atmosphere was exchanged to N_2. Anhydrous DCM (6 mL) was then added and the reaction mixture was stirred for for 3 hours at rt. Afterwards, the reaction mixture was filtered and the residue was washed with DCM. The combined organic layers were concentrated *in vacuo* to obtain the crude product as a white solid (105 mg, 43 mmol, quant.). NMR analysis (**Figure S7**) confirmed that the brominated product **S4** was obtained in full conversion with only a small bromoform impurity. ^1H NMR (400 MHz, $CDCl_3$) δ = 7.62 – 7.55 (m, 4H), 7.50 – 7.42 (m, 4H), 7.40 – 7.34 (m, 1H), 4.56 (s, 2H). The spectral data is in accordance with literature. [5]

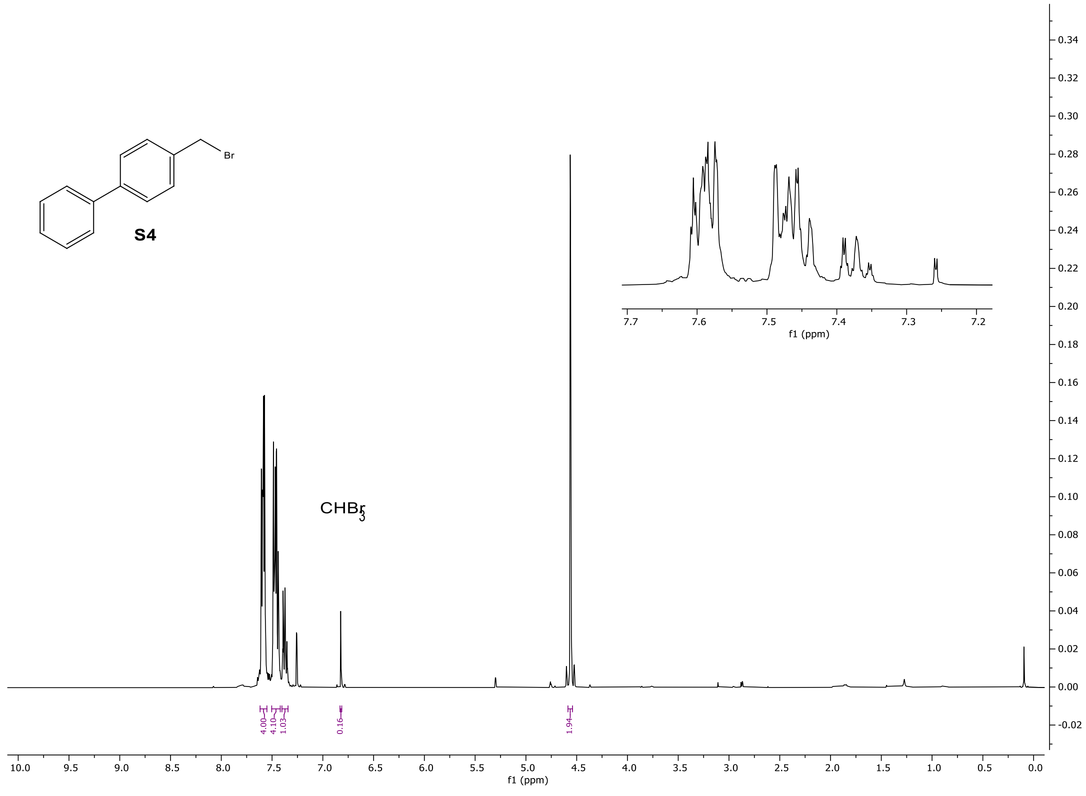

Figure S7: Crude ^1H NMR spectrum ($CDCl_3$, 400 MHz) of 4-(chloromethyl)-1,1'-biphenyl (**S4**).

Synthesis and stability of brominated precursors **10a-b**

With an optimized bromination procedure for the model system biphenyl-4-methanol in hand, this procedure was used to synthesize the brominated precursors **10a-b**. For the synthesis of the ethyl ester precursor **10a** it was found that the reaction did not go to completion within 3 hours, unlike the model system (**Figure S8**). It was assumed a bigger substrate could have more difficulty aligning properly with the activated PS-PPh_3 moieties in the heterogeneous reaction, and therefore the reaction time was increased. Unfortunately, after 5 hours the starting material was still visible in the LC-MS (peak a), but also several side products had formed (peak c in TIC, peak d in PDA) (**Figure S8**). To our delight, slightly increasing the excess of reagents (CBr_4 from 1.1 equiv. to 1.3 equiv.; PS-PPh_3 from 2.2 equiv. to 2.6 equiv.) allowed the bromination to proceed to near completion within 3 hours without the formation of side-products for both precursor **10a** and **10b** (**Figures S9-10**).

Long term storage of the brominated precursors was found to be difficult, as they degraded after storing in the freezer (-20 °C) for a month (**Figure S11**). Therefore, a survey of storage conditions was performed, showing that the compound degraded within days when stored as its pure form in the desiccator, but was relatively stable for at least a week in the freezer or preferably as MeCN solution (**Figure S12**). Proposed degradation products that were observed during these studies included the stabilized carbocation $[M\text{-}Br]^+$ (374 m/z) resulting from cleavage of the bromide and its acetonitrile adduct (415 m/z). Therefore, it seems that the cleavage of the carbon-bromide bond and the formation of the stabilized carbocation is a likely mechanism for further degradation of the precursor. In order to obtain more information on the bond cleavage, studies were undertaken in which the dissolved precursor was mixed with the radical scavenger TEMPO (0.1 equiv.). The addition of TEMPO did not significantly change the amount of degradation after 7 days (compared to precursor in MeCN without TEMPO), indicating that it is not likely for the degradation to proceed through a radical pathway. Moreover, it was found that the addition of water (10 v/v%) did not influence the degradation either, indicating no direct substitution of the benzylic bromide moiety occurred in neutral aqueous conditions. (**Figure S13**).

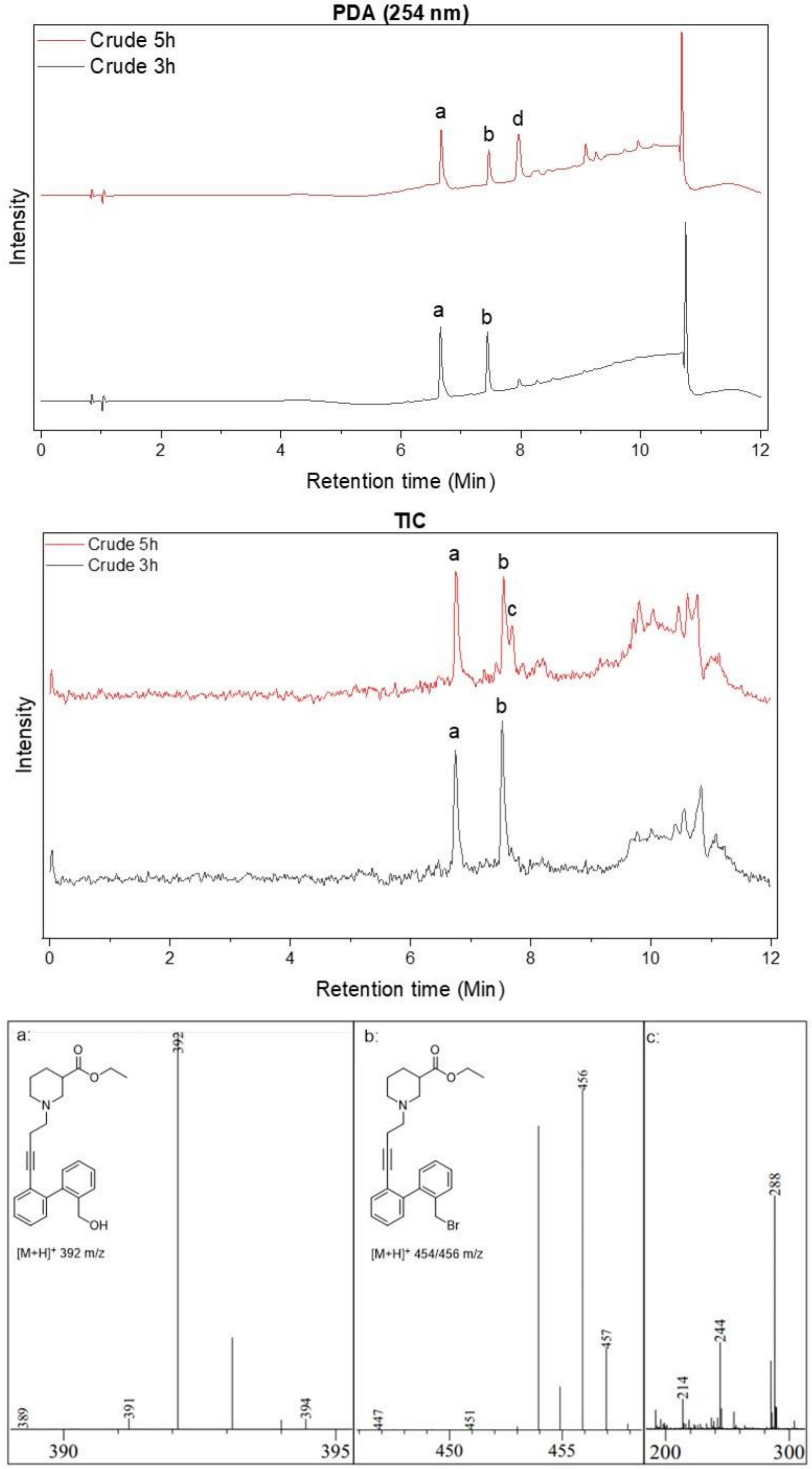

Figure S8: Crude LC-MS spectra for synthesis of compound **10a** using 1.1 equiv. CBr_4 and 2.2 equiv. PS-PPh_3. Top to bottom: PDA spectra (254 nm), TIC spectra, ESI-MS spectra.

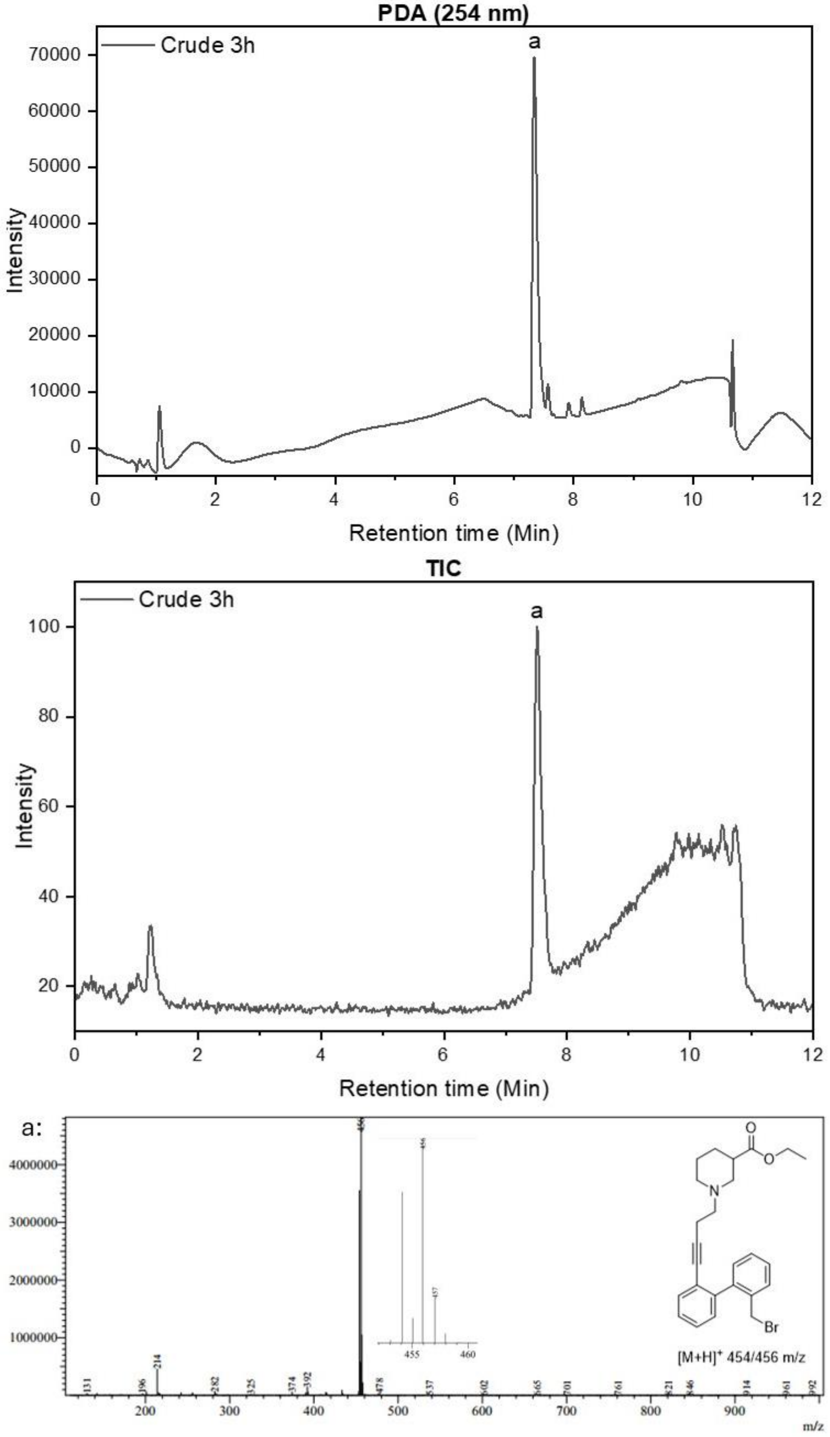

Figure S9: Crude LC-MS spectra for synthesis of compound **10a** using 1.3 equiv. CBr_4 and 2.6 equiv. PS-PPh_3. Top to bottom: PDA spectra (254 nm), TIC spectra, ESI-MS spectra.

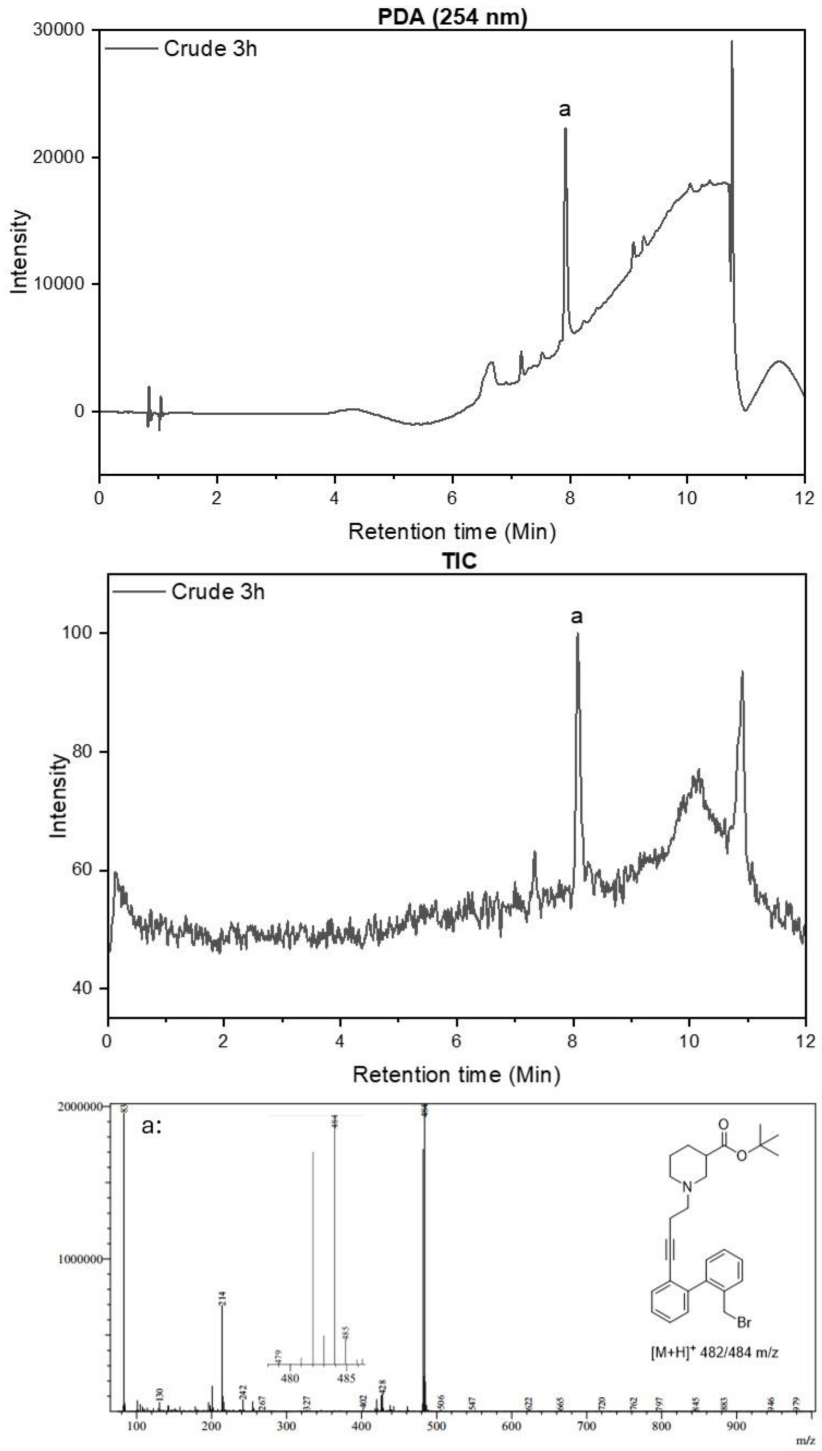

Figure S10: Crude LC-MS spectra for synthesis of compound **10b** using 1.3 equiv. CBr_4 and 2.6 equiv. PS-PPh_3. Top to bottom: PDA spectra (254 nm), TIC spectra, ESI-MS spectra.

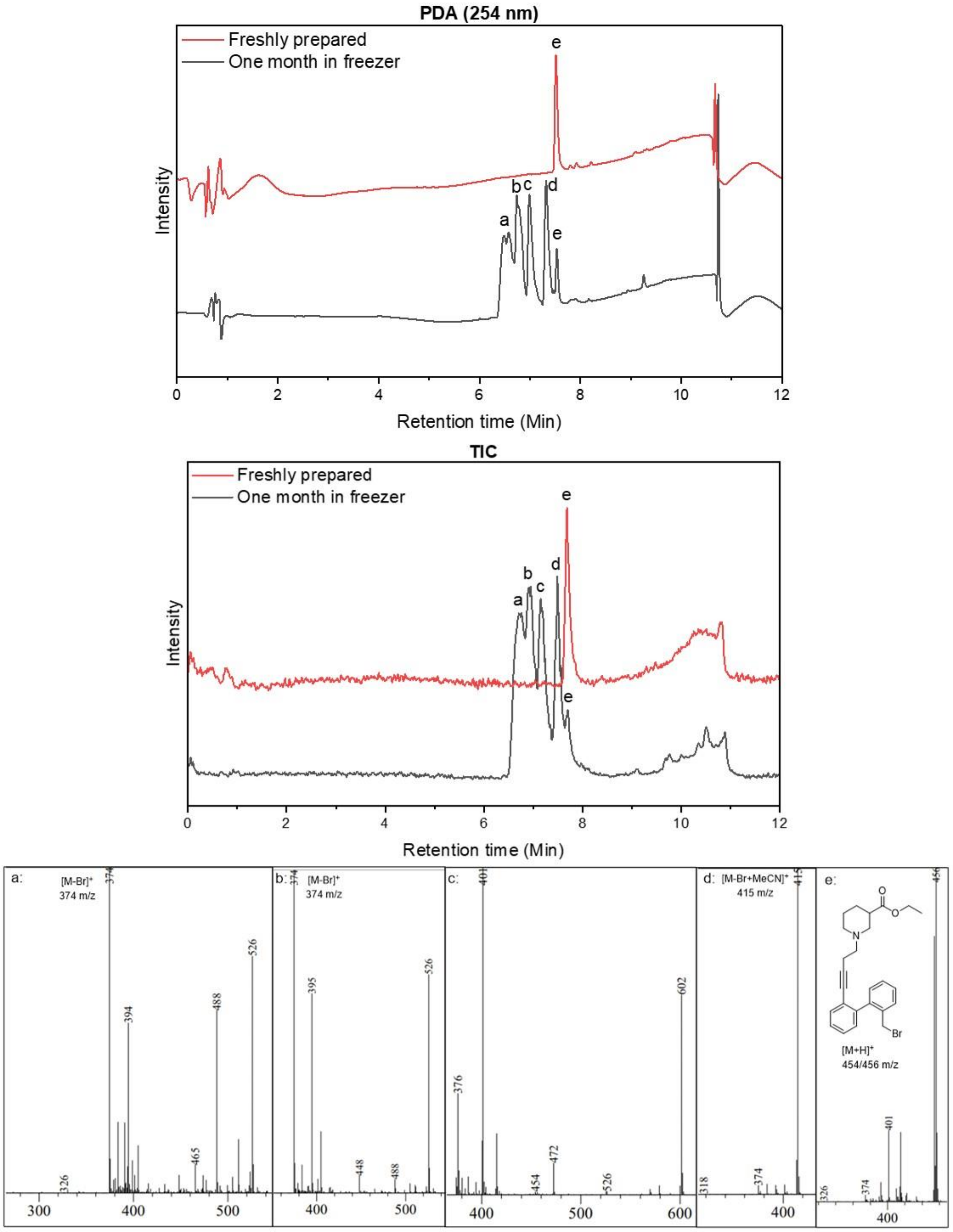

Figure S11: LC-MS spectra of compound **10a** directly after synthesis (red) and after storing one month in the freezer (black). Top to bottom: PDA spectra (254 nm), TIC spectra, ESI-MS spectra.

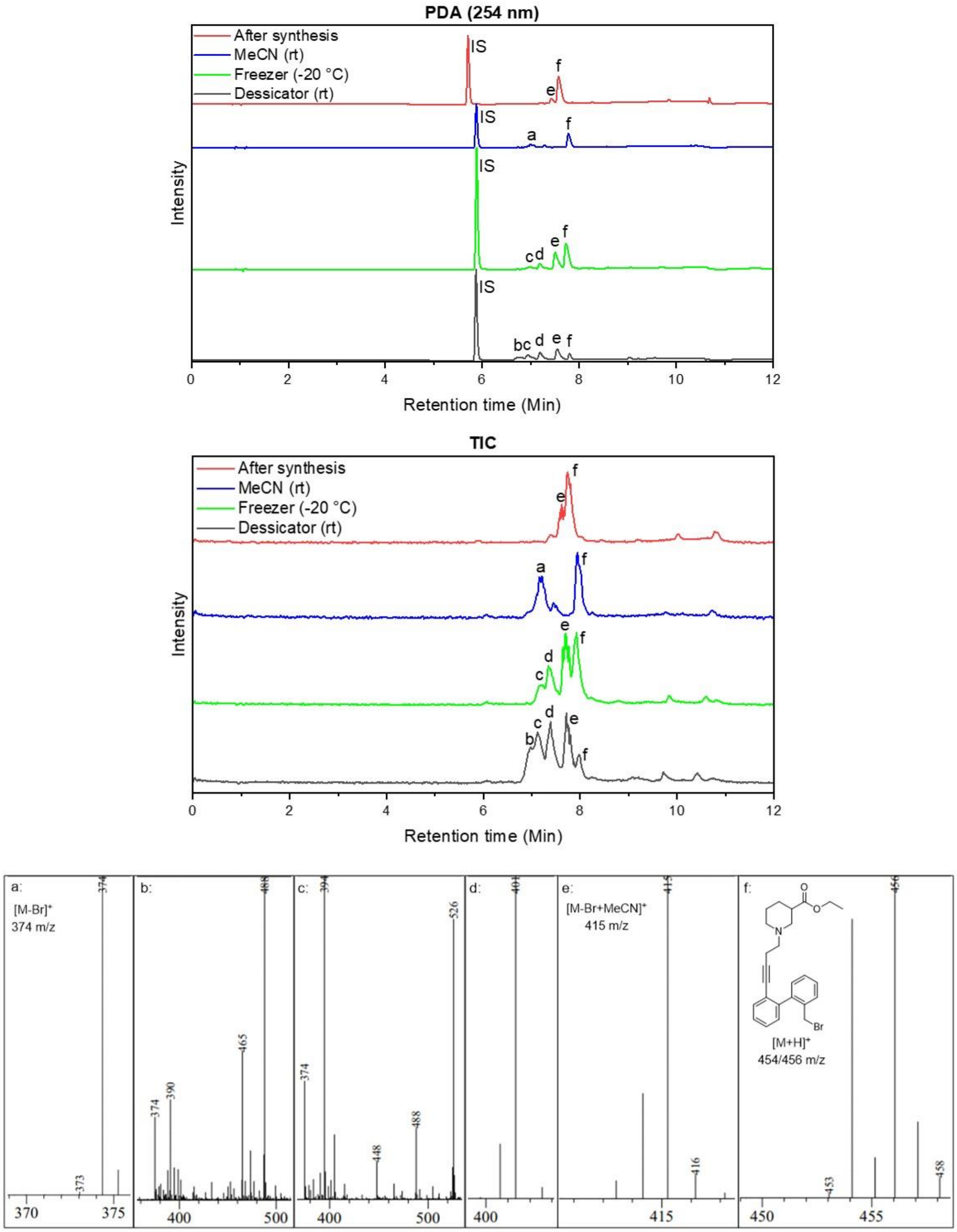

Figure S12: LC-MS spectra of compound **10a** directly after synthesis (red) and after storing for one – seven days in MeCN (blue), the freezer (green), and the desiccator under vacuum (black). Top to bottom: PDA spectra (254 nm), TIC spectra, ESI-MS spectra. After synthesis, the product **10a** was divided into batches of 1 mg, which were stored as indicated. Upon sample preparation, MeCN (670 µL) and internal standard (IS) solution (830 µL, 75 µM 2-nitrobenzoic acid) were added and the samples were measured by LC-MS.

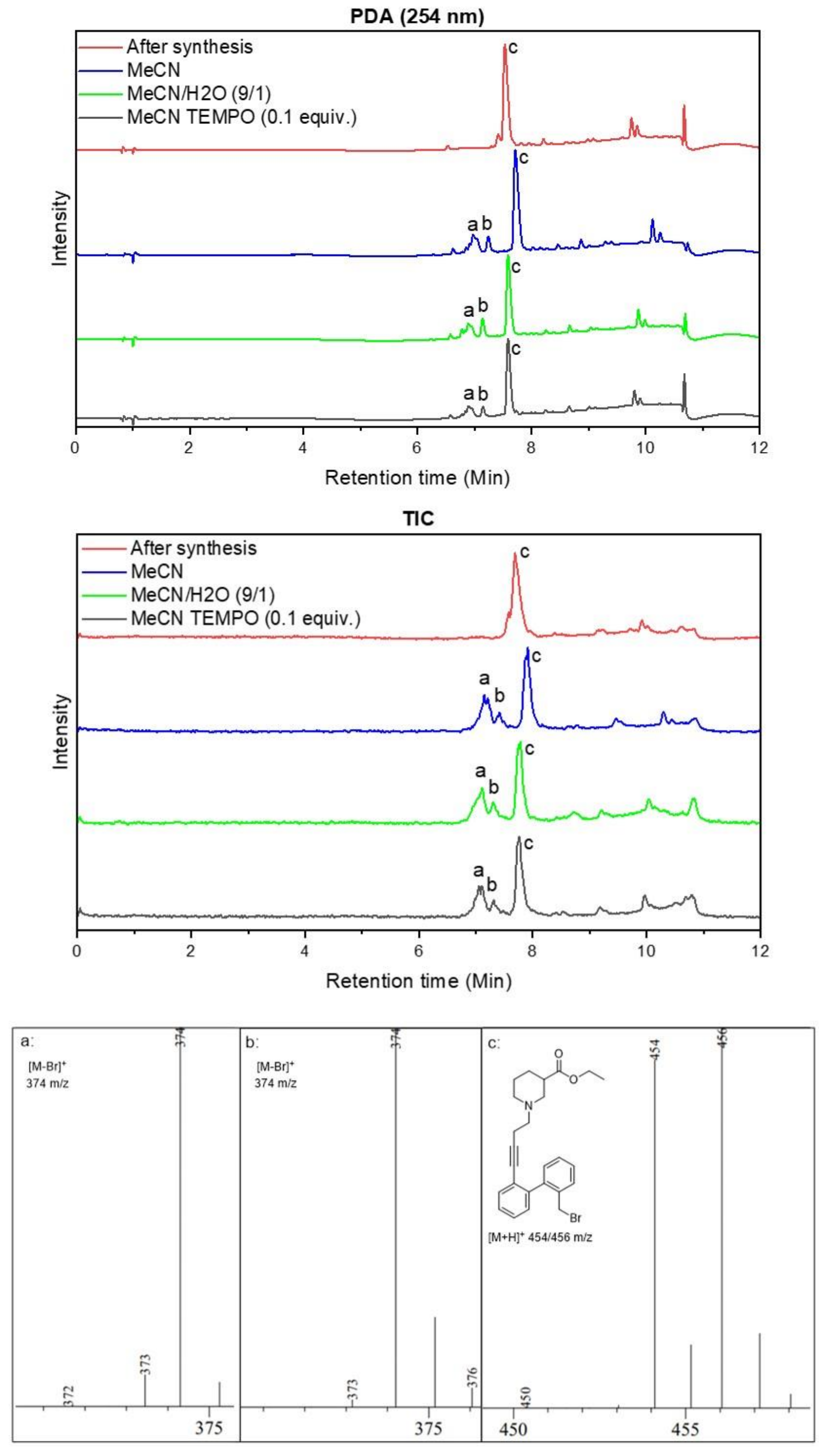

Figure S13: LC-MS spectra of compound **10a** directly after synthesis (red) and after storing for one week in MeCN (blue), MeCN/H_2O 9/1 (green), and MeCN with 0.1 equiv. TEMPO (black). Top to bottom: PDA spectra (254 nm), TIC spectra, ESI-MS spectra.

Deoxyfluorination reactions using the model system biphenyl-4-methanol

To obtain the fluorinated reference compounds **11a-b**, several deoxyfluorination conditions were screened using the model system biphenyl-4-methanol (**S1**) (**Scheme S3**). Given the successful deoxyfluorination of benzylic alcohols, including biphenyl-4-methanol, using the $PPh_3/ICH_2CH_2I/CsF$ system by Xiao and co-workers, [7] these reaction conditions were first investigated. Unfortunately, these conditions only afforded moderate conversion and we continued using DAST. Minimal optimization afforded complete conversion, after which these reaction conditions were used for the synthesis of the fluorinated reference standards **11a-b**.

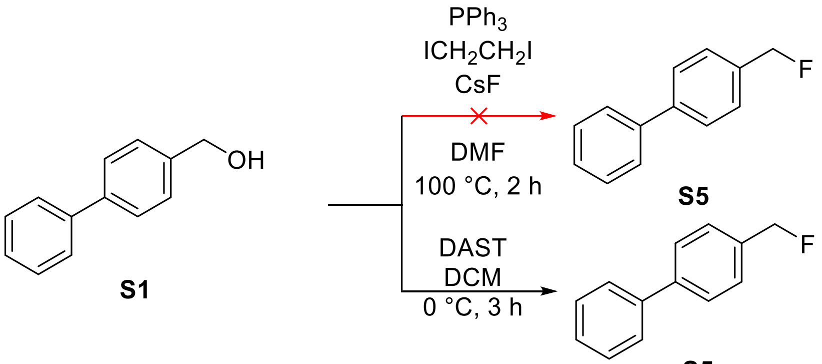

Scheme S3: Deoxyfluorination test reactions using the model system biphenyl-4-methano (**S1**).

First a deoxyfluorination was performed using the $PPh_3/ICH_2CH_2I/CsF$ system by Xiao and co-workers.[7] To that end, anhydrous DMF (5 mL) was degassed using three freeze-pump-thaw cycles in an oven-dried Schlenk flask. Afterwards, biphenyl-4-methanol (**S1**) (92 mg, 0.5 mmol, 1.0 equiv.), PPh_3 (197 mg, 0.75 mmol, 1.5 equiv.), 1,2-diiodoethane (211 mg, 0.75 mg, 1.5 equiv.) were added under nitrogen atmosphere and the mixture was stirred until all compounds had dissolved. Dried (vacuum oven, 100 °C, on) CsF (228 mg, 1.5 mmol, 3.0 equiv.) was added and the reaction mixture was placed in a pre-heated oil bath and stirred at 100 °C for 2 h. Then, DCM (20 mL) was added, and the organic layer was washed with water (3 x 20 mL). The organic layer was dried over $MgSO_4$ after which the solvent was removed *in vacuo* to obtain the crude product. NMR analysis indicated the formation of the product **S5** (^{1}H NMR: 5.41 ppm, d, J = 47.9 Hz; ^{19}F NMR: -205.93 ppm, t, J = 47.6 Hz) [8], though starting material **S1** (^{1}H NMR: 4.72 ppm) and side-products (^{1}H NMR: 5.24 ppm, 4.78 ppm) were also visible (**Figure S13-14**).

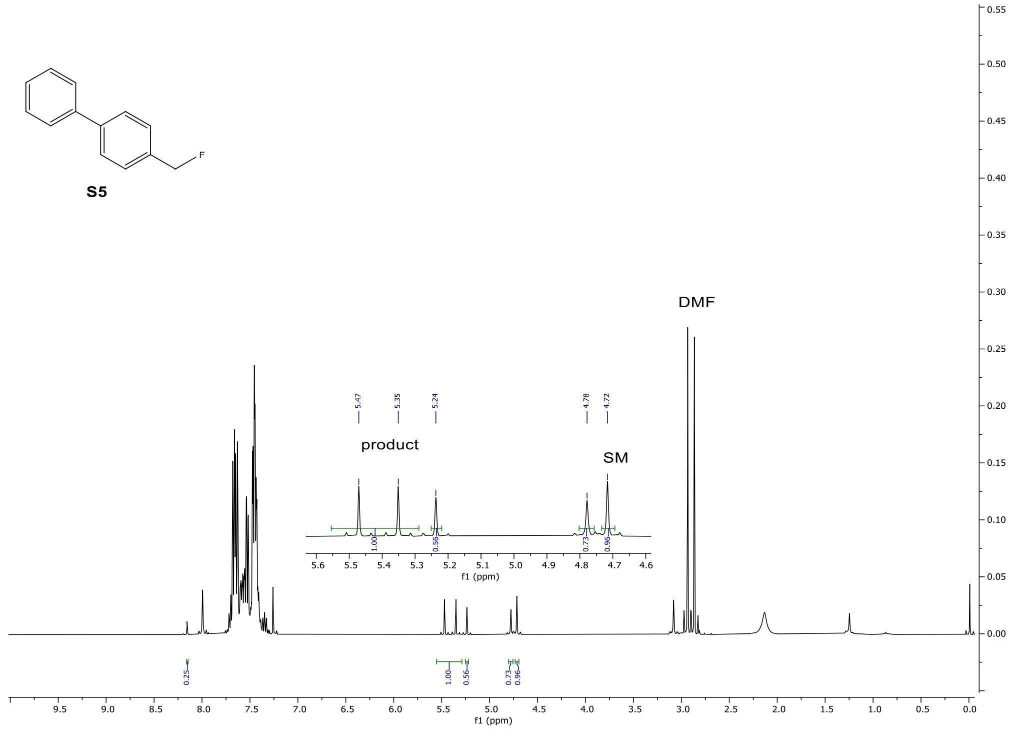

Figure S13: Crude ^{1}H NMR spectrum ($CDCl_3$, 400 MHz) of 4-(fluoromethyl)-1,1'-biphenyl (**S5**). SM = starting material **S1**.

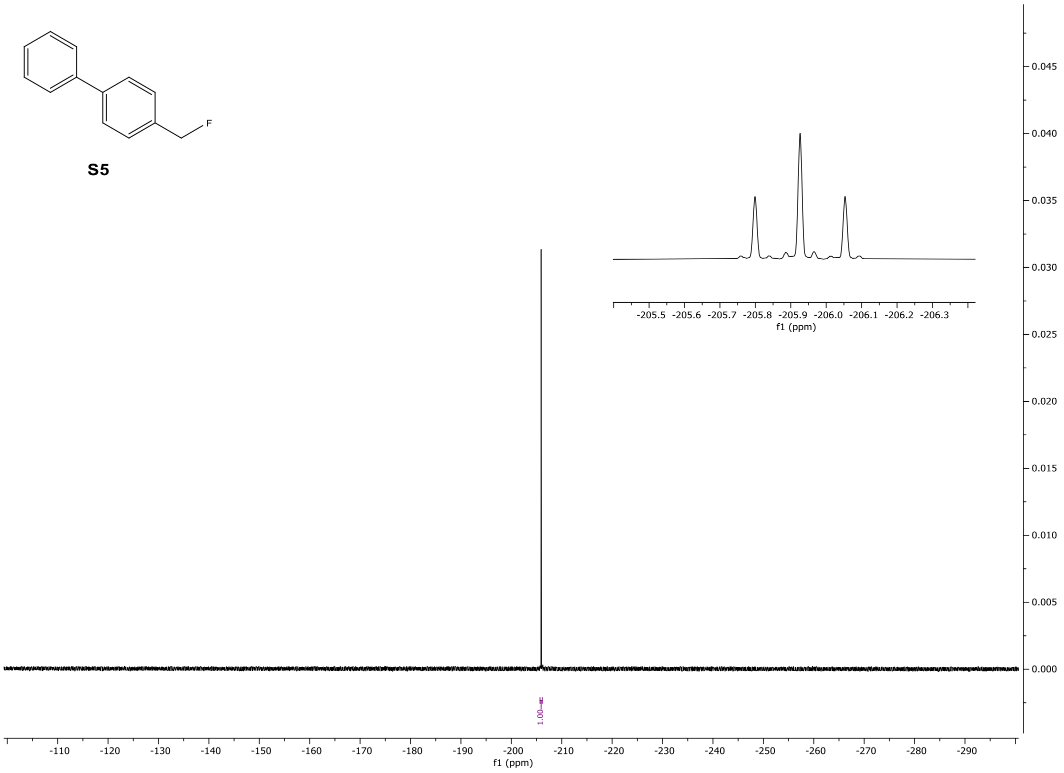

Figure S14: Crude ^{19}F NMR spectrum ($CDCl_3$, 376 MHz) of 4-(fluoromethyl)-1,1'-biphenyl (**S5**).

Since the $PPh_3/ICH_2CH_2I/CsF$ system did not afford completion of the reaction in our hands, DAST was used to perform deoxyfluorination reactions. In this procedure biphenyl-4-methanol (**S1**) (100 mg, 0.54 mmol, 1.0 equiv.) was introduced into an oven-dried Schlenk tube and the atmosphere was exchanged to N_2. Anhydrous DCM (3 mL) was then added and the solution was cooled in an ice bath. Afterwards, DAST (amount in **Table S1**) was added and the reaction mixture was stirred at room temperature for the time indicated in **Table S1**. The reaction was quenched by addition of ice and the mixture was stirred until the ice melted. Then, the product was extracted with DCM. The combined organic layers were washed with brine and dried over $MgSO_4$, after which the solvent was removed *in vacuo* to obtain the crude product. NMR analysis of the crude product indicated the formation of the product **S5** (^{1}H NMR: 5.43 ppm, d, J = 47.9 Hz; ^{19}F NMR: -205.97 ppm, t, J = 47.9 Hz)[8], although side-products (^{1}H NMR: 4.64 ppm, 4.07 ppm) were also visible (**Figure S15-16**).

Minimal optimization of the conditions started using 3.5 equiv. DAST for 24 h at room temperature (**Table S1, Entry 1**). It was found that both decreasing the reaction time to 3 h (**Table S1, Entry 2**) and decreasing the amount of DAST to 2.0 equiv. **(Table S1, Entry 3**) had no effect on the product distribution. Therefore, the last conditions were used to access the reference standards **11a-b**.

Table S1: Optimization of deoxyfluorination of biphenyl-4-methanol (**S1**)

Entry	DAST (equiv.)	Conditions	Product integration (5.43 ppm)	Side-product integration (4.64 ppm)	Side-product integration (4.07 ppm)
1	3.5	24 h, rt	2H	0.37H	0.29H
2	3.5	3 h, rt	2H	0.39H	0.26H
3	2.0	3 h, rt	2H	0.39H	0.28H

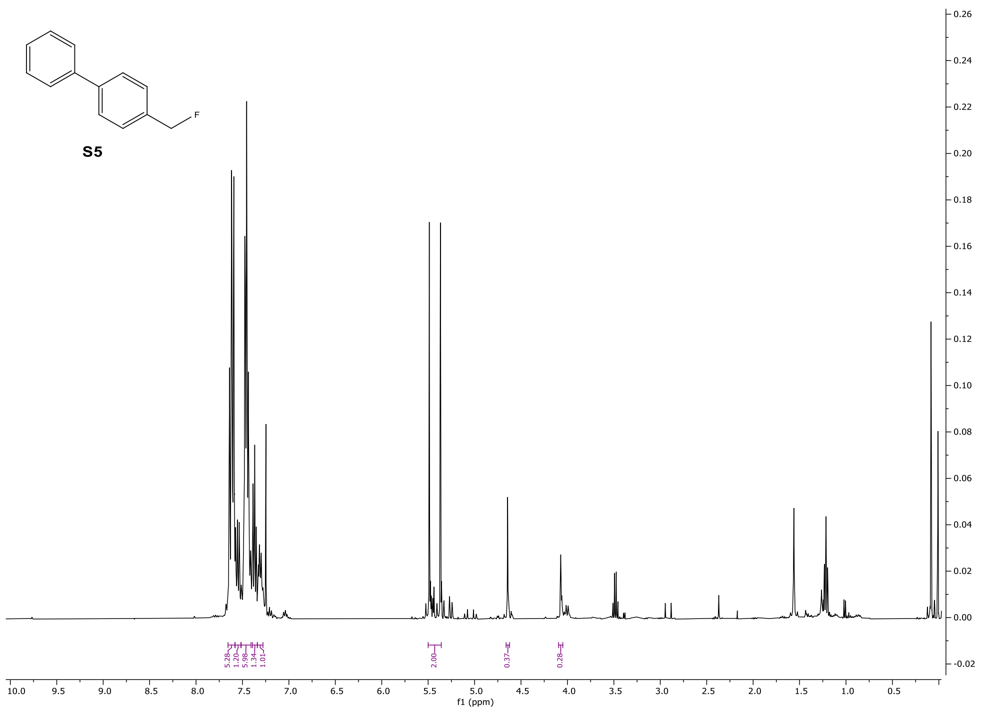

Figure S15: Crude ^{1}H NMR spectrum ($CDCl_3$, 400 MHz) of 4-(fluoromethyl)-1,1'-biphenyl (**S5**) for entry 1 in table S1.

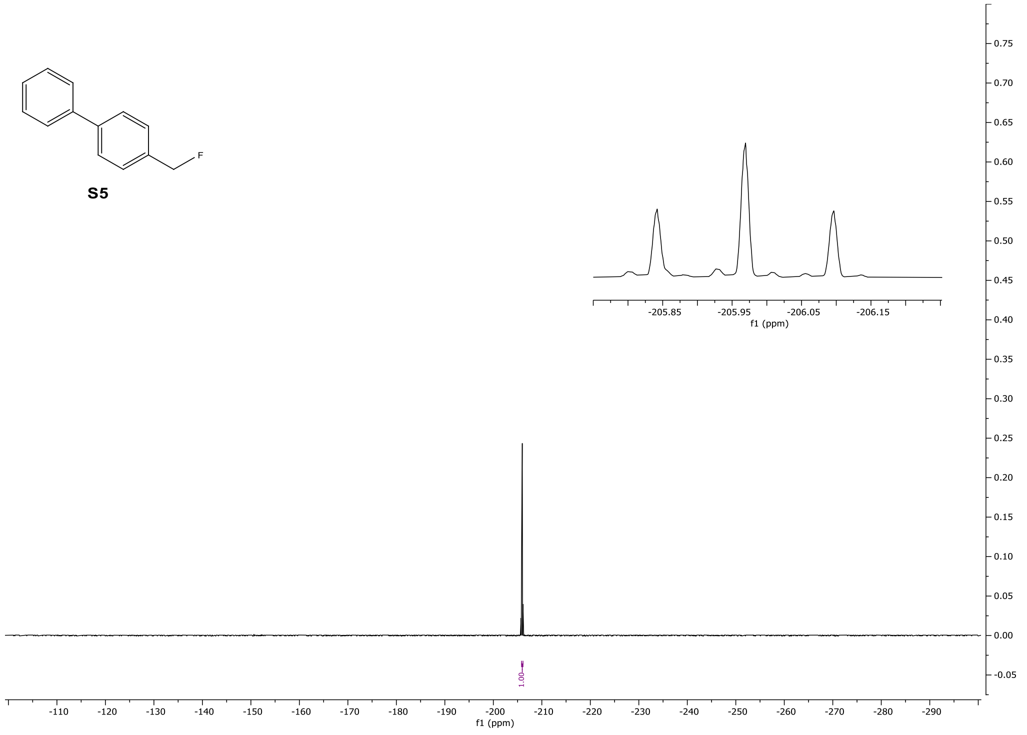

Figure S16: Crude ^{19}F NMR spectrum ($CDCl_3$, 376 MHz) of 4-(fluoromethyl)-1,1'-biphenyl (**S5**) for entry 1 in table S1.

Synthesis of reference standards **11a-b**

With an optimized fluorination procedure for the model system biphenyl-4-methanol in hand, this procedure was used to synthesize the fluorinated reference standards **11a-b**. Unfortunately, it was found that two products with the same mass were formed (**Figure S17**), mostly for the *tert*-butyl protected version **11b**. Treatment of benzylic alcohols with DAST is able to proceed through an S_N1 or S_N2-like mechanism. [9, 10] Moreover, carbocations with the same alkynylbiphenyl moiety have also been observed to react through an 6-*exo-dig* cyclization pathway. [11] Therefore, it was assumed that a similar 6-*exo-dig* cyclization with the alkyne (**Scheme 4**) could allow for the formation of **11a-b'** as plausible structure of the side-product.

Additional signs for the formation of this side-product could be found in the ^{1}H and ^{19}F NMR spectra that were recorded after isolation of a small fraction of the side-product (**Figures S18-19**). ^{1}H NMR shows a singlet at 3.68 ppm, which is in range for the CH_2 protons of 9-methylene-9,10-dihydrophenanthrene moieties (reported 3.65 ppm) [12]. Moreover, the ^{19}F NMR signal at -105.6 ppm is close to other reported fluoroalkenes. [13, 14]

In an attempt to minimize the formation of side-product **11b'** it was decided to add CsF (2.0 equiv.) to the reaction mixture to accelerate direct fluorination to give **11b** instead of stabilization of the carbocation with the alkyne leading to generation of **11b'**. To our delight, this proved effective (**Figure S17**) allowing the synthesis of reference compound **11b**.

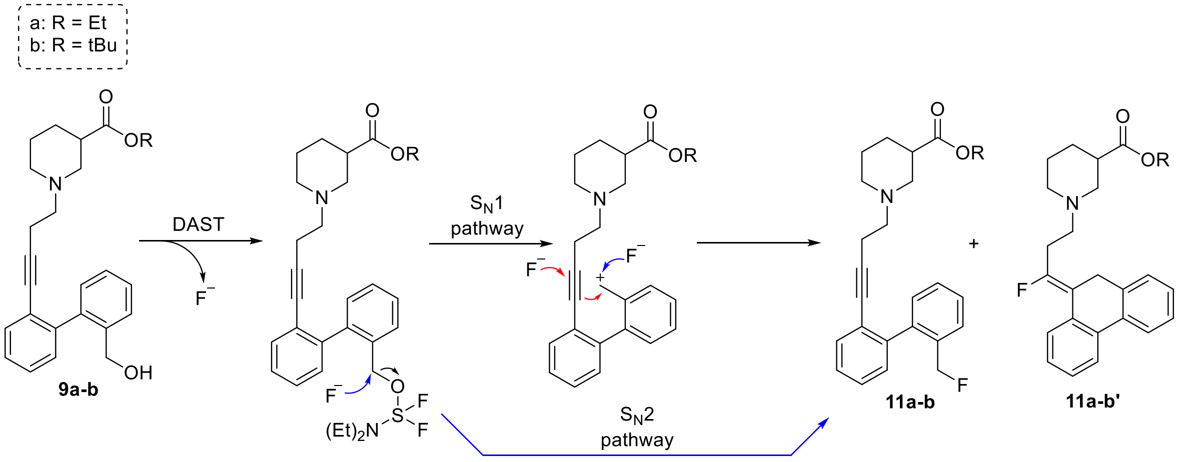

Scheme S4: DAST-mediated deoxyfluorination to afford **11a-b** (blue arrows) and presumed cyclodehydration to afford **11a-b'** (red arrows).

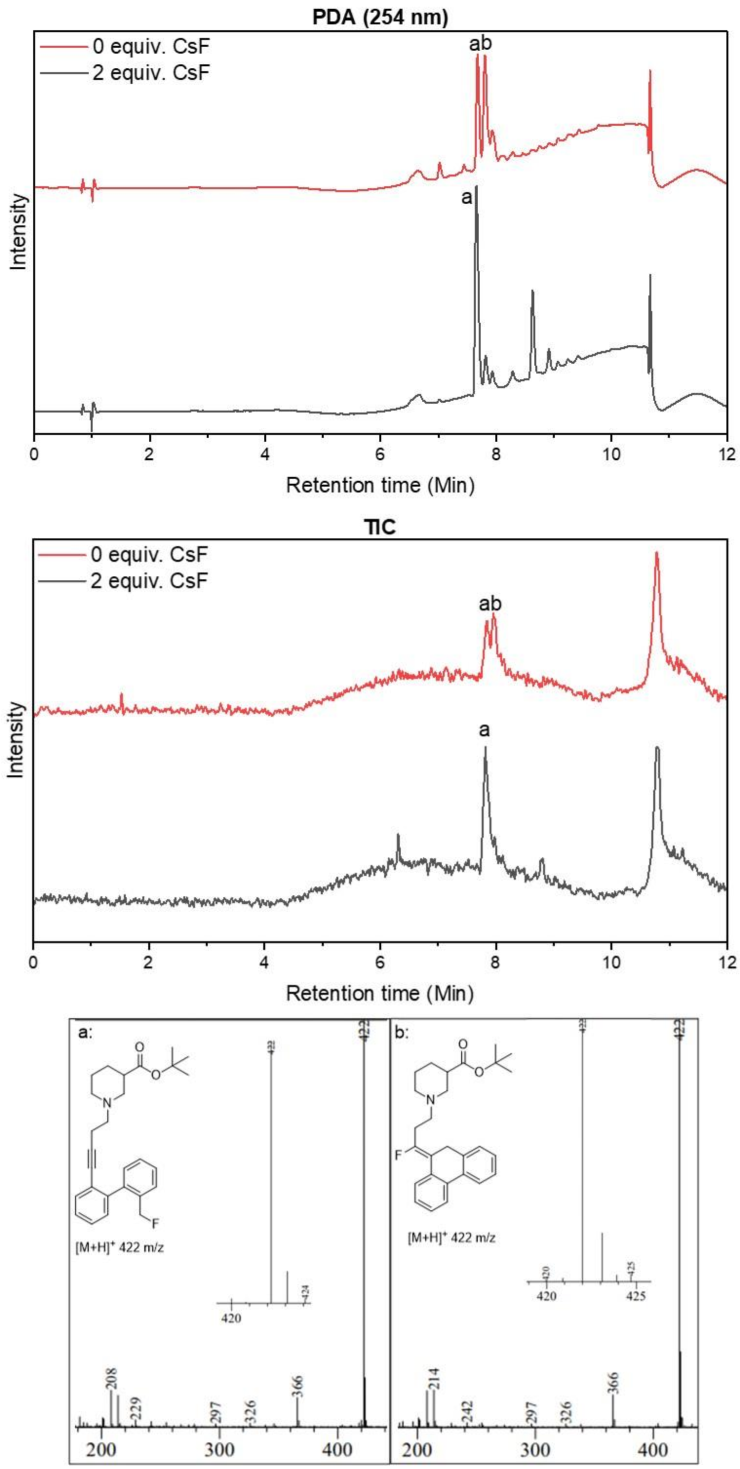

Figure S17: LC-MS spectra of compound **11b** using no CsF (red) and using 2.0 equiv. CsF (black). Top to bottom: PDA spectra (254 nm), TIC spectra, ESI-MS spectra.

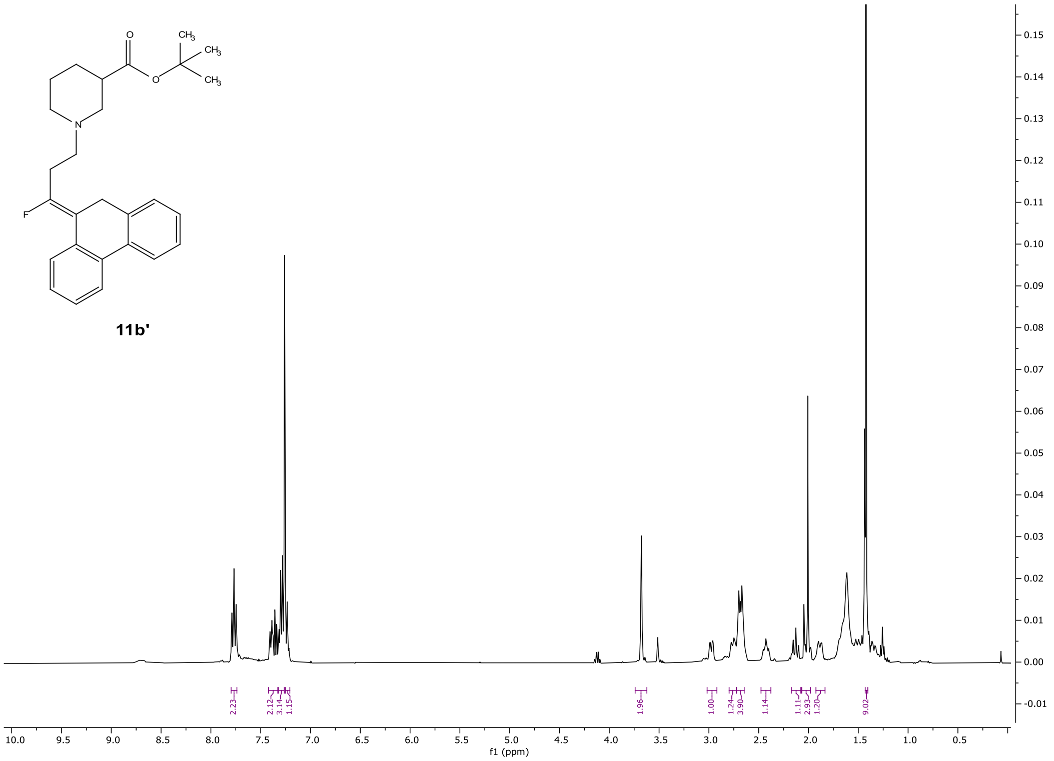

Figure S18: ^{1}H NMR spectrum ($CDCl_3$, 400 MHz) after isolation of a small fraction of the presumed side-product **11b'**.

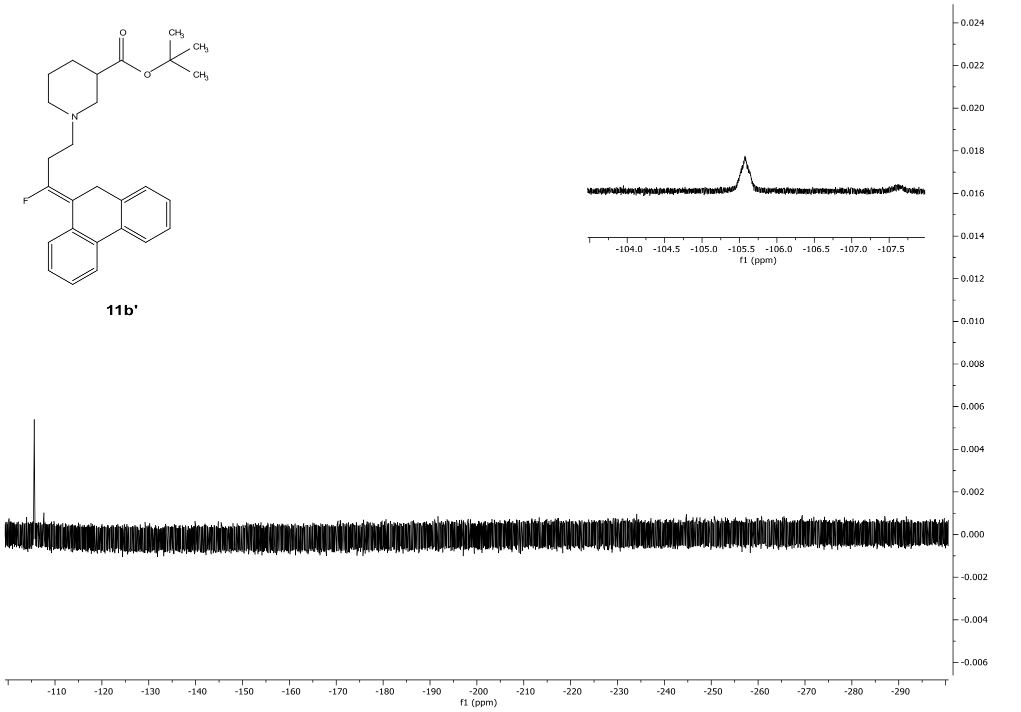

Figure S19: ^{19}F NMR spectrum ($CDCl_3$, 376 MHz) after isolation of a small fraction of the presumed side-product **11b'**.

Radiosynthesis

Ethyl ester deprotection

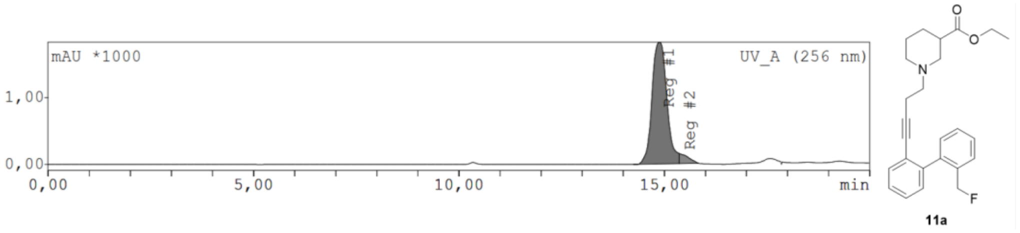

Figure S20: HPLC spectrum of reference compound **11a** (Reg #1, 14.9 min).

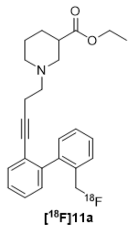

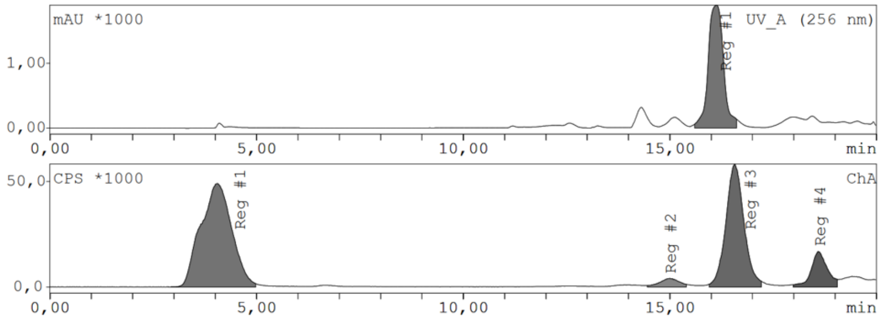

Integration ChA

Substance	R/T min	Type	Area Counts	%Area %
Reg #1	4,05	DD(	2576945	51,96
Reg #2	14,98	DD(	138221	2,79
Reg #3	16,57	DD(	1782106	35,93
Reg #4	18,60	DD(	462563	9,33
Sum in ROI			4959835	100,00
Area (total)			5512432	
Ext. BKG.			0,00 CPS	

Figure S21: HPLC spectrum of **[^{18}F]11a** (Reg #2, 15.0 min). Top to bottom: PDA spectrum (256 nm) and radioactivity trace.

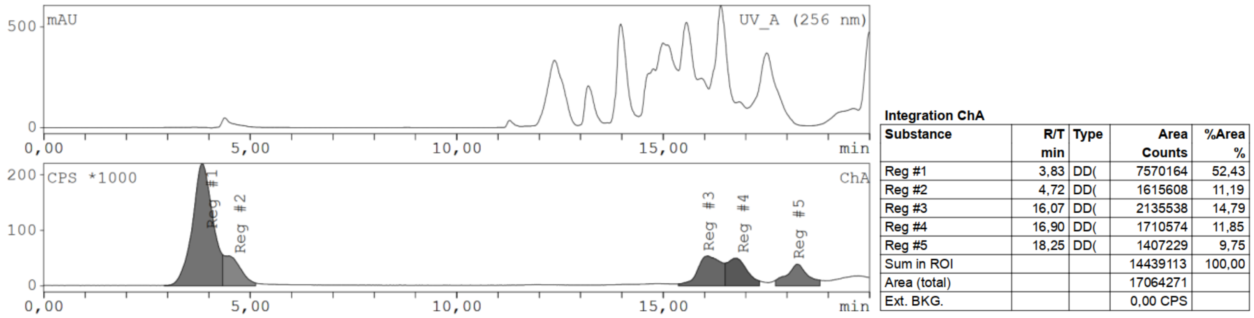

Integration ChA

Substance	R/T min	Type	Area Counts	%Area %
Reg #1	3,83	DD(	7570164	52,43
Reg #2	4,72	DD(	1615608	11,19
Reg #3	16,07	DD(	2135538	14,79
Reg #4	16,90	DD(	1710574	11,85
Reg #5	18,25	DD(	1407229	9,75
Sum in ROI			14439113	100,00
Area (total)			17064271	
Ext. BKG.			0,00 CPS	

Figure S22: HPLC spectrum after deprotection of **[^{18}F]11a** using 200 µL aq. 0.01 M NaOH, 100 °C, 20 min. No starting material **[^{18}F]11a** (15.0 min) or deprotected product **[^{18}F]4** (slightly more polar, so at lower retention time) can be observed. Instead, only degradation products are detected. Top to bottom: PDA spectrum (256 nm) and radioactivity trace.

Tert-butyl ester deprotection

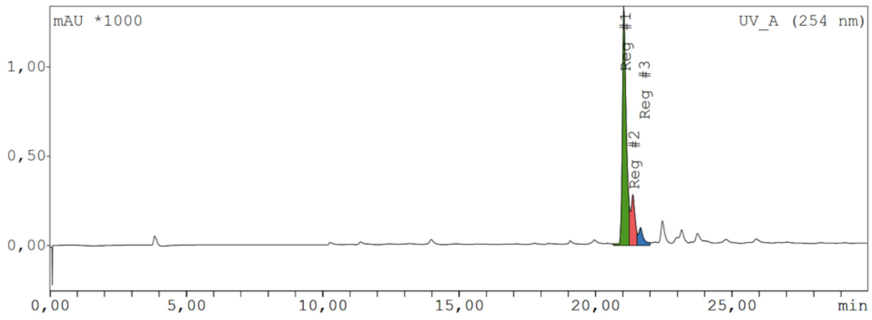

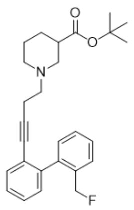

11b

Integration UV_A (254 nm)

Substance	R/T min	Type	Area mAU*min	%Area %
Reg #1	21,05	DD(	13331,66	73,06
Reg #2	21,37	DD(	3469,30	19,01
Reg #3	21,75	DD(	1446,84	7,93
Sum in ROI			18247,81	100,00

Figure S23: HPLC spectrum of reference compound **11b** (Reg #1, 21.1 min).

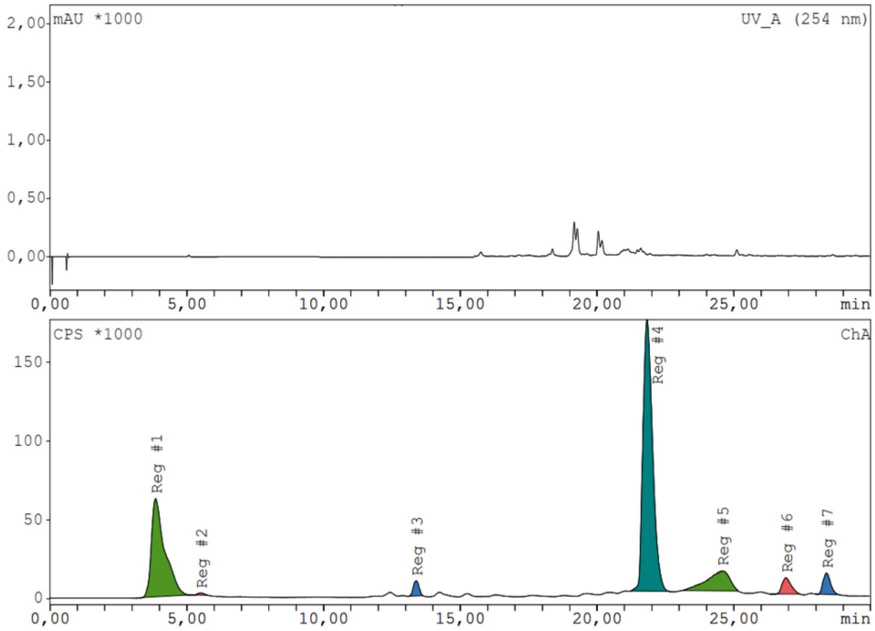

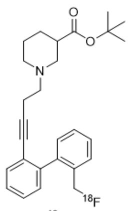

[^{18}F]11b

Integration ChA

Substanz	Ret min	Typ	Fläche Counts	%Fläche %
Reg #1	3,87	BB(	2110017	27,36
Reg #2	5,50	BB(	20644	0,27
Reg #3	13,38	BB(	138556	1,80
Reg #4	21,83	BB(	4219478	54,72
Reg #5	24,57	BB(	731788	9,49
Reg #6	26,90	BB(	235343	3,05
Reg #7	28,38	BB(	254918	3,31
Summe in ROI			7710744	100,00
Fläche (total)			11715174	
Ext. BKG.			0,00 CPS	

Figure S24: HPLC spectrum of **[^{18}F]11b** (Reg #4, 21.8 min). Top to bottom: PDA spectrum (254 nm) and radioactivity trace.

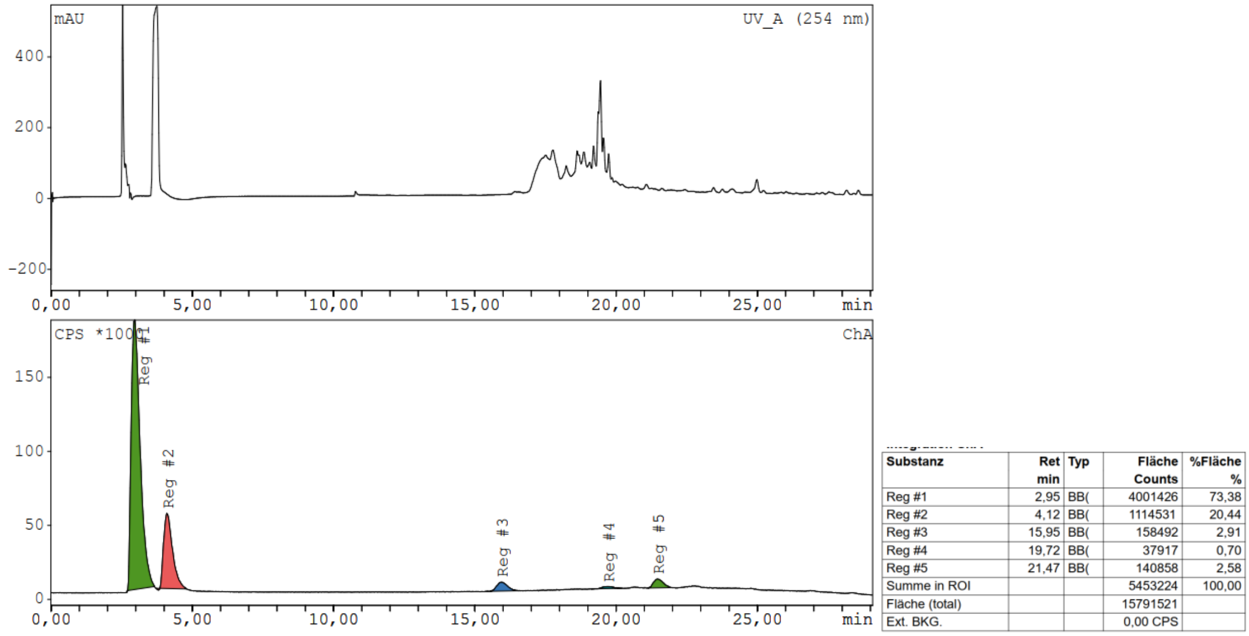

Substanz	Ret min	Typ	Fläche Counts	%Fläche %
Reg #1	2,95	BB(	4001426	73,38
Reg #2	4,12	BB(	1114531	20,44
Reg #3	15,95	BB(	158492	2,91
Reg #4	19,72	BB(	37917	0,70
Reg #5	21,47	BB(	140858	2,58
Summe in ROI			5453224	100,00
Fläche (total)			15791521	
Ext. BKG.			0,00 CPS	

Figure S25: HPLC spectrum after deprotection of **[^{18}F]11b** using 150 µL 2 M HCl, 10 min, 105 °C. The starting material **[^{18}F]11b** (21.5 min) almost completely disappeared. However, only free fluor is being detected around 3 min and no product formation is detected. Top to bottom: PDA spectrum (254 nm) and radioactivity trace.

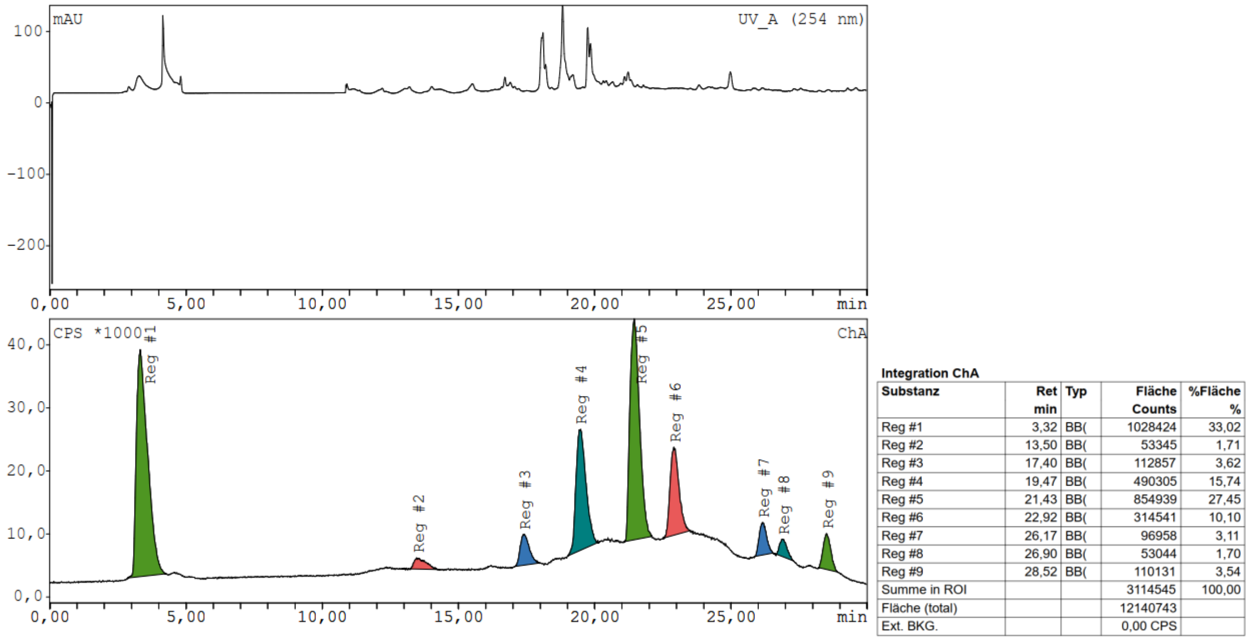

Integration ChA

Substanz	Ret min	Typ	Fläche Counts	%Fläche %
Reg #1	3,32	BB(	1028424	33,02
Reg #2	13,50	BB(	53345	1,71
Reg #3	17,40	BB(	112857	3,62
Reg #4	19,47	BB(	490305	15,74
Reg #5	21,43	BB(	854939	27,45
Reg #6	22,92	BB(	314541	10,10
Reg #7	26,17	BB(	96958	3,11
Reg #8	26,90	BB(	53044	1,70
Reg #9	28,52	BB(	110131	3,54
Summe in ROI			3114545	100,00
Fläche (total)			12140743	
Ext. BKG.			0,00 CPS	

Figure S26: HPLC spectrum after deprotection of **[^{18}F]11b** using 150 µL TFA, 10 min, rt. Most of the product is still protected as **[^{18}F]11b** (Reg #5, 21.4 min). Top to bottom: PDA spectrum (254 nm) and radioactivity trace.

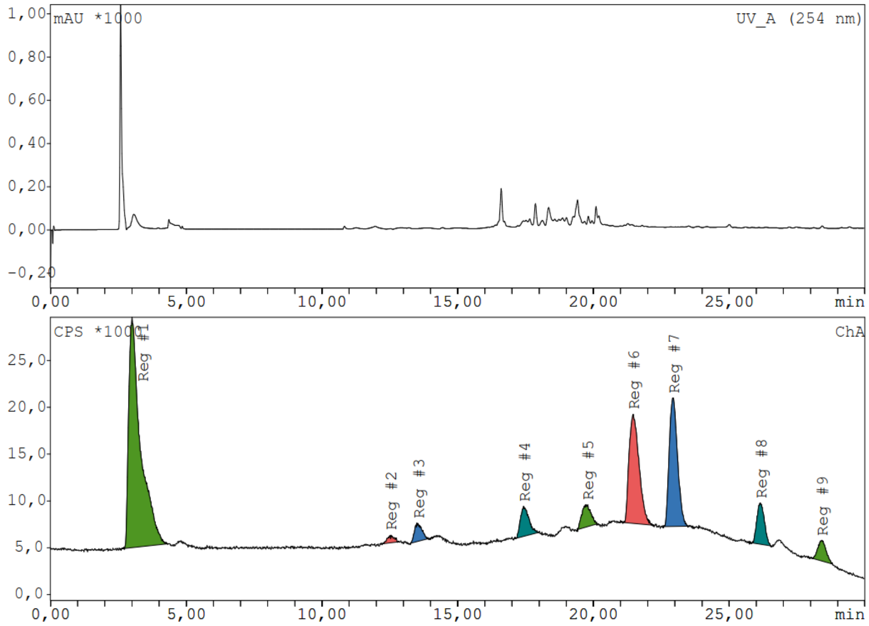

Integration ChA

Substanz	Ret min	Typ	Fläche Counts	%Fläche %
Reg #1	3,00	BB(	742299,4	45,57
Reg #2	12,52	BB(	12778,3	0,78
Reg #3	13,53	BB(	34993,6	2,15
Reg #4	17,43	BB(	68251,4	4,19
Reg #5	19,77	BB(	54386,5	3,34
Reg #6	21,45	BB(	297457,5	18,26
Reg #7	22,93	BB(	288196,7	17,69
Reg #8	26,15	BB(	84161,9	5,17
Reg #9	28,40	BB(	46342,2	2,85
Summe in ROI			1628867,4	100,00
Fläche (total)			11596078,	
Ext. BKG.			0,00 CPS	

Figure S27: HPLC spectrum after deprotection of **[^{18}F]11b** using 150 µL TFA, 20 min, 105 °C. **[^{18}F]11b** is still present (Reg #6, 21.5 min). Top to bottom: PDA spectrum (254 nm) and radioactivity trace.

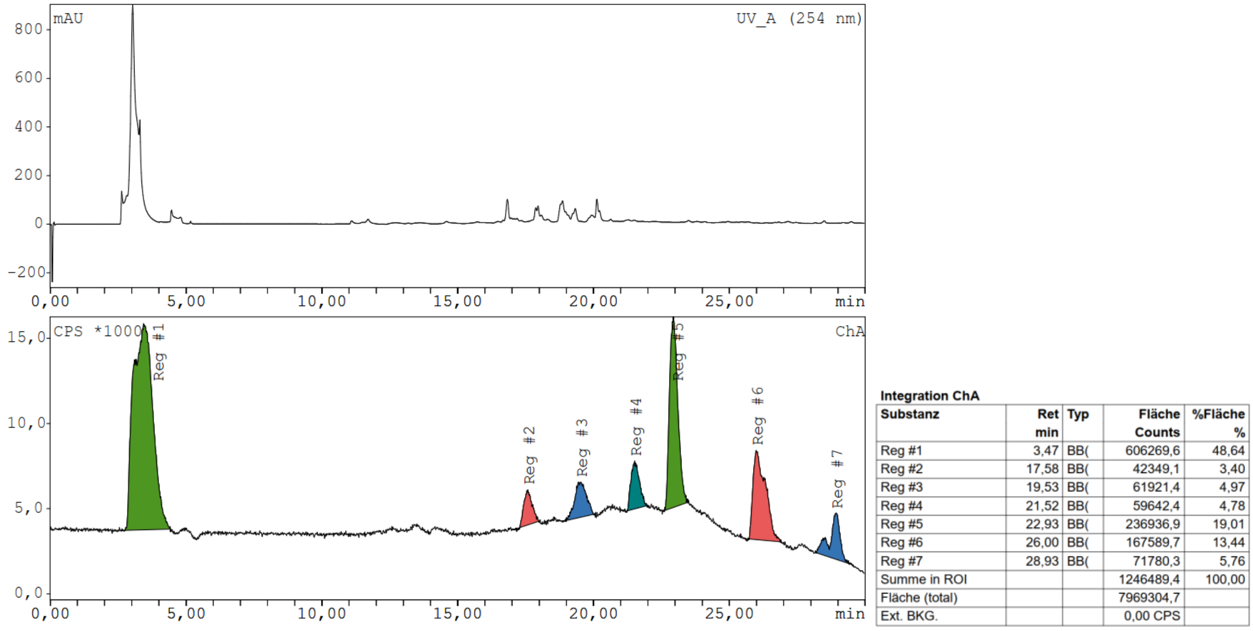

Integration ChA

Substanz	Ret min	Typ	Fläche Counts	%Fläche %
Reg #1	3,47	BB(	606269,6	48,64
Reg #2	17,58	BB(	42349,1	3,40
Reg #3	19,53	BB(	61921,4	4,97
Reg #4	21,52	BB(	59642,4	4,78
Reg #5	22,93	BB(	236936,9	19,01
Reg #6	26,00	BB(	167589,7	13,44
Reg #7	28,93	BB(	71780,3	5,76
Summe in ROI			1246489,4	100,00
Fläche (total)			7969304,7	
Ext. BKG.			0,00 CPS	

Figure S28: HPLC spectrum after deprotection of **[^{18}F]11b** using 250 µL formic acid, 15 min, 50 °C. **[^{18}F]11b** is still present (Reg #4, 21.5 min). Top to bottom: PDA spectrum (254 nm) and radioactivity trace.

Ethyl ester prodrug approach

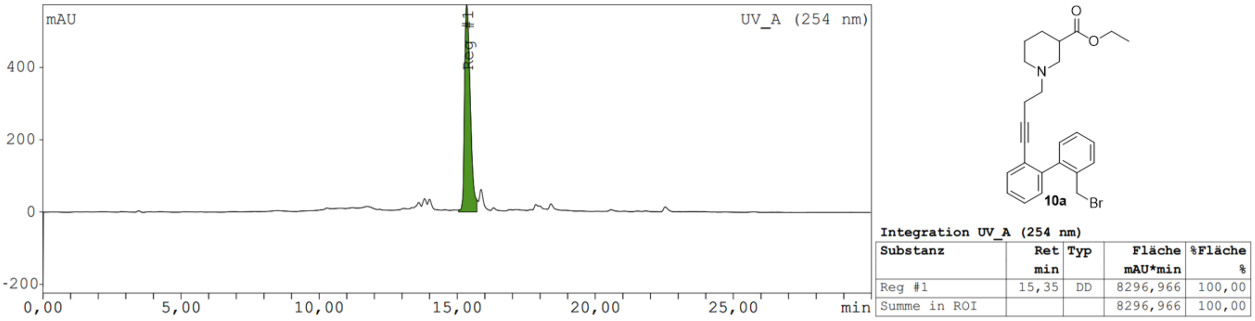

Integration UV_A (254 nm)

Substanz	Ret min	Typ	Fläche mAU*min	%Fläche %
Reg #1	15,35	DD	8296,966	100,00
Summe in ROI			8296,966	100,00

Figure S29: HPLC spectrum of brominated precursor **10a** using the semi-preparative column under conditions used for purification.

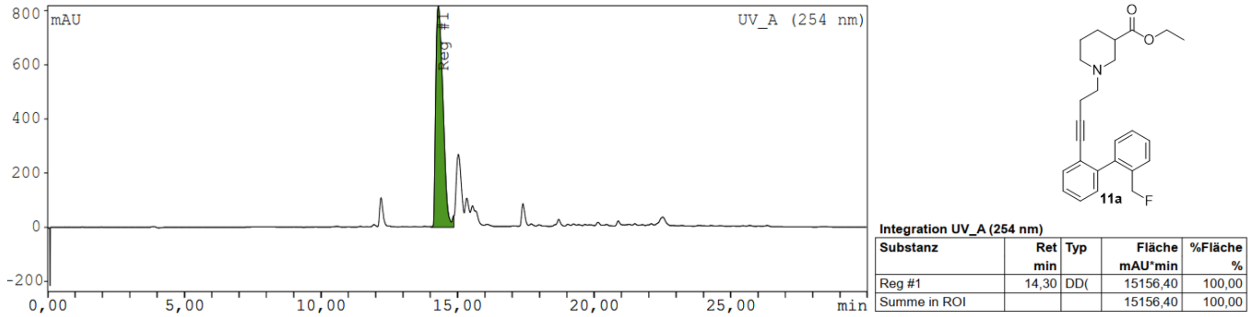

Integration UV_A (254 nm)

Substanz	Ret min	Typ	Fläche mAU*min	%Fläche %
Reg #1	14,30	DD(	15156,40	100,00
Summe in ROI			15156,40	100,00

Figure S30: HPLC spectrum of reference compound **11a** using the semi-preparative column under conditions used for purification.

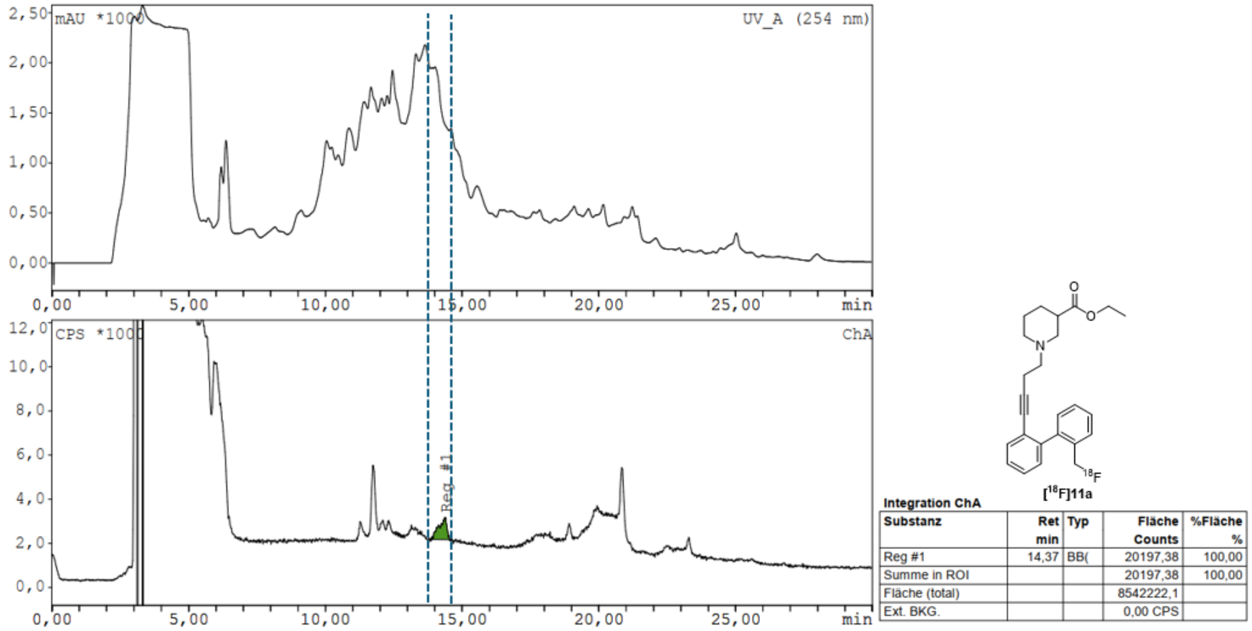

Integration ChA

Substanz	Ret min	Typ	Fläche Counts	%Fläche %
Reg #1	14,37	BB(	20197,38	100,00
Summe in ROI			20197,38	100,00
Fläche (total)			8542222,1	
Ext. BKG.			0,00 CPS	

Figure S31: HPLC spectrum obtained during the purification of **[^{18}F]11a**. The region between the dotted lines was collected. Top to bottom: PDA spectrum (254 nm) and radioactivity trace.

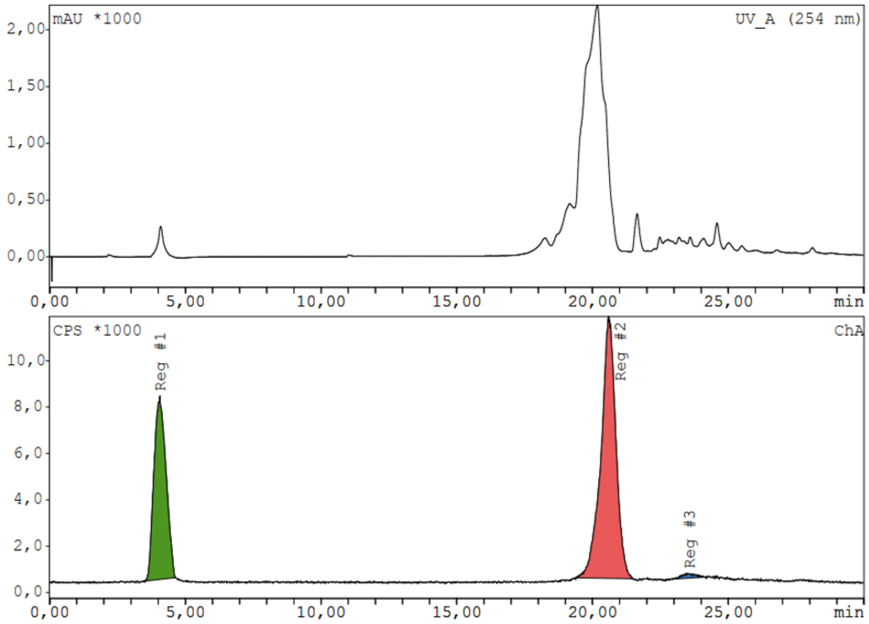

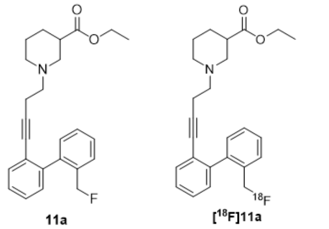

11a [18F]**11a**

Integration ChA

Substanz	Ret min	Typ	Fläche Counts	%Fläche %
Reg #1	4,03	BB(	244303,8	35,96
Reg #2	20,60	BB(	428612,7	63,08
Reg #3	23,53	BB(	6534,1	0,96
Summe in ROI			679450,6	100,00
Fläche (total)			1573307,8	
Ext. BKG.			0,00 CPS	

Figure S32: Co-injection of purified **[18F]11a** and reference standard **11a** 45 minutes after purification. The tracer **[18F]11a** (Reg #2, 20.6 min) partially degraded to free [18F]fluoride (Reg #1, 4.0 min). Top to bottom: PDA spectrum (254 nm) and radioactivity trace.

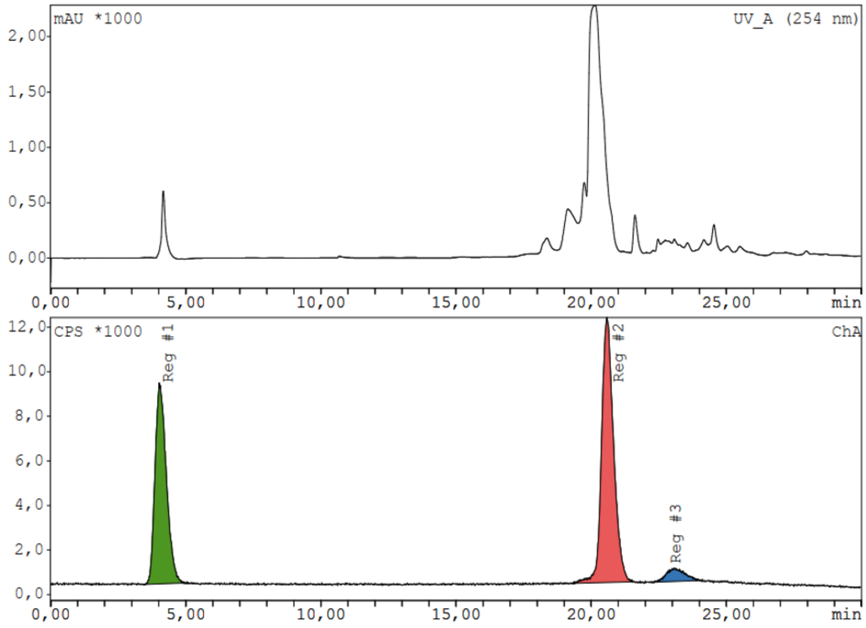

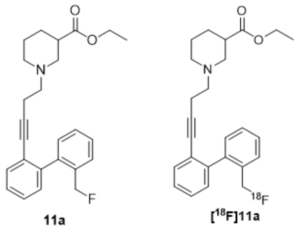

11a [18F]**11a**

Integration ChA

Substanz	Ret min	Typ	Fläche Counts	%Fläche %
Reg #1	4,03	BB(	261870,1	39,97
Reg #2	20,58	BB(	365893,9	55,84
Reg #3	23,08	BB(	27438,6	4,19
Summe in ROI			655202,6	100,00
Fläche (total)			1533440,6	
Ext. BKG.			0,00 CPS	

Figure S33: Co-injection of purified **[18F]11a** and reference standard **11a** 85 minutes after purification. The tracer **[18F]11a** (Reg #2, 20.6 min) partially degraded to free [18F]fluoride (Reg #1, 4.0 min). Top to bottom: PDA spectrum (254 nm) and radioactivity trace.

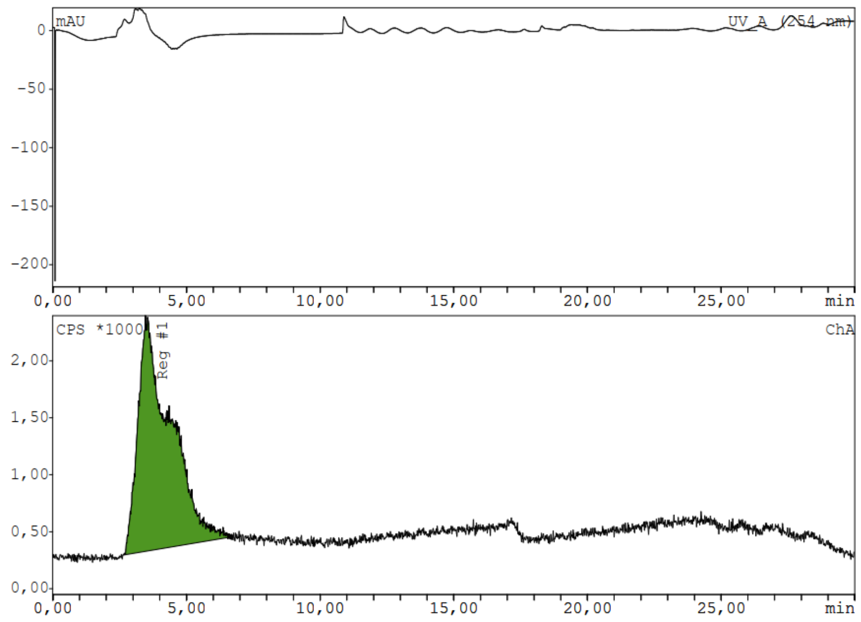

Integration ChA

Substanz	Ret min	Typ	Fläche Counts	%Fläche %
Reg #1	3,55	BB(	172743,1	100,00
Summe in ROI			172743,1	100,00
Fläche (total)			983082,7	
Ext. BKG.			0,00 CPS	

Figure S34: HPLC spectrum of [^{18}F]fluoride (5 µL in 500 µL MeCN). Top to bottom: PDA spectrum (254 nm) and radioactivity trace.

Molar activity estimation

As can be seen in the HPLC chromatogram obtained during purification of radiotracer **[^{18}F]11a** (Figure S31), several non-radioactive compounds eluted during the purification of the radiotracer in close proximity to the collected fractions. Therefore, precise quantification of the amount of isotopically stable compound was thwarted, making it difficult to calculate an accurate molar activity.

Nevertheless, an estimate the molar activity was made by assuming that all non-radioactive products that eluted with the radioligand **[^{18}F]11a** were the non-radioactive analogue **11a** or at least have a similar extinction coefficient. This allowed us to use the UV absorption in the HPLC taken during the purification of the radiotracer to make an estimate of the molar activity. To that end, an estimate of the amount of isotopically stable compound was then made using the integration of the UV-spectrum obtained during purification of the radiotracer **[^{18}F]11a** (Figure S35). Moreover, a calibration curve of the reference compound **11a** was made using the semi-preparative HPLC using the same conditions as during the purification (Figure S36). The amount of isotopically stable compound was then calculated to be approximately 0.91 µmol, using the linear fit of the linear range of the calibration curve. Using the final formulated dose of **[^{18}F]11a** (2.9 MBq), the molar activity value was then estimated to be 3.22 MBq/µmol

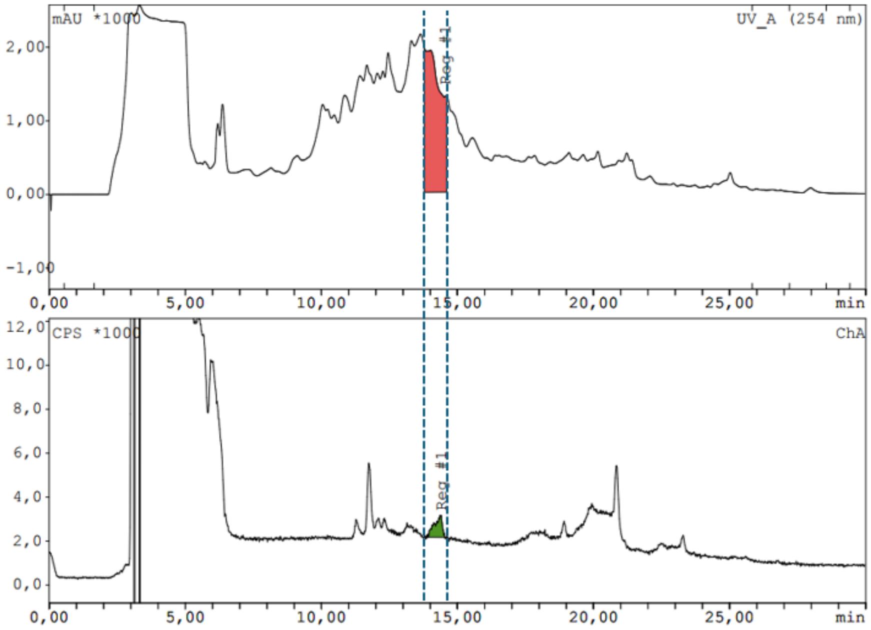

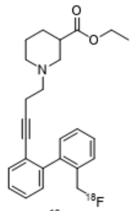

[^{18}F]11a

Integration UV_A (254 nm)

Substanz	Ret min	Typ	Fläche mAU*min	%Fläche %
Reg #1	14,52	DD	79975,30	100,00
Summe in ROI			79975,30	100,00
Fläche (total)			1130085	

Integration ChA

Substanz	Ret min	Typ	Fläche Counts	%Fläche %
Reg #1	14,37	BB	20197,38	100,00
Summe in ROI			20197,38	100,00
Fläche (total)			8542222	
Ext. BKG.			0,00 CPS	

Figure S35: HPLC spectrum obtained during the purification of **[^{18}F]11a** with integration of the co-eluted non-radioactive compounds. The region between the dotted lines was collected. Top to bottom: PDA spectrum (254 nm) and radioactivity trace.

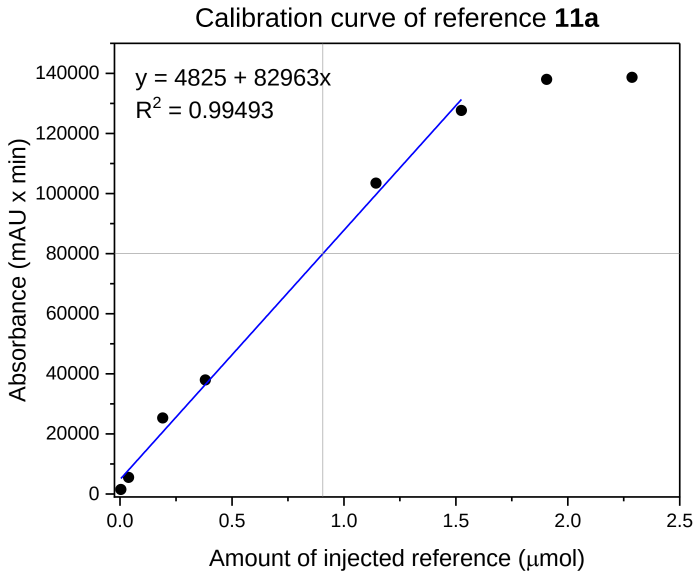

Figure S36: Calibration curve of non-radioactive reference standard **11a**. A linear fit (blue) was performed on the linear region of the calibration curve. The absorbance value for the integration of the UV-spectrum obtained during purification of the radiotracer **[^{18}F]11a** (79975.3 mAU x min) and the corresponding amount of isotopically stable compound (0.91 µmol) are indicated with grey lines.

Analytical spectra of synthesized compounds

Analytical spectra of **6a**

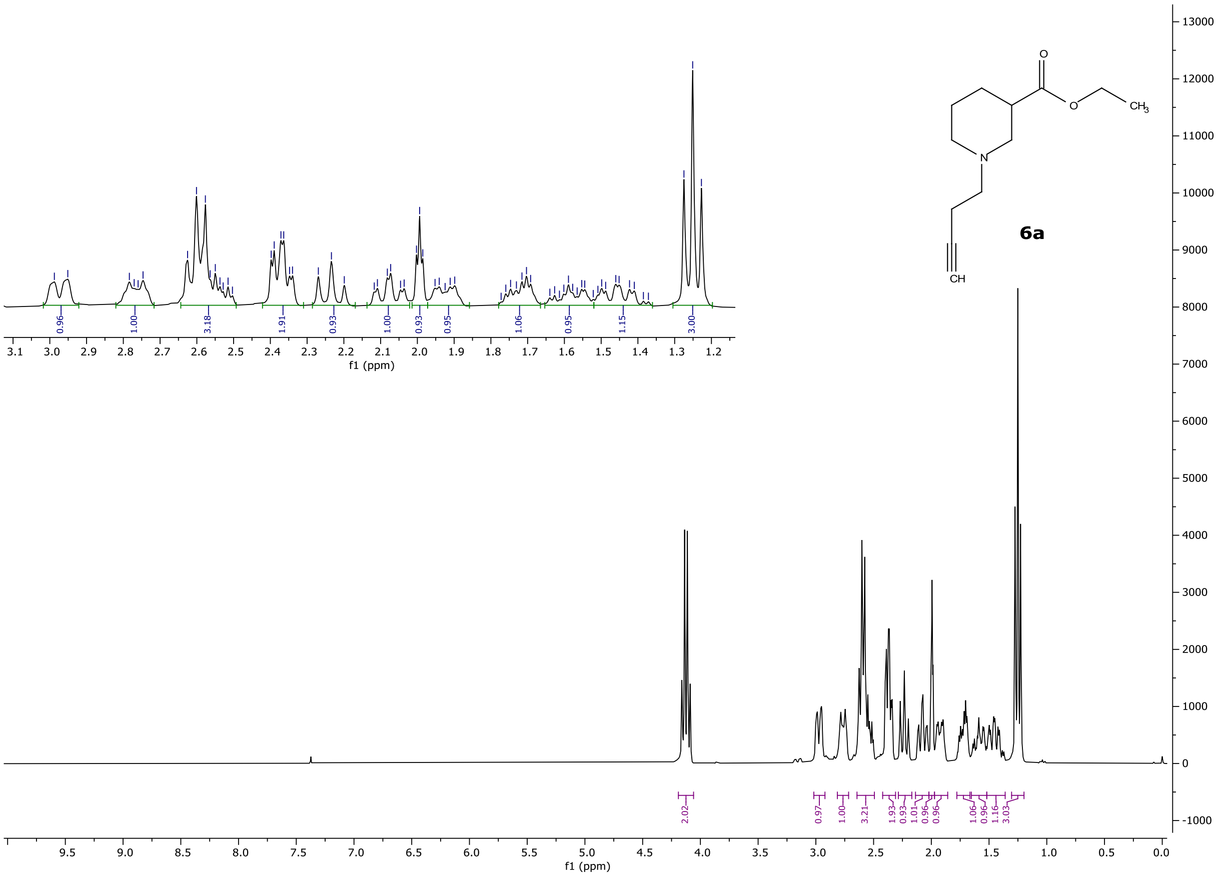

Figure S37: ^{1}H NMR spectrum ($CDCl_3$, 300 MHz) of **6a**.

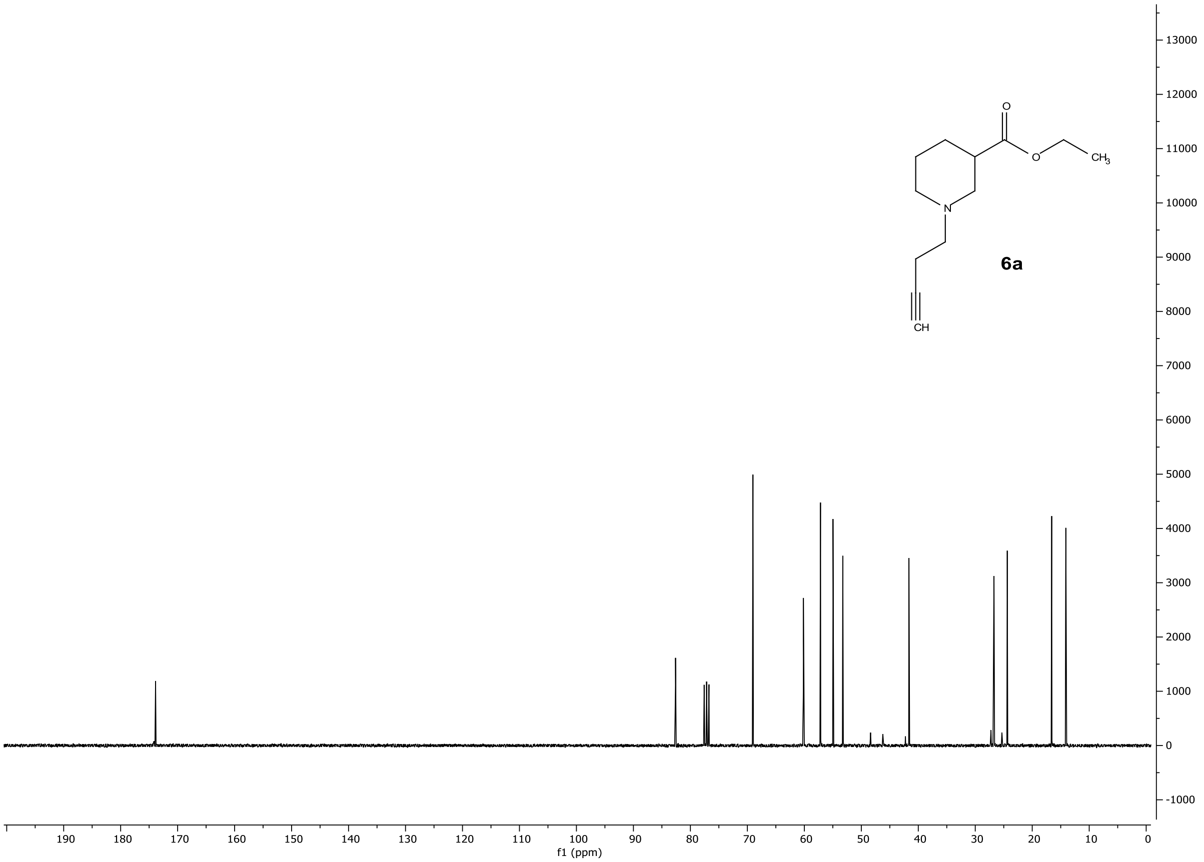

Figure S38: ^{13}C NMR spectrum ($CDCl_3$, 75 MHz) of **6a**.

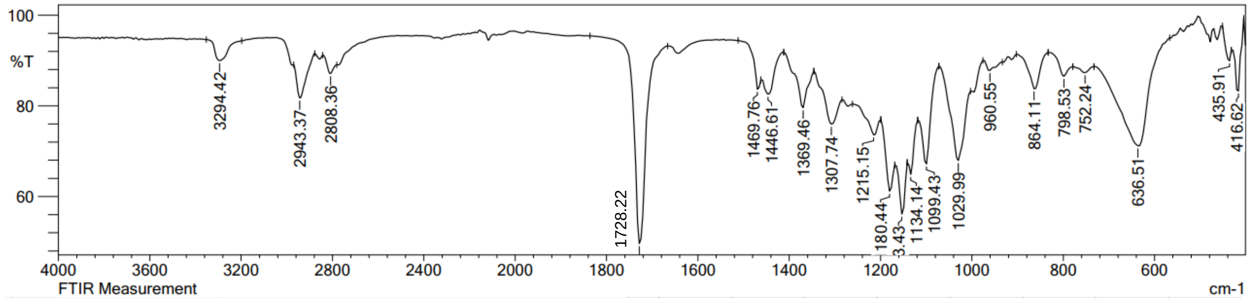

Figure S39: FTIR spectrum of **6a**.

Analytical spectra of **6b**

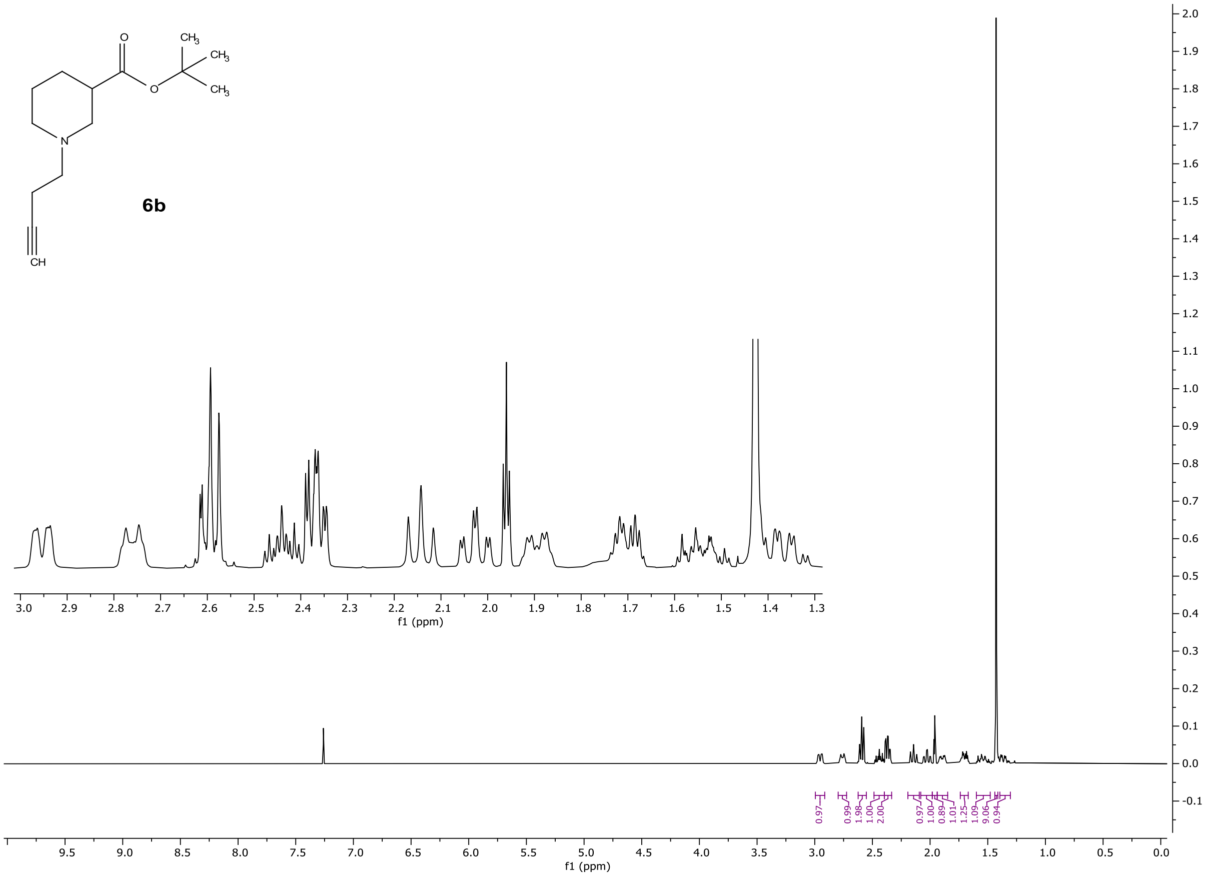

6b

Figure S40: ^{1}H NMR spectrum ($CDCl_3$, 400 MHz) of **6b**.

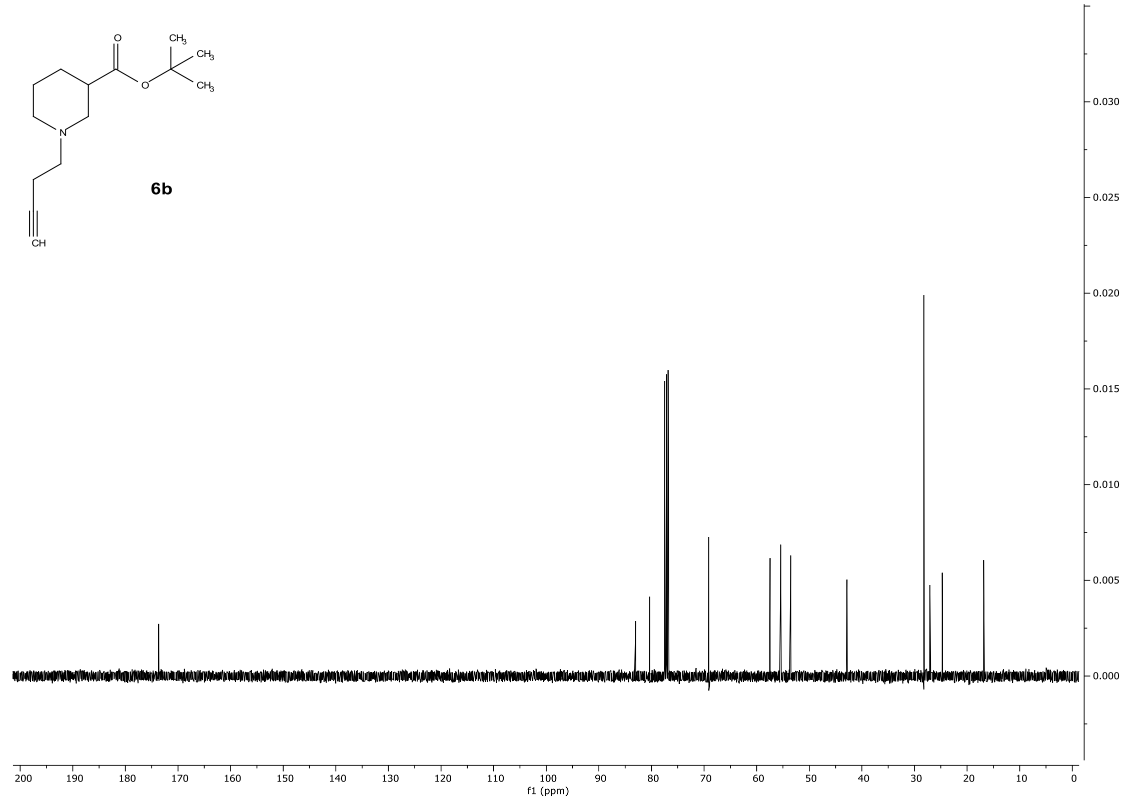

Figure S41: ^{13}C NMR spectrum ($CDCl_3$, 101 MHz) of **6b**.

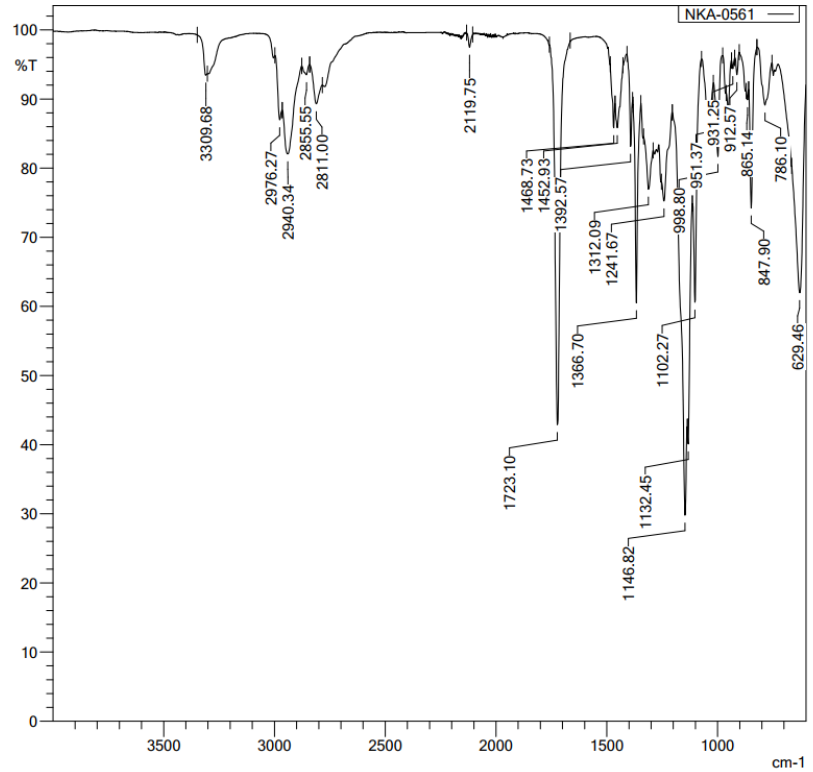

Figure S42: FTIR spectrum of **6b**.

Analytical spectra of **7a**

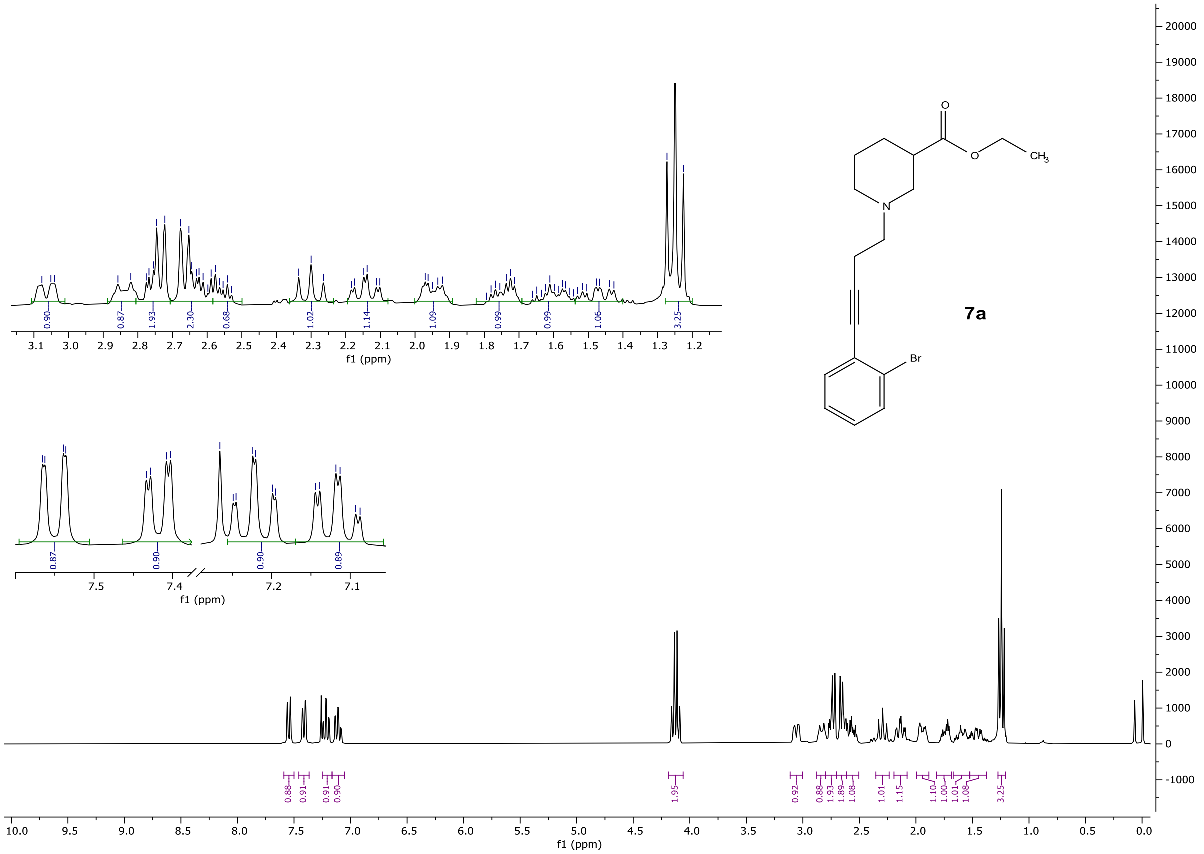

Figure S43: ^{1}H NMR spectrum ($CDCl_3$, 300 MHz) of **7a**.

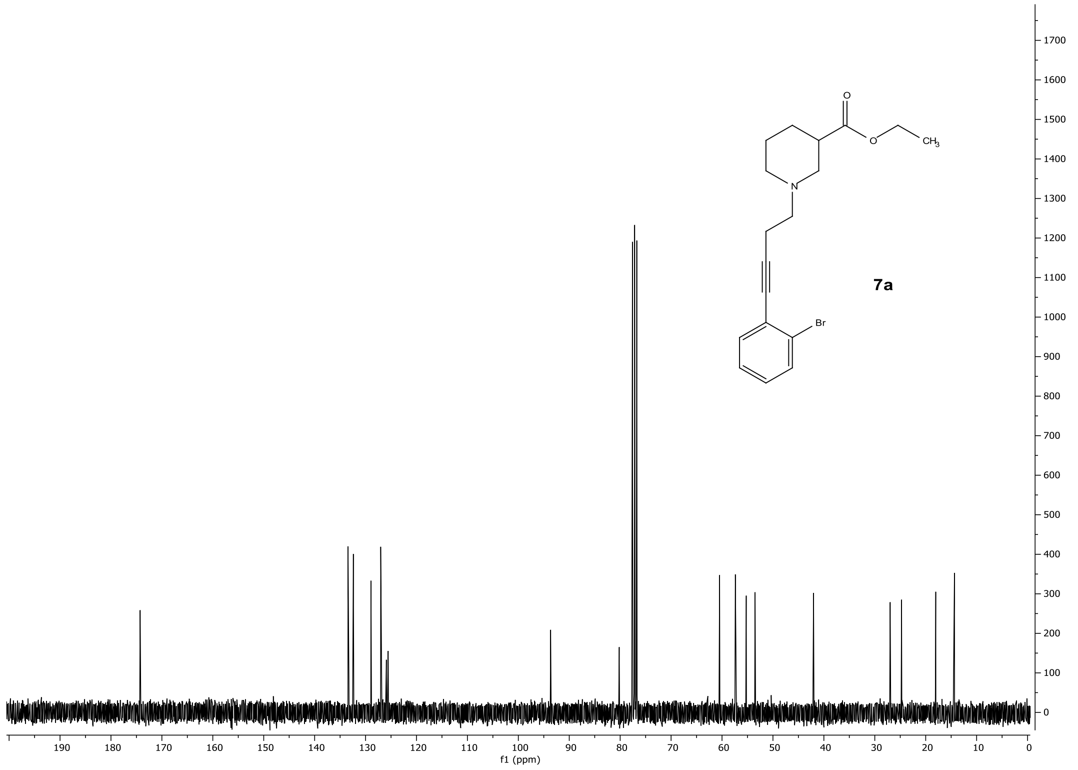

Figure S44: ^{13}C NMR spectrum ($CDCl_3$, 75 MHz) of **7a**.

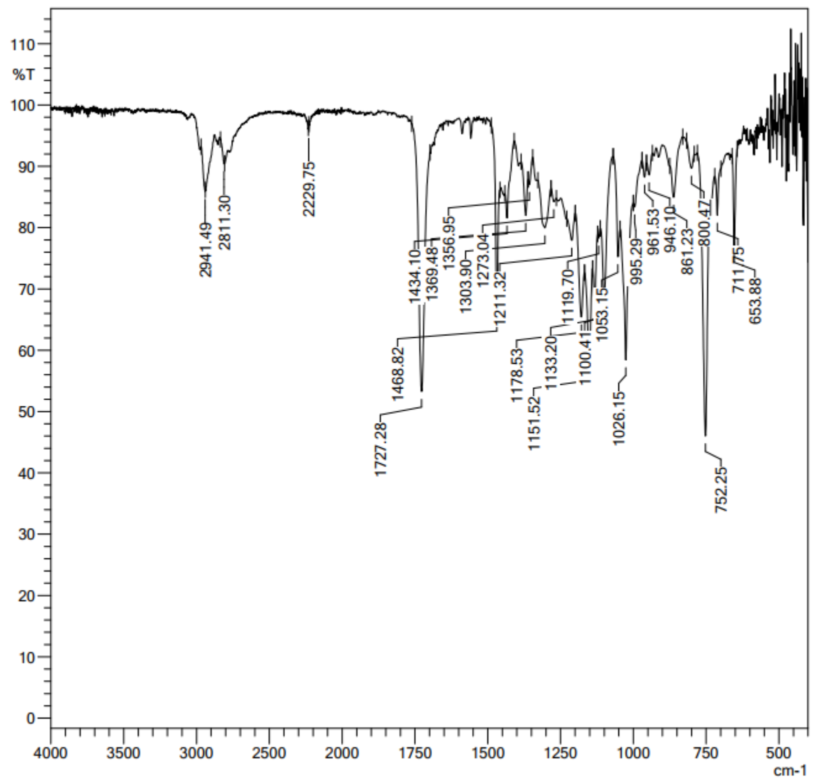

Figure S45: FTIR spectrum of **7a**.

Analytical spectra of **7b**

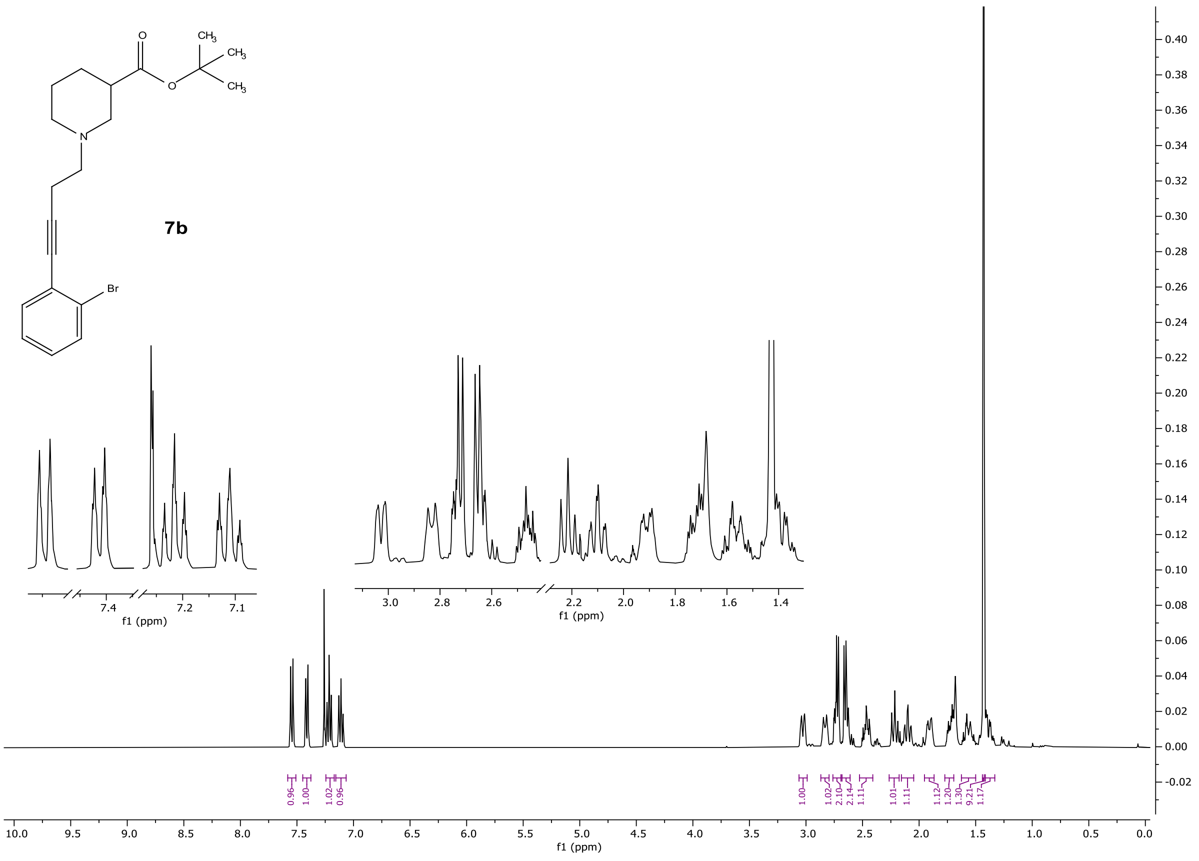

Figure S46: ^{1}H NMR spectrum ($CDCl_3$, 400 MHz) of **7b**.

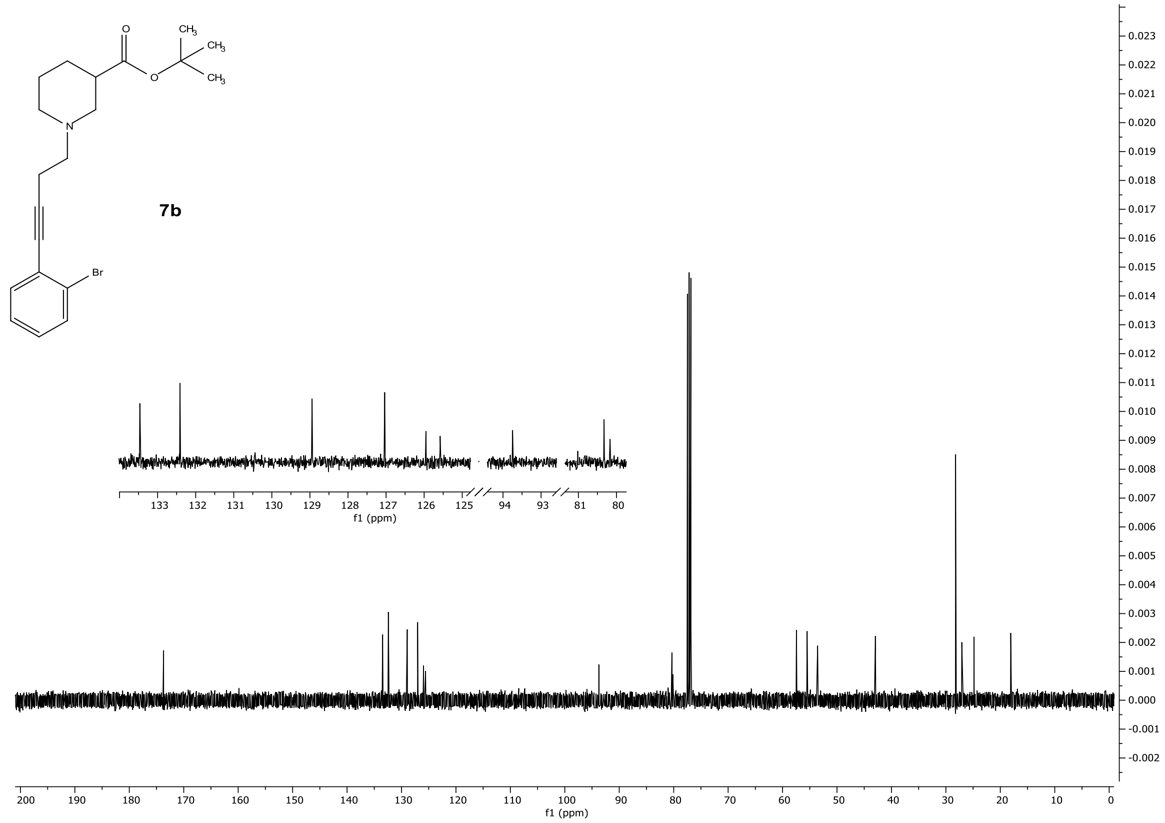

Figure S47: ^{13}C NMR spectrum ($CDCl_3$, 101 MHz) of **7b**.

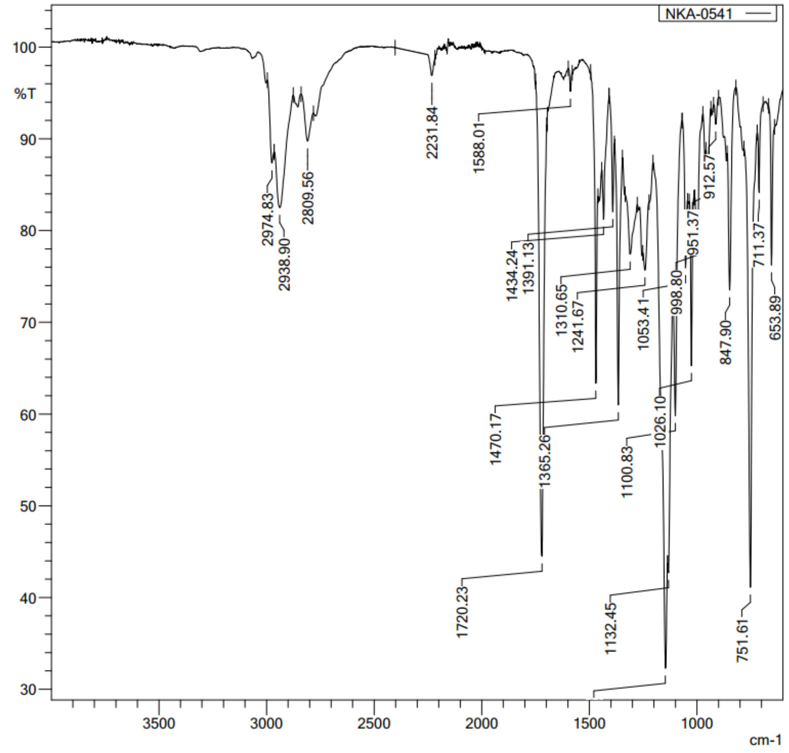

Figure S48: FTIR spectrum of **7b**.

Analytical spectra of **8a**

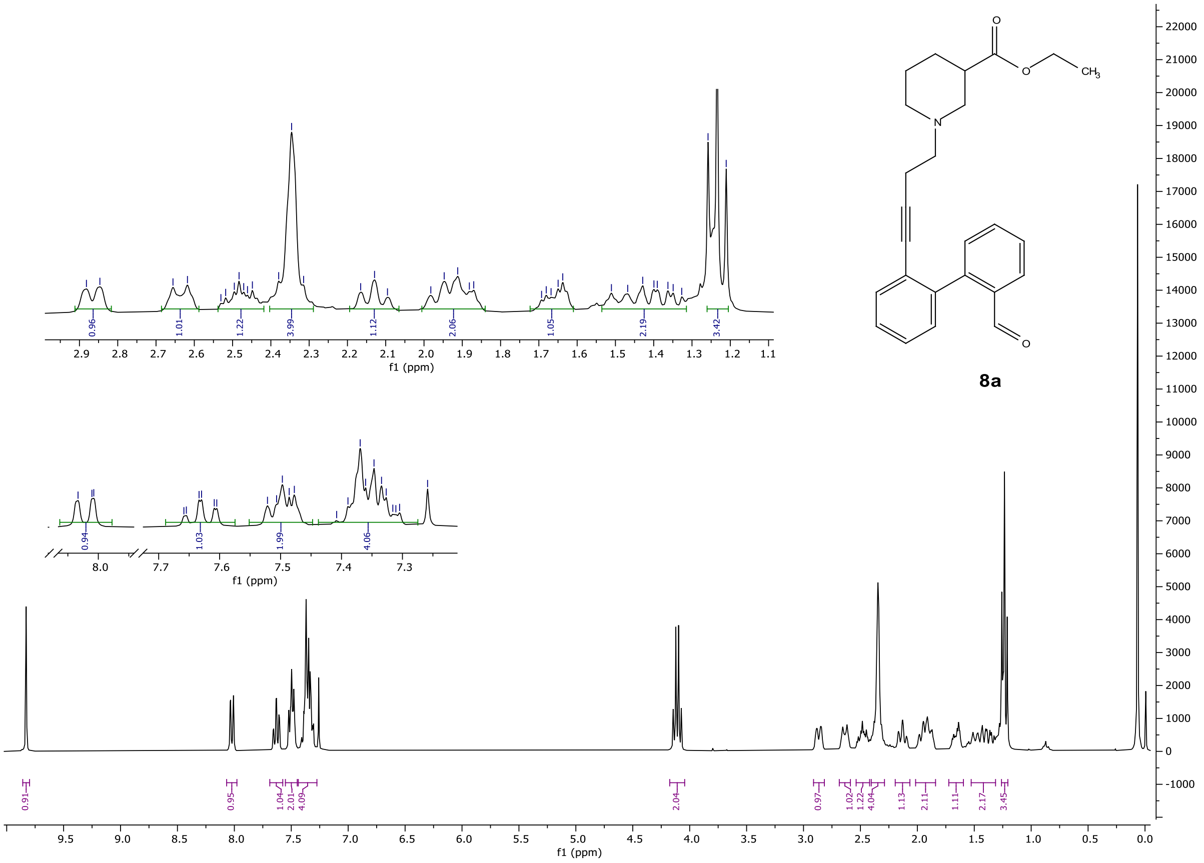

Figure S49: ^{1}H NMR spectrum ($CDCl_3$, 300 MHz) of **8a**.

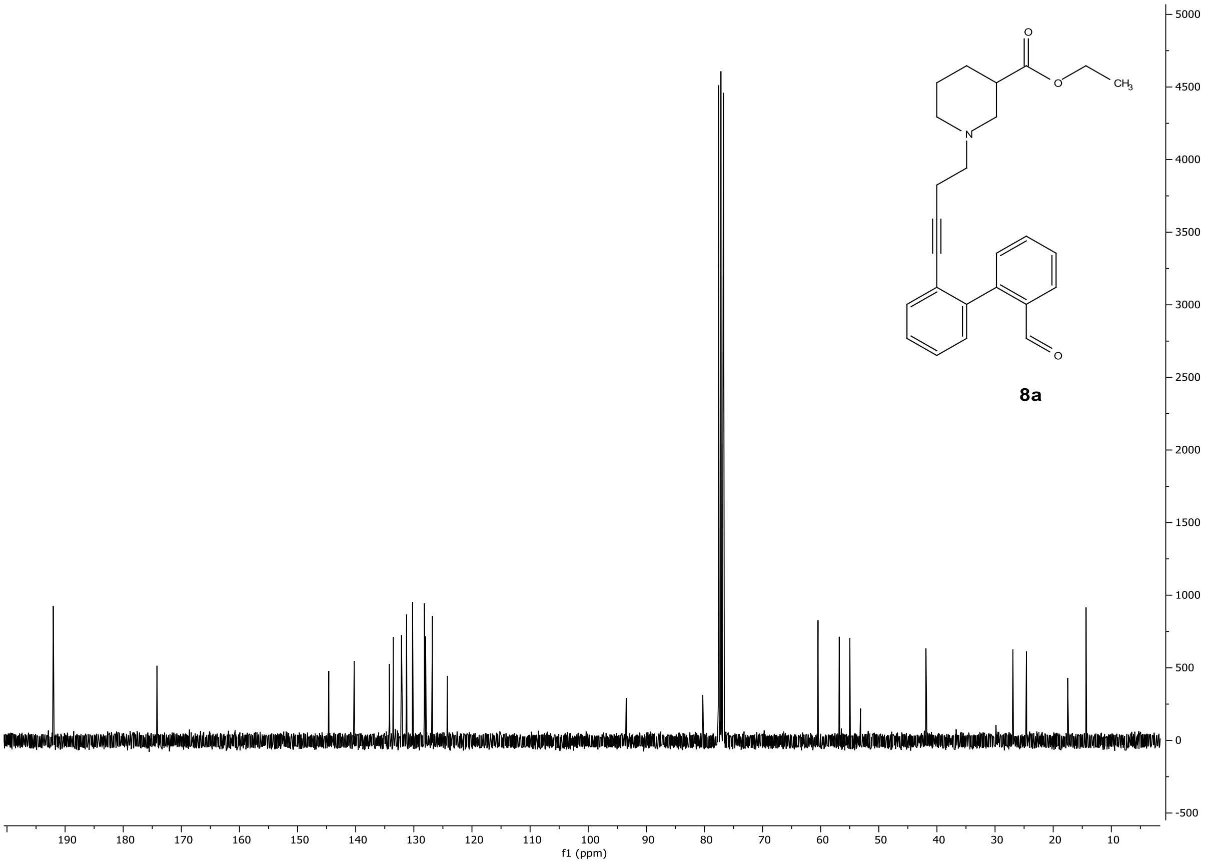

Figure S50: ^{13}C NMR spectrum ($CDCl_3$, 75 MHz) of **8a**.

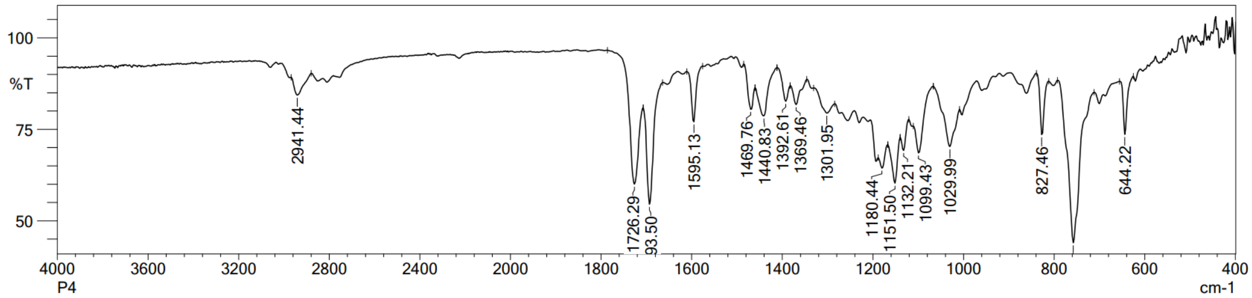

Figure S51: FTIR spectrum of **8a**.

Analytical spectra of **8b**

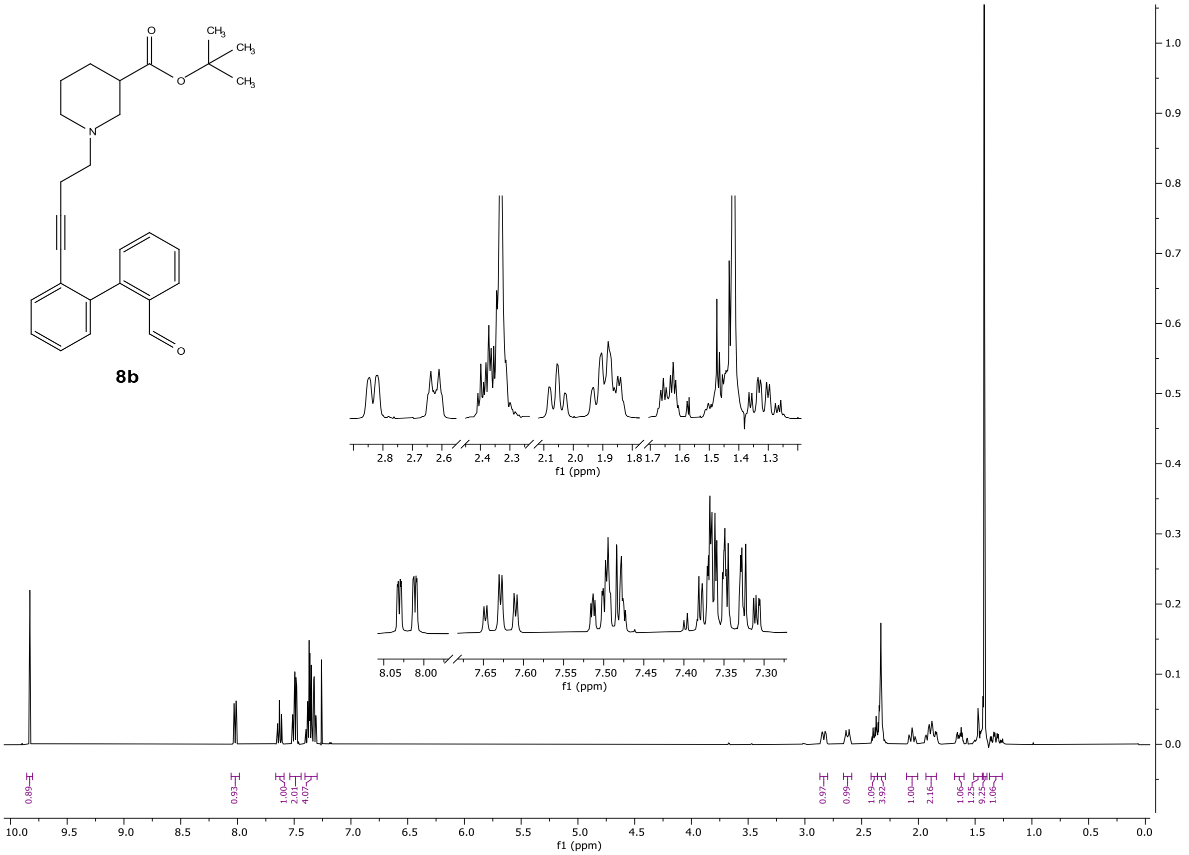

Figure S52: ^{1}H NMR spectrum ($CDCl_3$, 400 MHz) of **8b**.

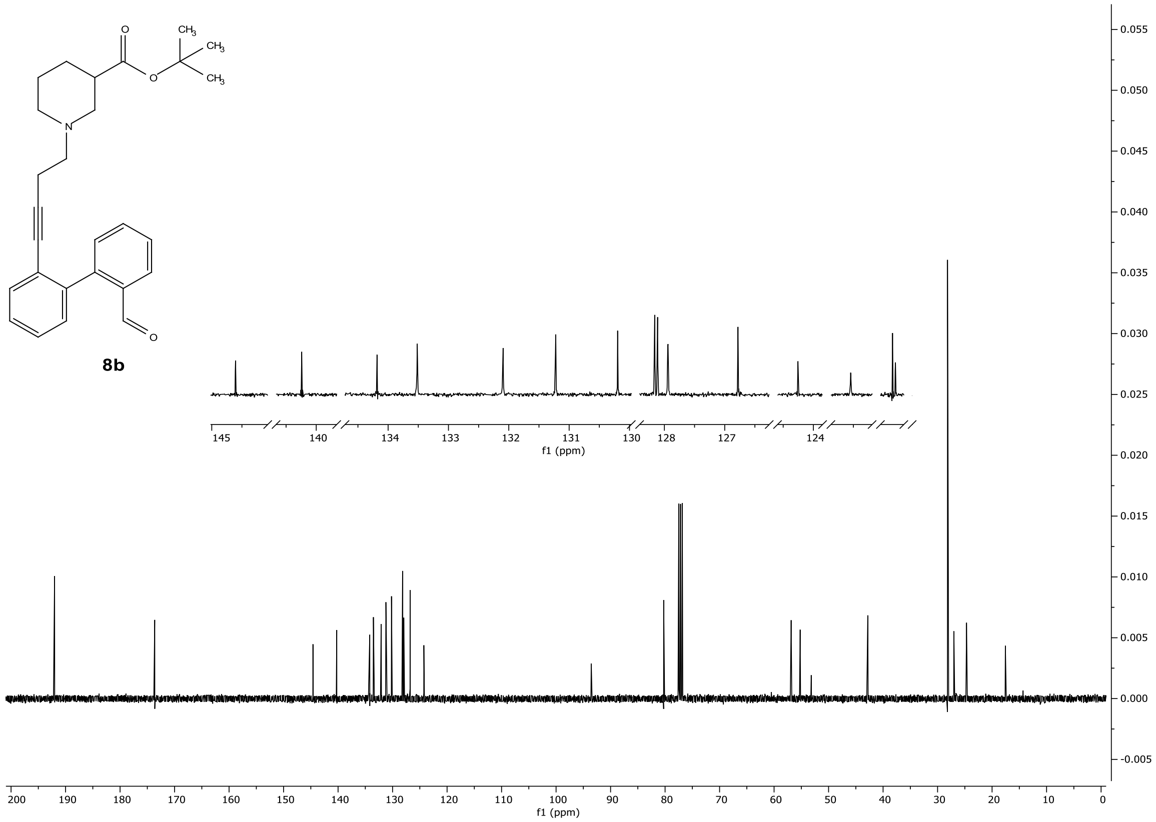

Figure S53: ^{13}C NMR spectrum ($CDCl_3$, 101 MHz) of **8b**.

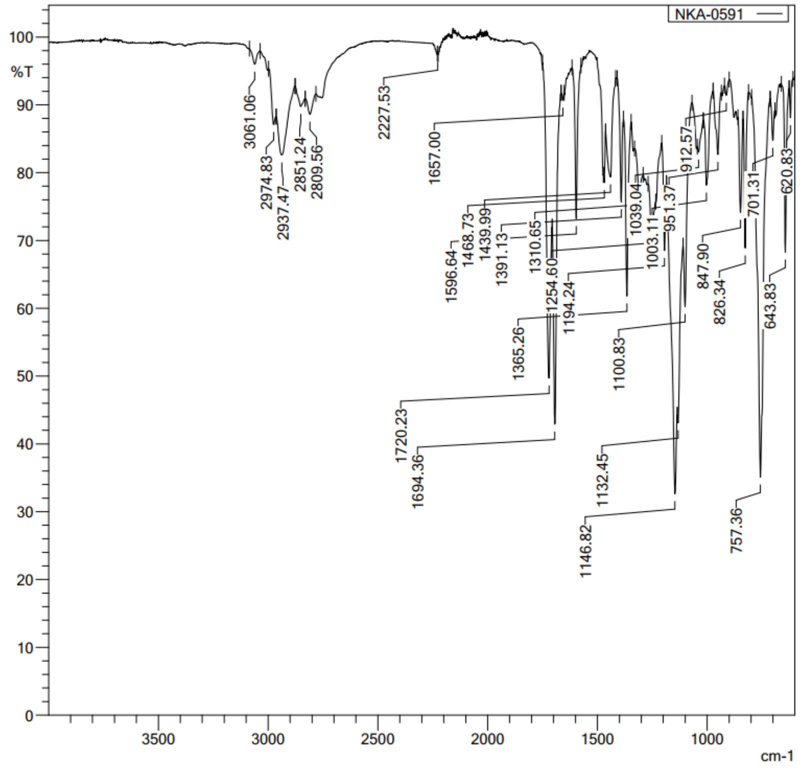

Figure S54: FTIR spectrum of **8b**.

Analytical spectra of **9a**

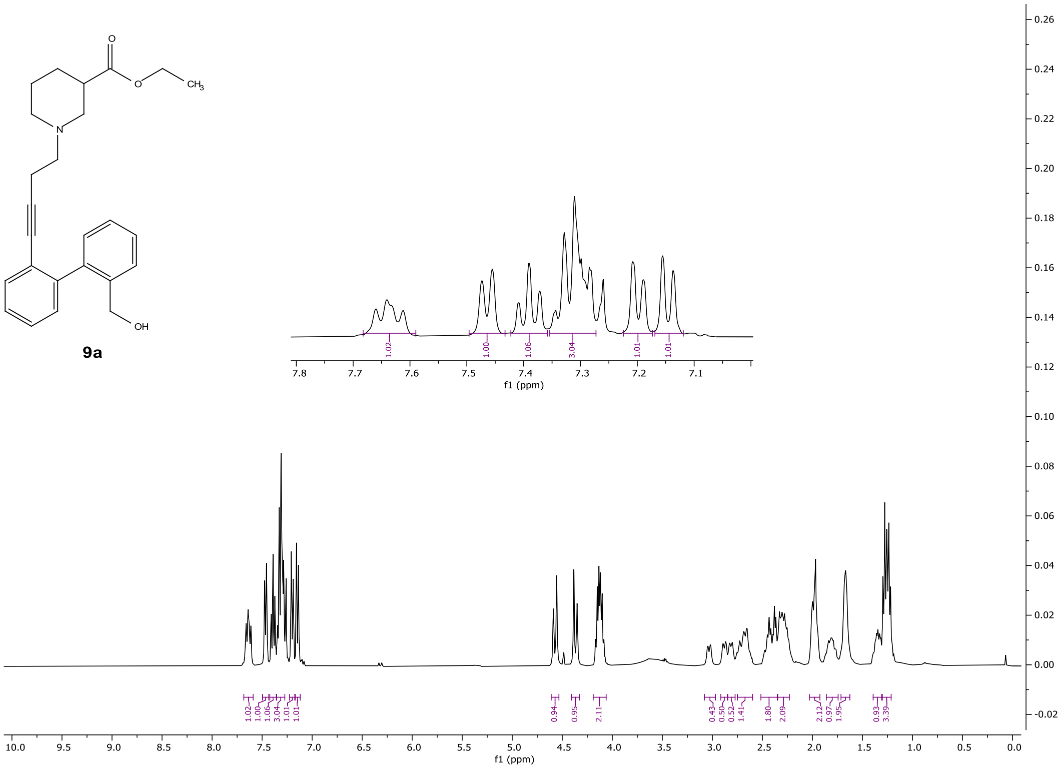

Figure S55: ^{1}H NMR spectrum ($CDCl_3$, 400 MHz) of **9a**.

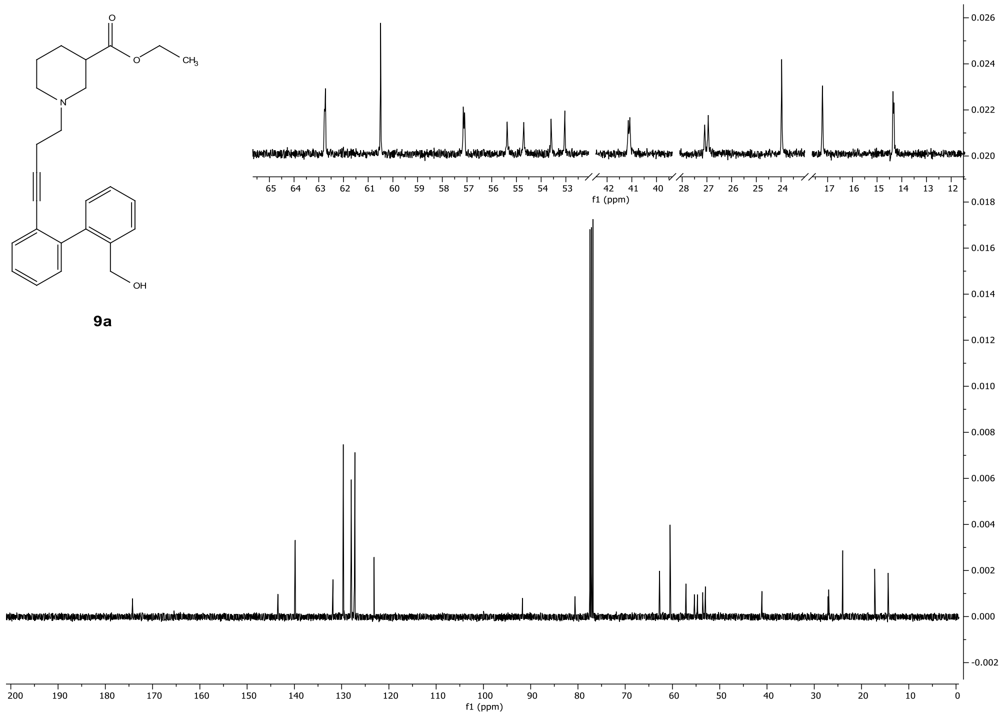

Figure S56: ^{13}C NMR spectrum ($CDCl_3$, 101 MHz) of **9a**.

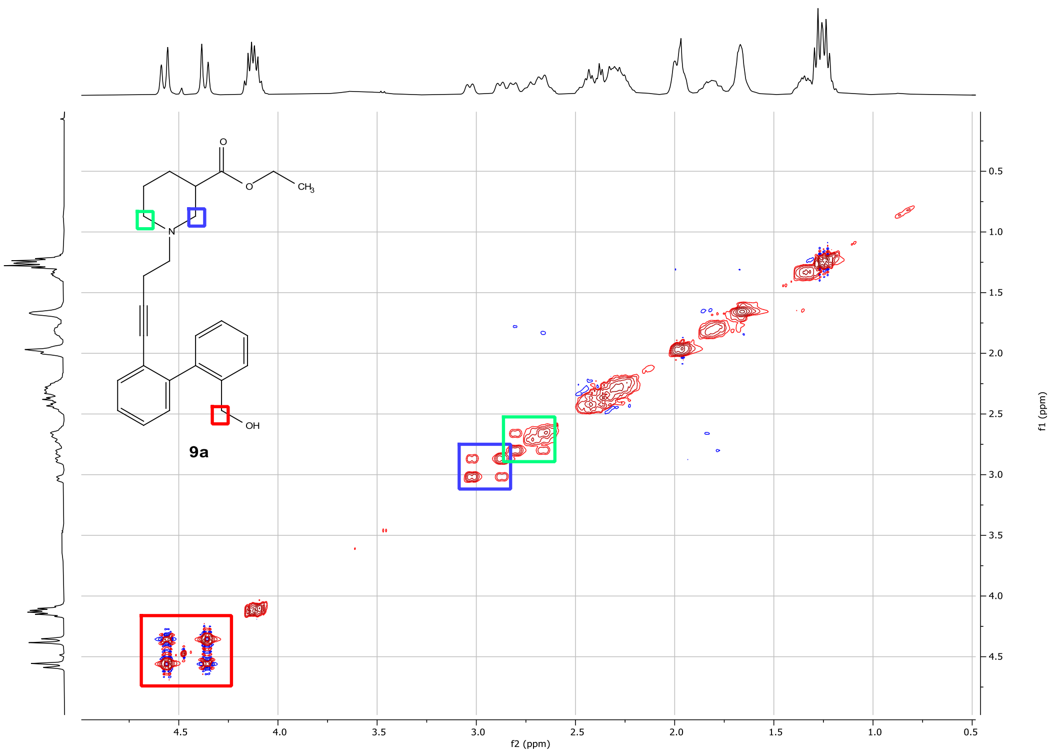

Figure S57: NOESY spectrum of **9a**. Same phase signals for the CH_2OH (red) and the NCH_2 (blue and green) protons show chemical exchange, indicating the presence of intramolecular interactions.[15]

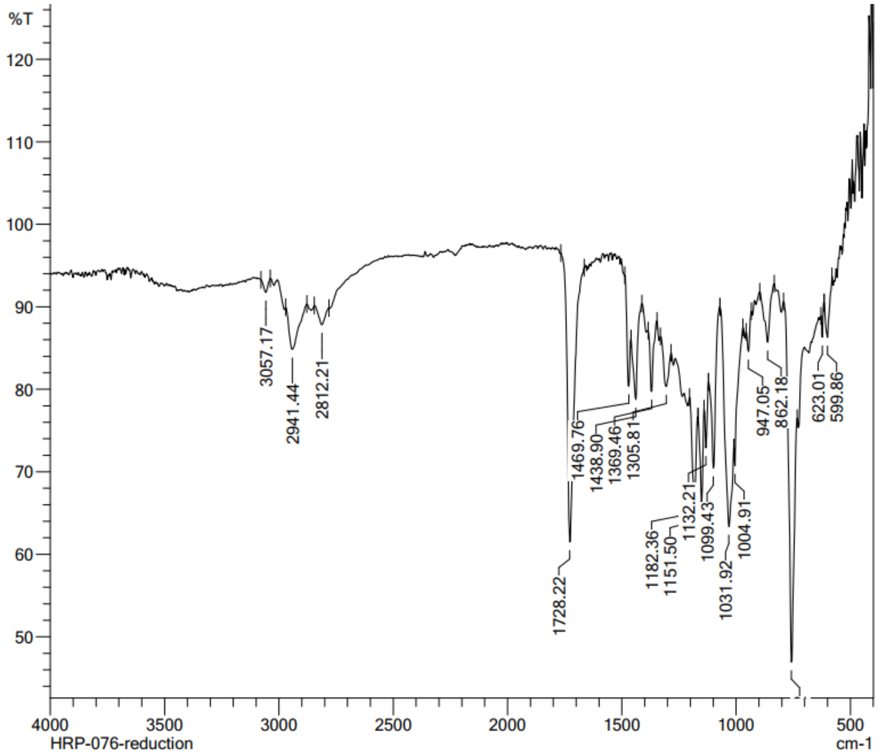

Figure S58: FTIR spectrum of **9a**.

Analytical spectra of **9b**

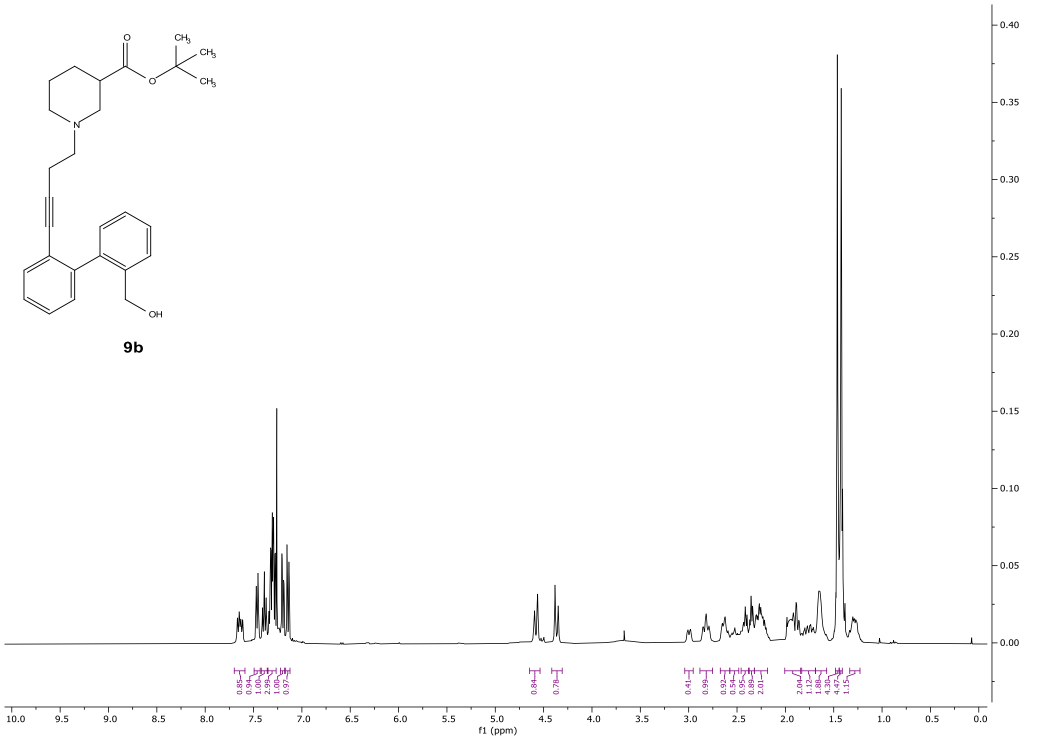

Figure S59: ^{1}H NMR spectrum ($CDCl_3$, 400 MHz) of **9b**.

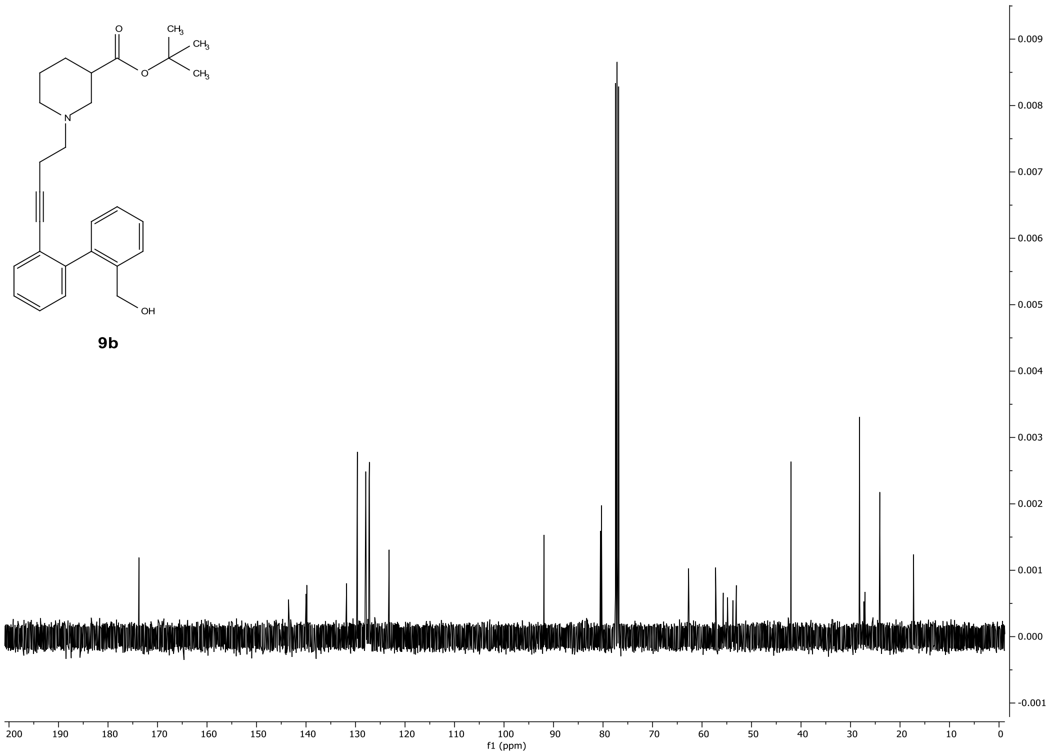

Figure S60: ^{13}C NMR spectrum ($CDCl_3$, 101 MHz) of **9b**.

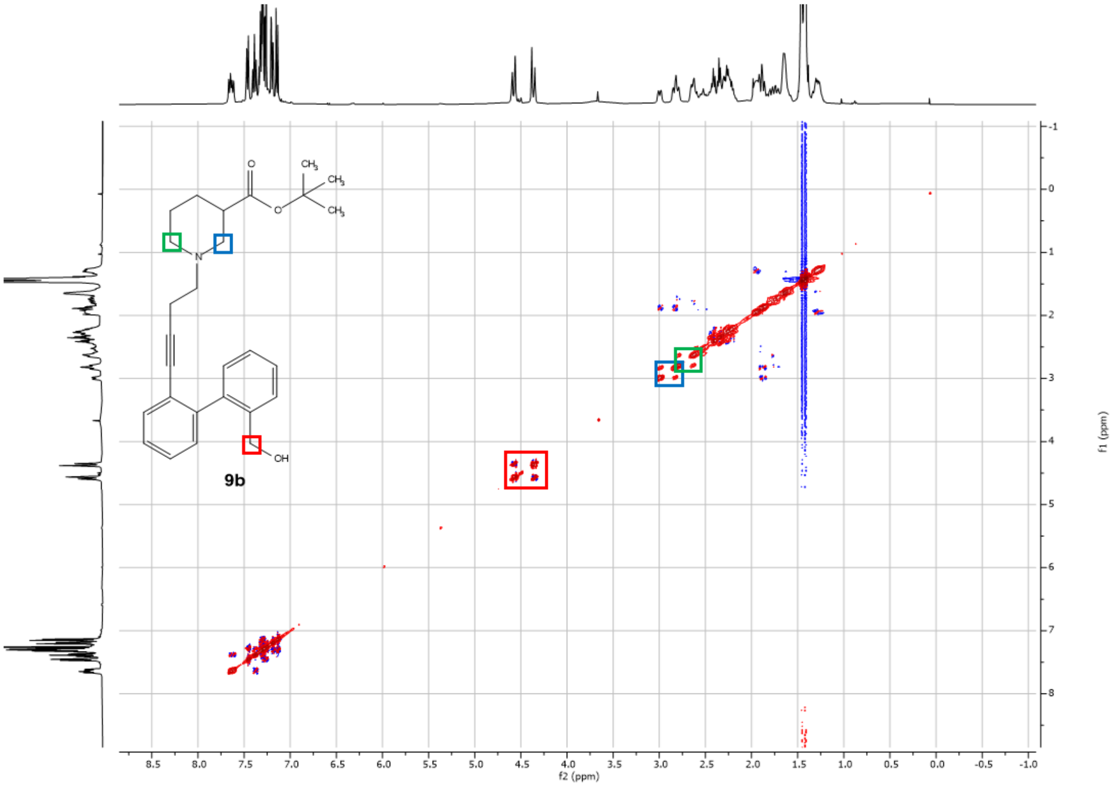

Figure S61: NOESY spectrum of **9b**. Same phase signals for the CH_2OH (red) and the NCH_2 (blue and green) protons show chemical exchange, indicating the presence of intramolecular interactions.[15]

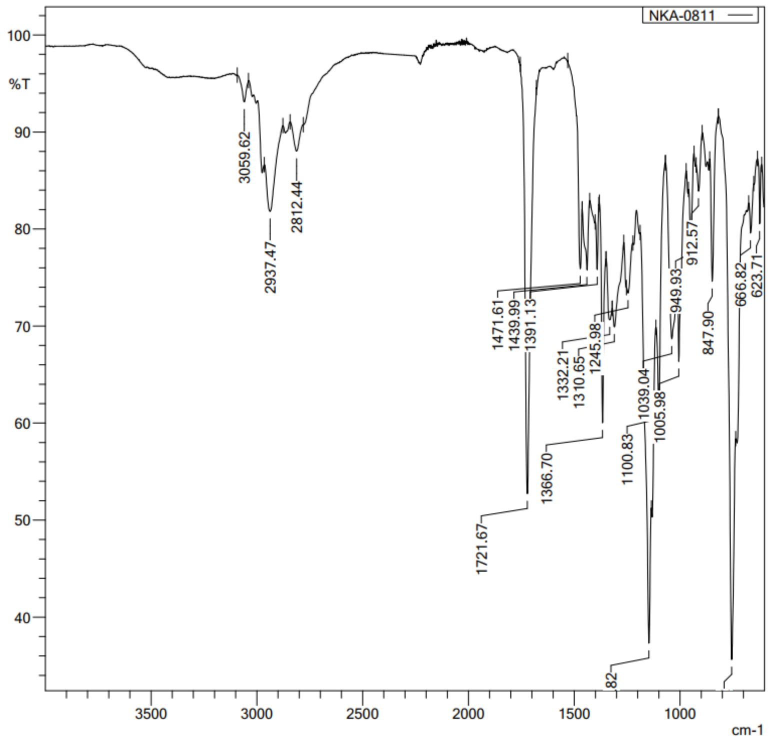

Figure S62: FTIR spectrum of **9b**.

Analytical spectra of **10a**

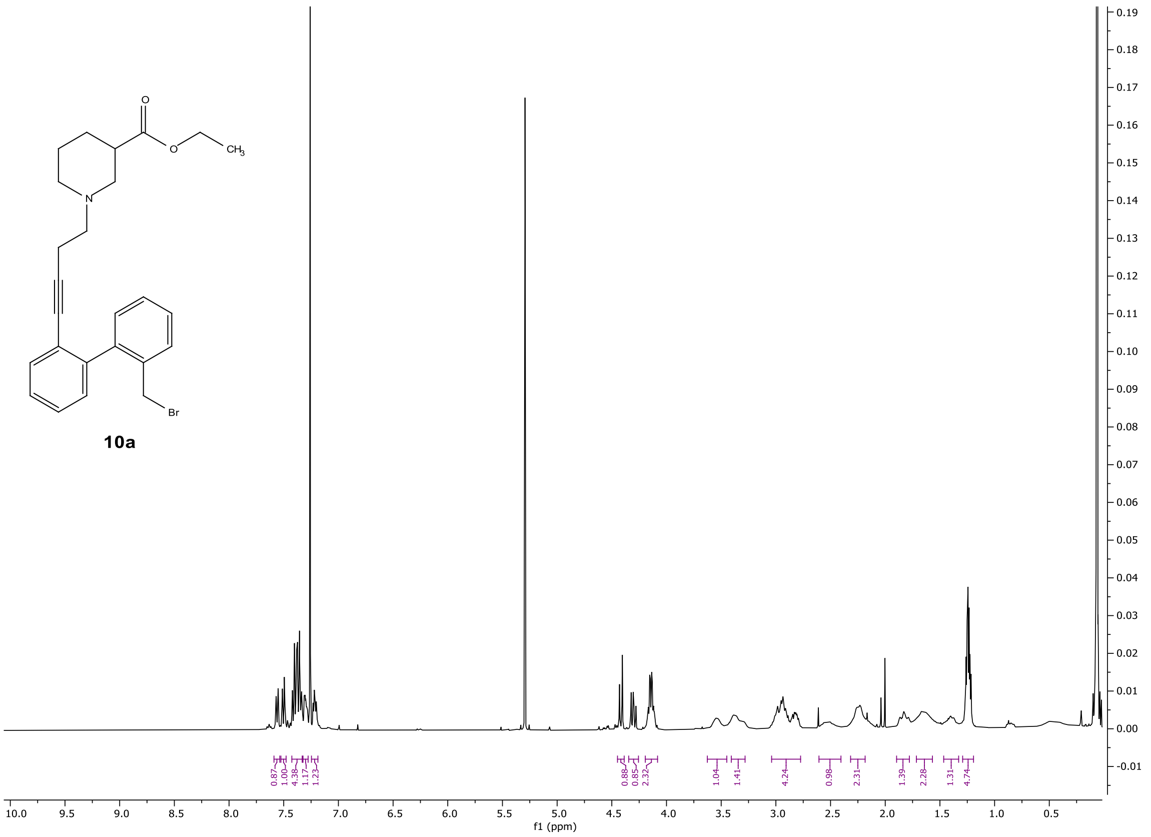

Figure S63: ^{1}H NMR spectrum ($CDCl_3$, 400 MHz) of **10a**. Due to instability of the compound, it was not possible to obtain clean ^{1}H NMR spectra after fully drying the compound. Instead, the obtained ^{1}H NMR directly after purification shows EtOAc and DCM solvent signals mixed with the product **10a**.

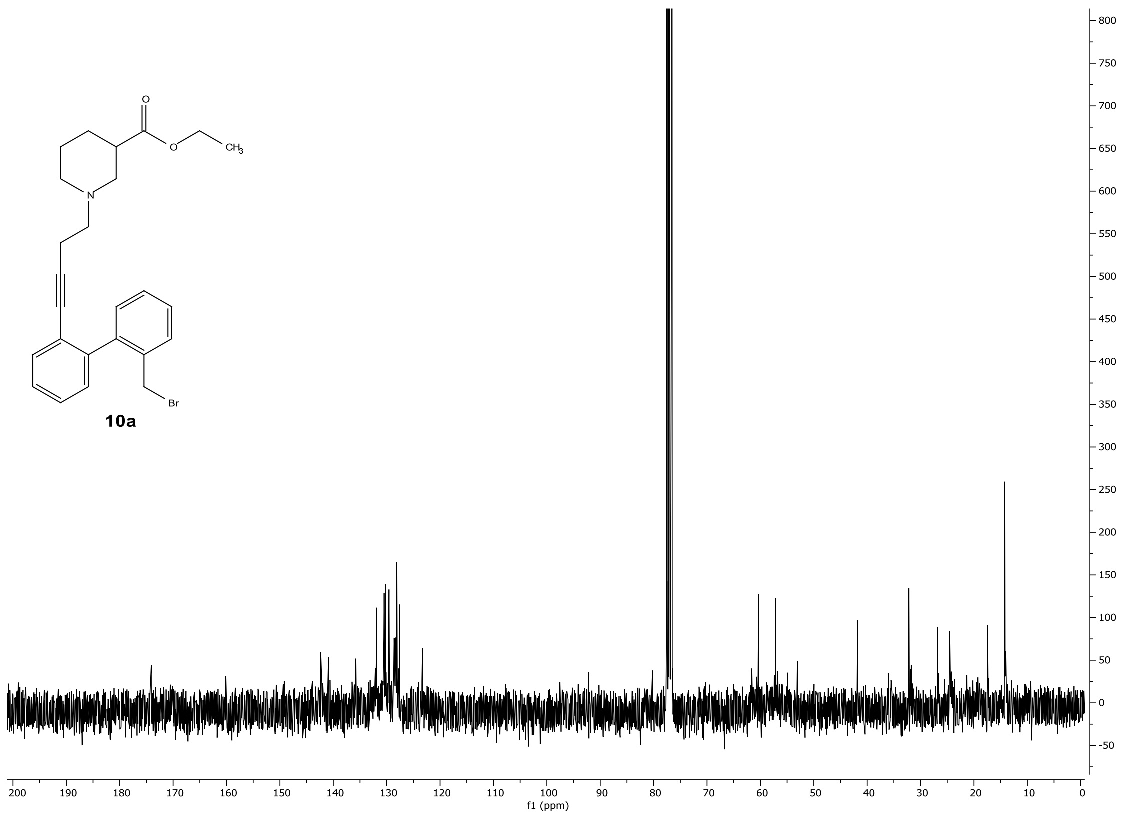

Figure S64: ^{13}C NMR spectrum ($CDCl_3$, 75 MHz) of **10a**.

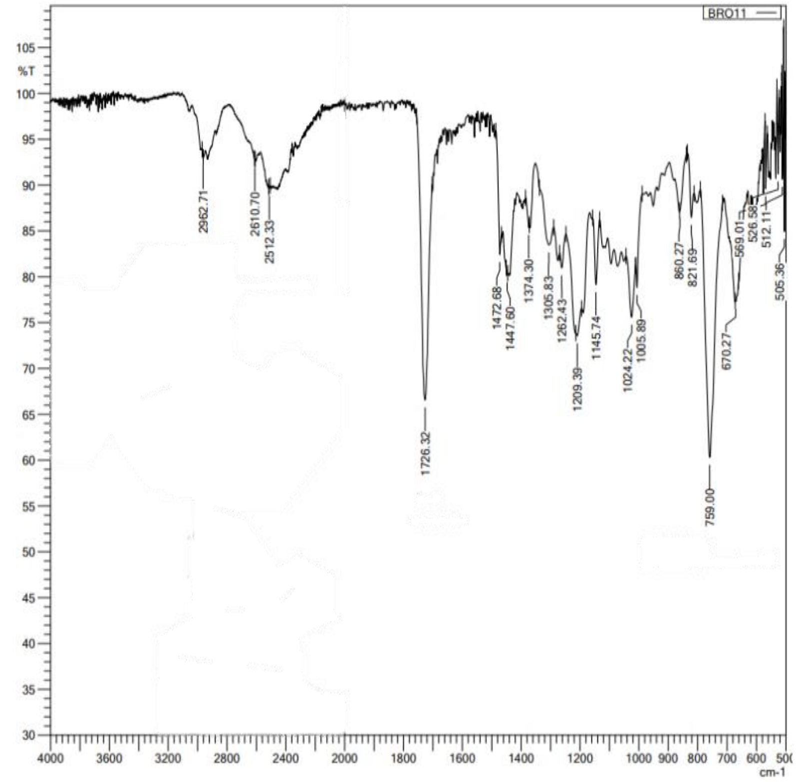

Figure S65: FTIR spectrum of **10a**.

Analytical spectra of **10b**

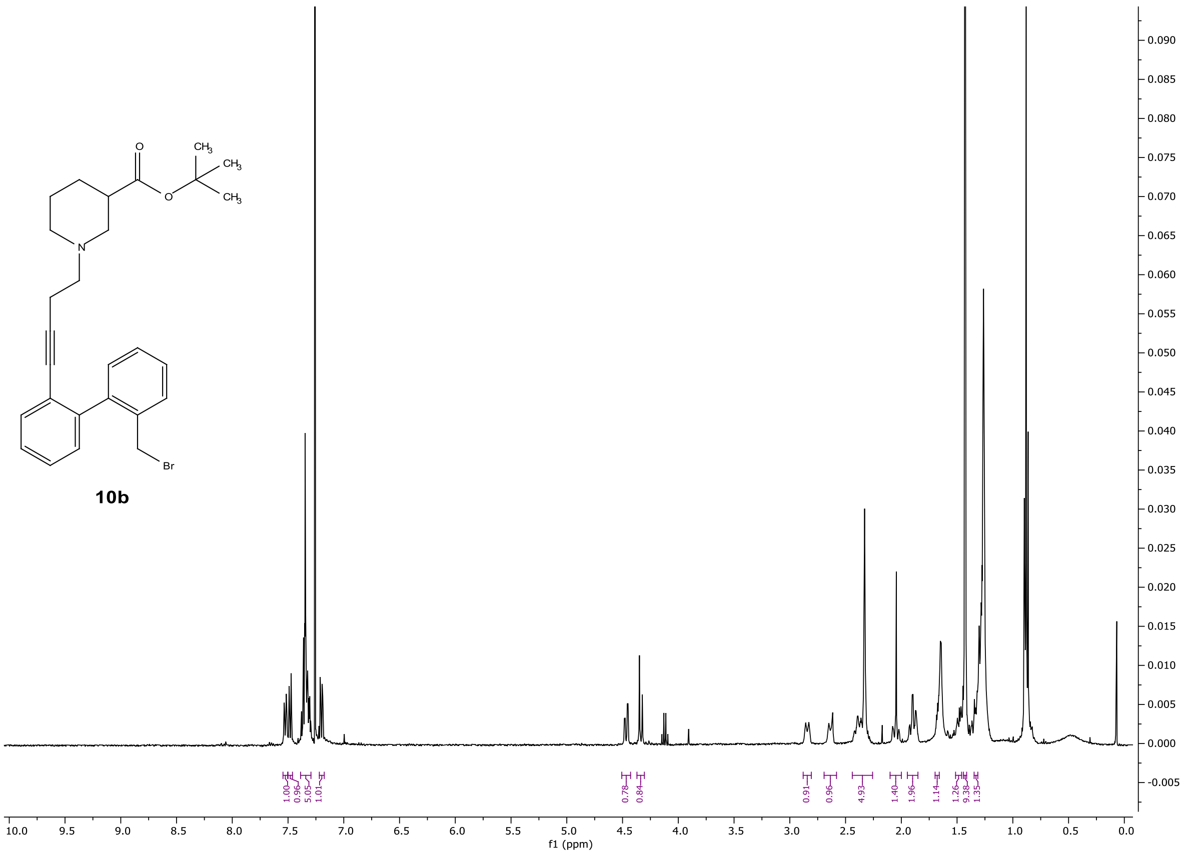

Figure S66: ^{1}H NMR spectrum ($CDCl_3$, 400 MHz) of **10b**. Due to instability of the compound, it was not possible to obtain clean ^{1}H NMR spectra after fully drying the compound. Instead, the obtained ^{1}H NMR directly after purification shows EtOAc and heptane solvent signals mixed with the product **10b**.

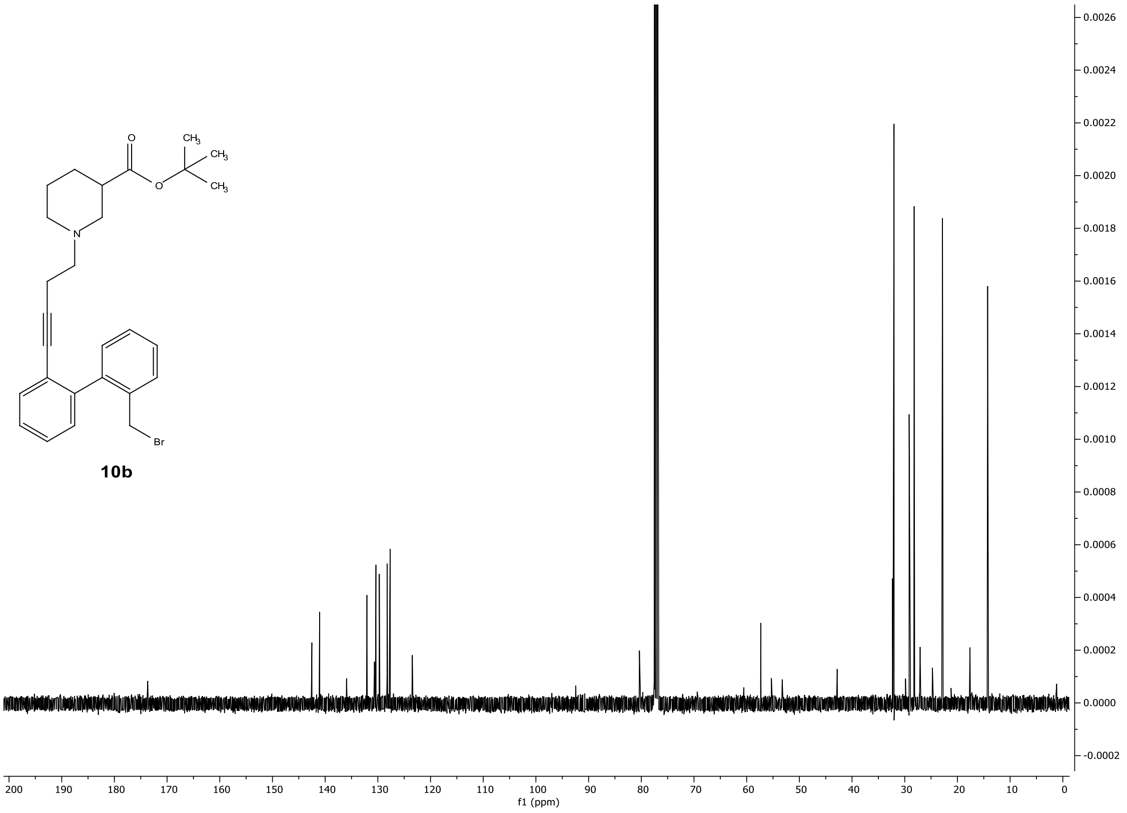

Figure S67: ^{13}C NMR spectrum ($CDCl_3$, 101 MHz) of **10b**. Due to instability of the compound, it was not possible to obtain clean ^{1}H NMR spectra after fully drying the compound. Instead, the obtained ^{1}H NMR directly after purification shows EtOAc and heptane solvent signals mixed with the product **10b**.

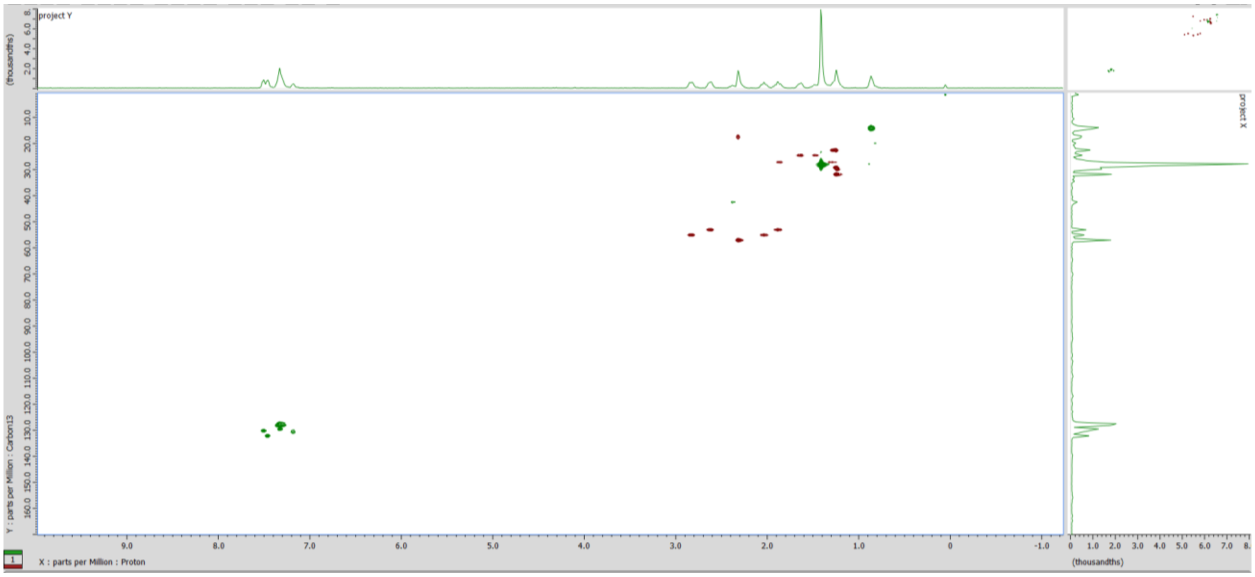

Figure S68: HSQC spectrum of **10b**. Due to instability of the compound, it was not possible to obtain clean ^{1}H NMR spectra after fully drying the compound. Instead, the obtained ^{1}H NMR directly after purification shows EtOAc and heptane solvent signals mixed with the product **10b**.

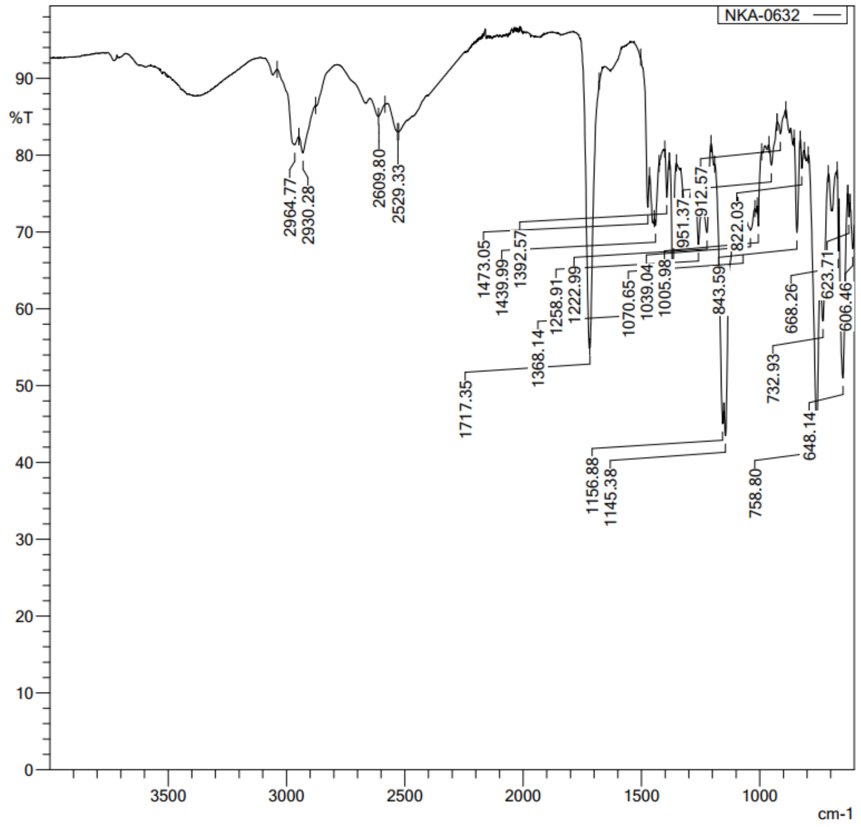

Figure S69: FTIR spectrum of **10b**.

Analytical spectra of **11a**

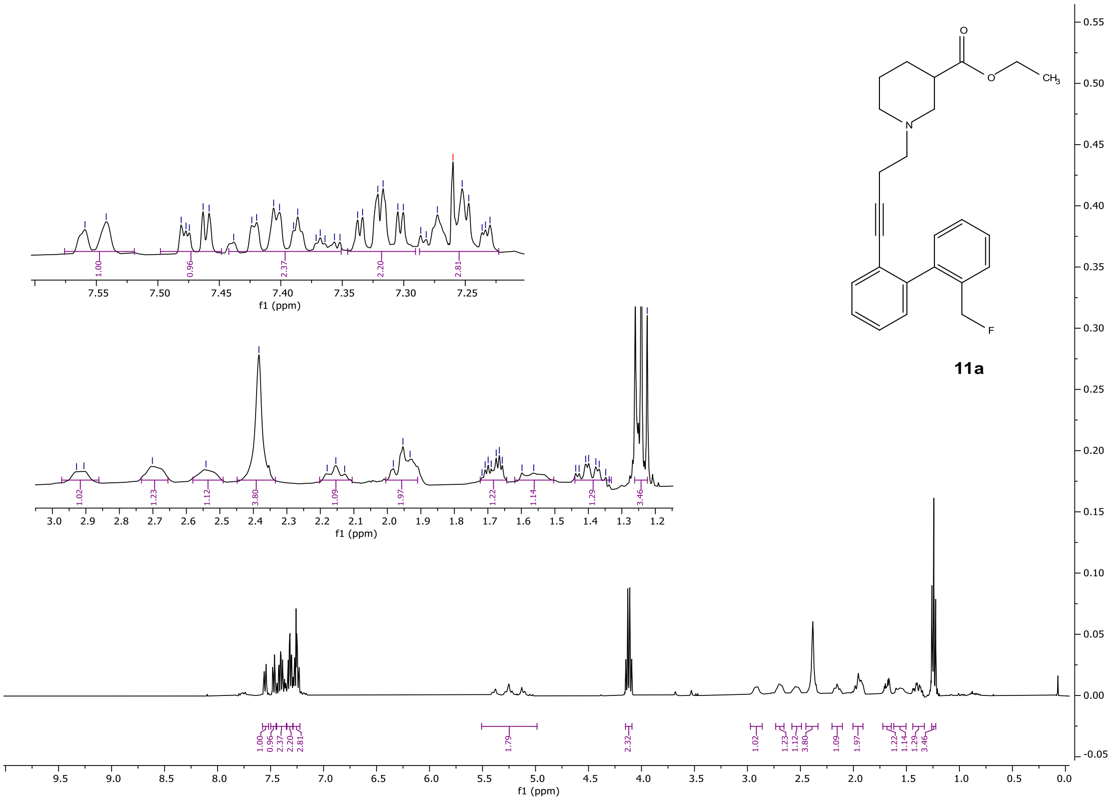

Figure S70: ^{1}H NMR spectrum ($CDCl_3$, 400 MHz) of **11a**.

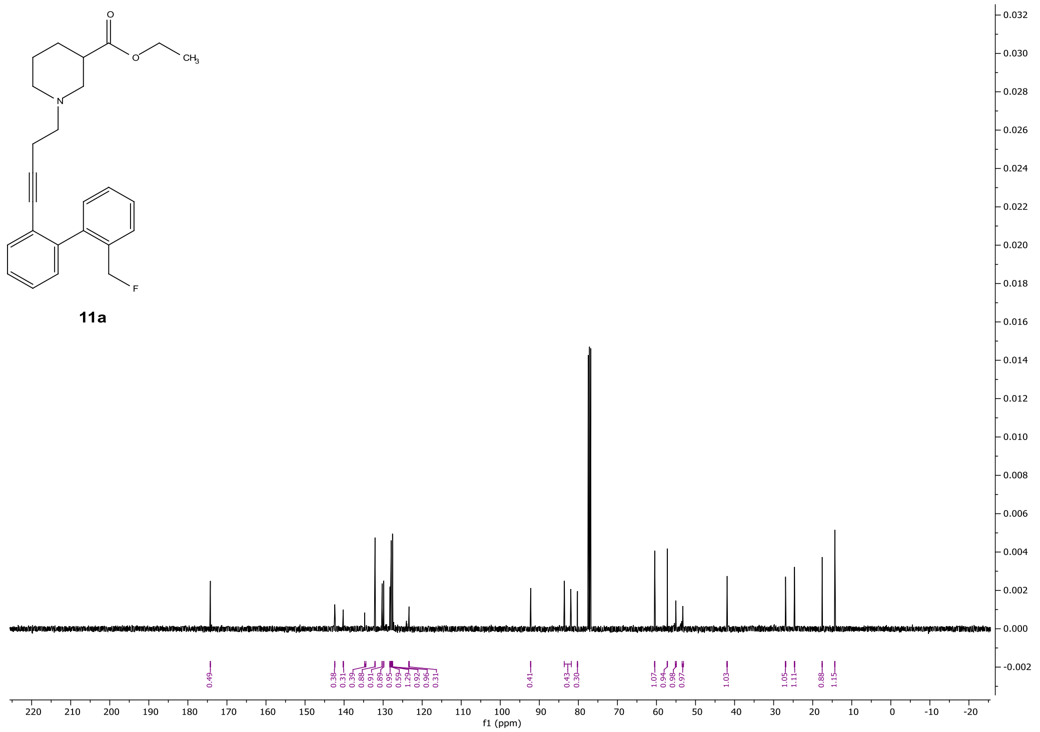

Figure S71: ^{13}C NMR spectrum ($CDCl_3$, 101 MHz) of **11a**.

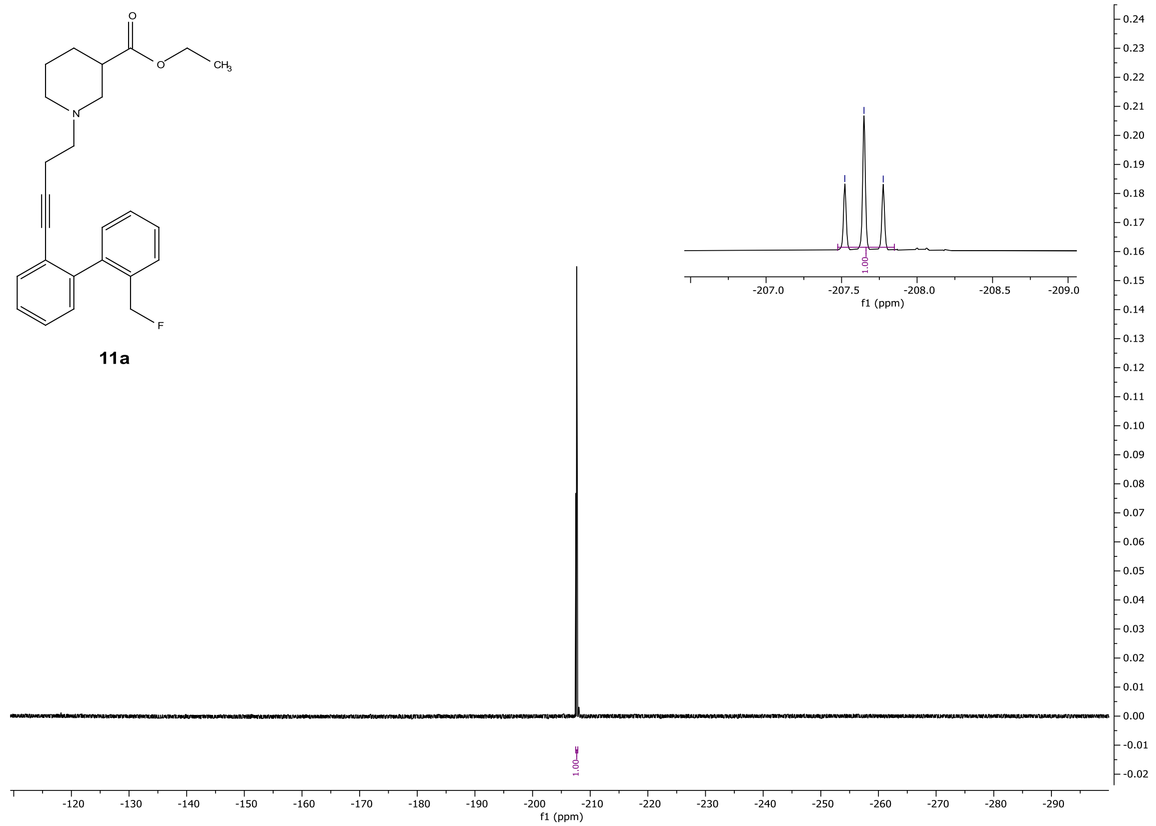

Figure S72: ^{19}F NMR spectrum ($CDCl_3$, 376 MHz) of **11a**.

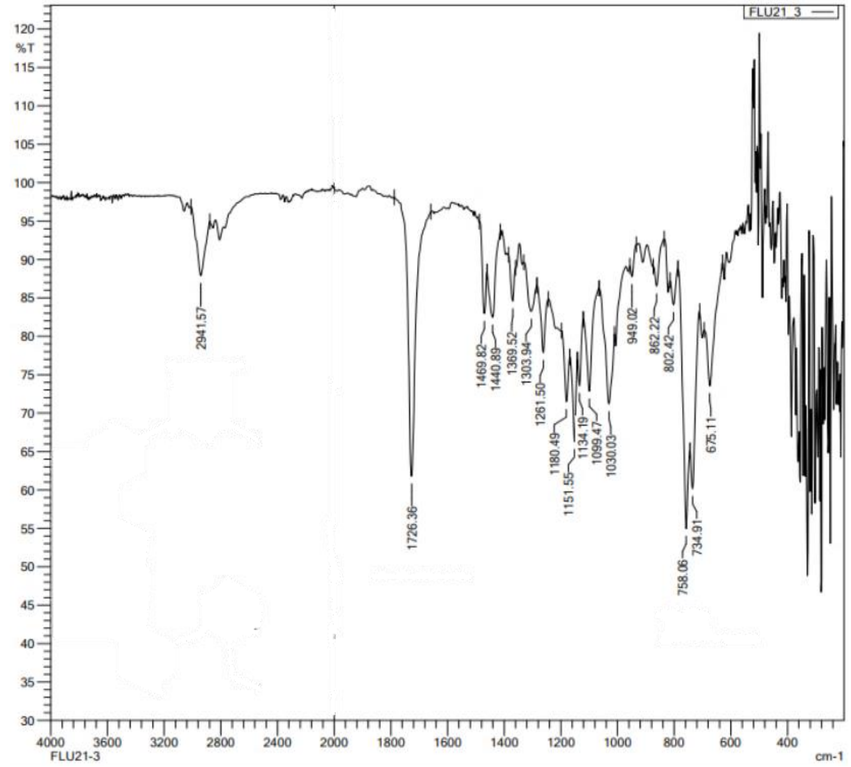

Figure S73: FTIR spectrum of **11a**.

Analytical spectra of **11b**

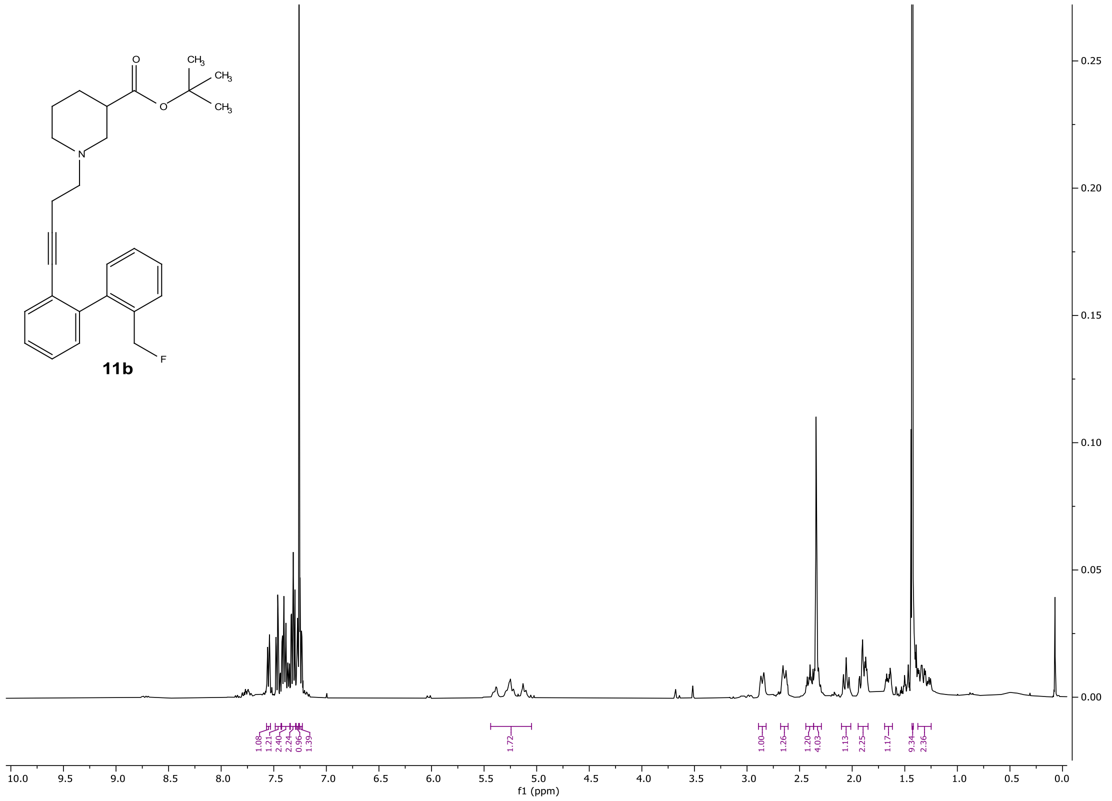

Figure S74: ^{1}H NMR spectrum ($CDCl_3$, 400 MHz) of **11b**.

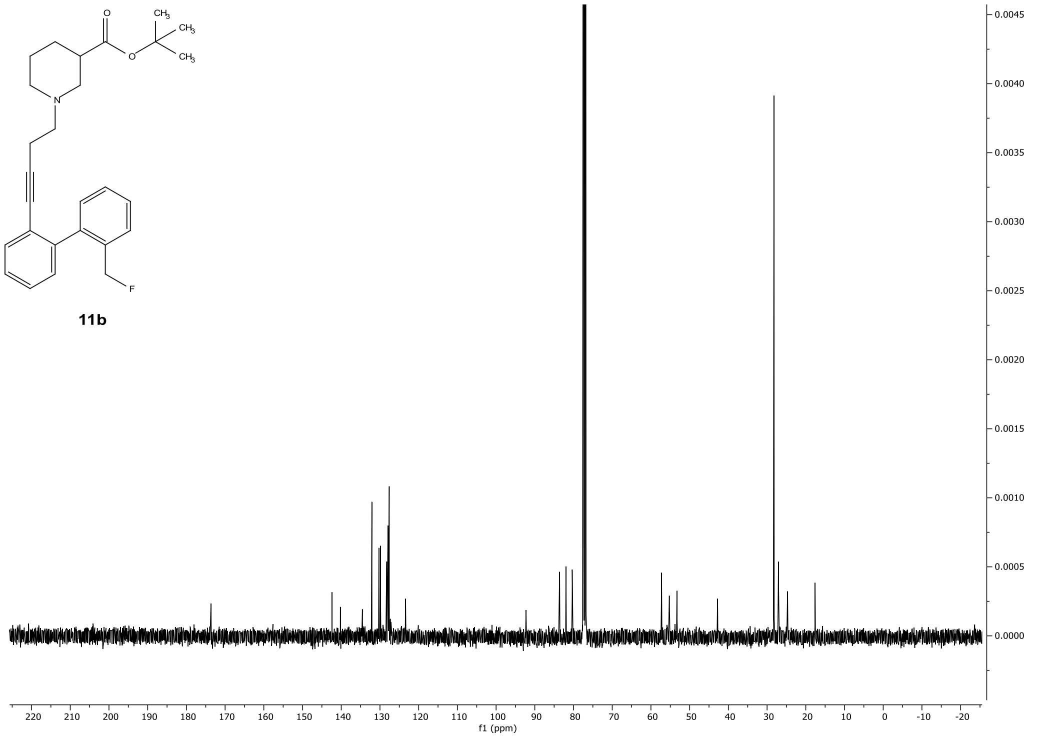

Figure S75: ^{13}C NMR spectrum ($CDCl_3$, 101 MHz) of **11b**.

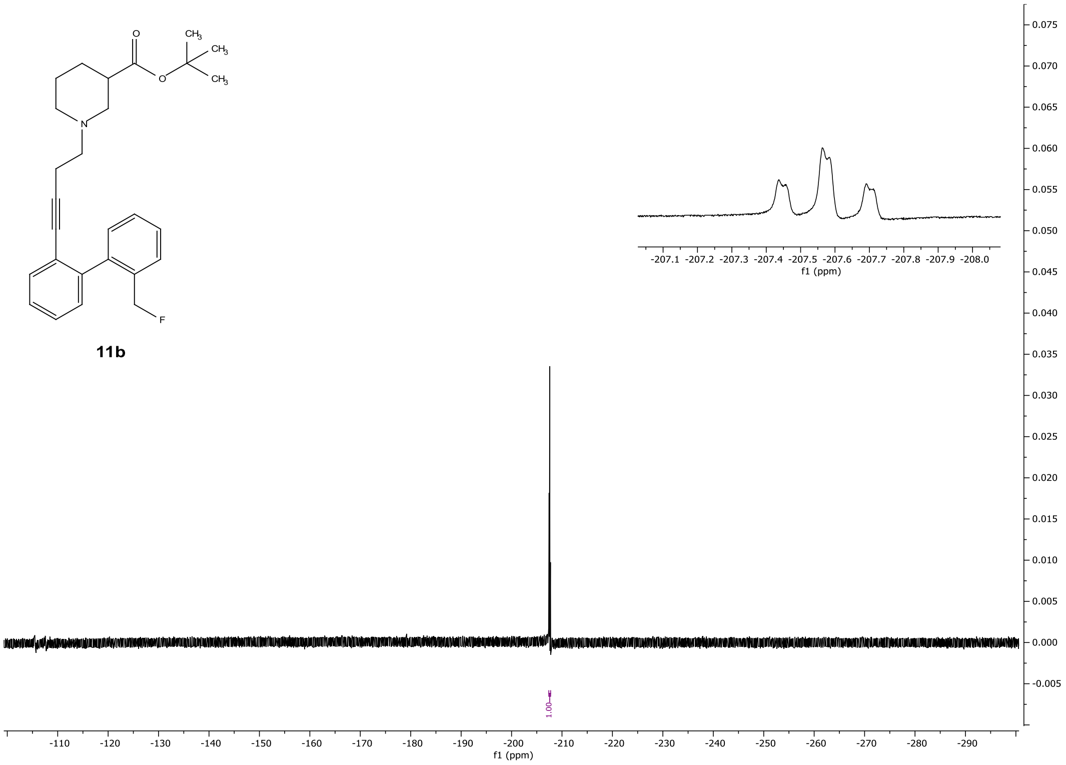

Figure S76: ^{19}F NMR spectrum ($CDCl_3$, 376 MHz) of **11b**.

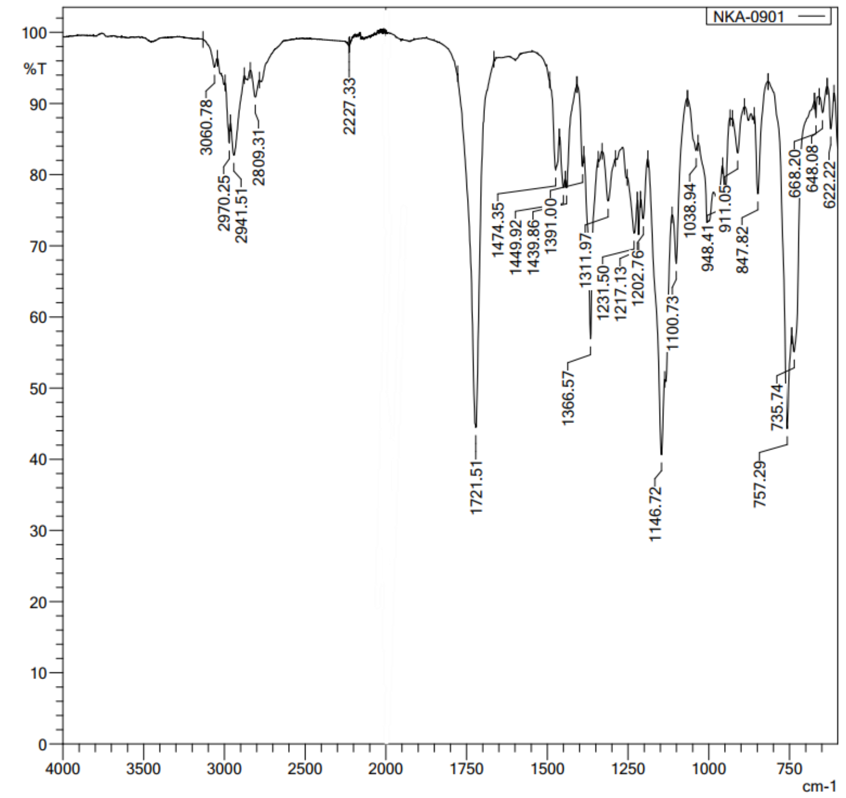

Figure S77: FTIR spectrum of **11b**.

References

(1) Kraemer, N.; Naredla, R. R.; Hoye, T. R. In Situ Allene Formation via Alkyne Tautomerization to Promote [4 + 2]-Cycloadditions with a Pendant Alkyne or Nitrile. *Organic Letters* **2022**, *24* (12), 2327-2331. https://doi.org/10.1021/acs.orglett.2c00491.

(2) Schaarschmidt, M.; Höfner, G.; Wanner, K. T. Synthesis and Biological Evaluation of Nipecotic Acid and Guvacine Derived 1,3-Disubstituted Allenes as Inhibitors of Murine GABA Transporter mGAT1. *ChemMedChem* **2019**, *14* (12), 1135-1151. https://doi.org/10.1002/cmdc.201900170.

(3) Ding, R.; He, Y.; Wang, X.; Xu, J.; Chen, Y.; Feng, M.; Qi, C. Treatment of alcohols with tosyl chloride does not always lead to the formation of tosylates. *Molecules* **2011**, *16* (7), 5665-5673. https://doi.org/10.3390/molecules16075665

(4) Qian, J.; Zhang, Y.; Zhao, W.; Hu, P. Decarboxylative halogenation of aliphatic carboxylic acids catalyzed by iron salts under visible light. *Chemical Communications* **2024**, *60* (20), 2764-2767, https://doi.org/10.1039/D3CC06149C.

(5) Peresypkin, A.; Clavel, C.; Menger, F. M. Ambidextrous 'hybrid' fluorinated zwitterionic geminis: self-assembly in both organic and aqueous media. *Mendeleev Communications* **2007**, *17* (2), 82-84. https://doi.org/10.1016/j.mencom.2007.03.009.

(6) Hodge, P.; Khoshdel, E. Conversion of alcohols into alkyl bromides using polymer-supported triphenylphosphine dibromide and polymer-supported triphenylphosphine and carbon tetrabromide. *Journal of the Chemical Society, Perkin Transactions 1* **1984**, (0), 195-198, https://doi.org/10.1039/P19840000195.

(7) Chen, J.; Lin, J.-H.; Xiao, J.-C. Halogenation through Deoxygenation of Alcohols and Aldehydes. *Organic Letters* **2018**, *20* (10), 3061-3064. https://doi.org/10.1021/acs.orglett.8b01058.

(8) Lai, J.; Xiao, X.; Shao, S.; Wang, S.; Kan, J.; Su, W. Photoinduced Transition-Metal and External Photosensitizer Free Benzylic Fluorination of Unactivated Alkylarenes. *Chemistry – A European Journal* **2024**, *30* (50), e202401669. https://doi.org/10.1002/chem.202401669.

(9) Shokat, K.; Uno, T.; Schultz, P. G. Mechanistic Studies of an Antibody-Catalyzed Elimination Reaction. *Journal of the American Chemical Society* **1994**, *116* (6), 2261-2270. https://doi.org/10.1021/ja00085a004.

(10) Yevglevskis, M.; Lee, G. L.; Sun, J.; Zhou, S.; Sun, X.; Kociok-Köhn, G.; James, T. D.; Woodman, T. J.; Lloyd, M. D. A study on the AMACR catalysed elimination reaction and its application to inhibitor testing. *Organic & biomolecular chemistry* **2016**, *14* (2), 612-622, https://doi.org/10.1039/C5OB01541C.

(11) Bera, K.; Sarkar, S.; Jana, U. Iron-catalyzed tandem carbon–carbon/carbon–oxygen bond formation/aromatization of 2'-alkynyl-biphenyl-2-carbinols: a new approach to the synthesis of substituted phenanthrenes. *Tetrahedron Letters* **2015**, *56* (2), 312-315. https://doi.org/10.1016/j.tetlet.2014.11.073.

(12) Sugimoto, A.; Yoneda, S. 9-Methylene-9,10-dihydrophenanthrene. *Journal of the Chemical Society, Chemical Communications* **1982**, (7), 376-377, 10.1039/C39820000376. DOI: 10.1039/C39820000376.

(13) Wenz, J.; Rettenmeier, C. A.; Wadepohl, H.; Gade, L. H. Catalytic C–F bond activation of geminal difluorocyclopropanes by nickel(i) complexes via a radical mechanism. *Chemical Communications* **2016**, *52* (1), 202-205, https://doi.org/10.1039/C5CC08950F.

(14) Engman, M.; Diesen, J. S.; Paptchikhine, A.; Andersson, P. G. Iridium-Catalyzed Asymmetric Hydrogenation of Fluorinated Olefins Using N,P-Ligands: A Struggle with Hydrogenolysis and Selectivity. *Journal of the American Chemical Society* **2007**, *129* (15), 4536-4537. https://doi.org/10.1021/ja0686763.

(15) Hu, D. X.; Grice, P.; Ley, S. V. Rotamers or Diastereomers? An Overlooked NMR Solution. *The Journal of Organic Chemistry* **2012**, *77* (11), 5198-5202. https://doi.org/10.1021/jo300734r.
